# Machine learning-powered estimation of malachite green photocatalytic degradation with NML-BiFeO_3_ composites

**DOI:** 10.1038/s41598-024-58976-x

**Published:** 2024-04-15

**Authors:** Iman Salahshoori, Amirhosein Yazdanbakhsh, Alireza Baghban

**Affiliations:** 1 Department of Polymer Processing, Iran Polymer and Petrochemical Institute, PO Box 14965-115, Tehran, Iran; 2 Department of Chemical Engineering, Science and Research Branch, Islamic Azad University, Tehran, Iran; 3 Department of Polymer Engineering, School of Chemical Engineering, College of Engineering, University of Tehran, Tehran, Iran; 4 Department of Process Engineering, NISOC Company, Ahvaz, Iran

**Keywords:** Dye removal, Kernel-based Gaussian process regression (GPR), Metal-incorporated bismuth ferrite (BiFeO_3_), Machine learning, Photocatalytic degradation, Wastewater treatment, Environmental sciences, Chemistry, Engineering, Materials science, Mathematics and computing

## Abstract

This study explores the potential of photocatalytic degradation using novel NML-BiFeO_3_ (noble metal-incorporated bismuth ferrite) compounds for eliminating malachite green (MG) dye from wastewater. The effectiveness of various Gaussian process regression (GPR) models in predicting MG degradation is investigated. Four GPR models (Matern, Exponential, Squared Exponential, and Rational Quadratic) were employed to analyze a dataset of 1200 observations encompassing various experimental conditions. The models have considered ten input variables, including catalyst properties, solution characteristics, and operational parameters. The Exponential kernel-based GPR model achieved the best performance, with a near-perfect R^2^ value of 1.0, indicating exceptional accuracy in predicting MG degradation. Sensitivity analysis revealed process time as the most critical factor influencing MG degradation, followed by pore volume, catalyst loading, light intensity, catalyst type, pH, anion type, surface area, and humic acid concentration. This highlights the complex interplay between these factors in the degradation process. The reliability of the models was confirmed by outlier detection using William’s plot, demonstrating a minimal number of outliers (66–71 data points depending on the model). This indicates the robustness of the data utilized for model development. This study suggests that NML-BiFeO_3_ composites hold promise for wastewater treatment and that GPR models, particularly Matern-GPR, offer a powerful tool for predicting MG degradation. Identifying fundamental catalyst properties can expedite the application of NML-BiFeO_3_, leading to optimized wastewater treatment processes. Overall, this study provides valuable insights into using NML-BiFeO_3_ compounds and machine learning for efficient MG removal from wastewater.

## Introduction

Water pollution, the introduction of harmful substances into water bodies like rivers, lakes, and oceans, stems from industrial processes, agriculture, urban runoff, and sewage disposal^1–12^. This pollution jeopardizes human health, ecosystems, economic activities, and access to clean drinking water^13,14^. Addressing water pollution is crucial for environmental justice, combating climate change, and sustaining a healthy future^15–18^. Pollutants such as organic compounds, pharmaceuticals, and chemicals harm aquatic life and water quality, highlighting the need for effective management and regulation to protect both the environment and human health^19–24^.

Traditional wastewater treatment can struggle with eliminating persistent organic pollutants due to their resistance to conventional methods, complex molecular structures, and the potential formation of harmful byproducts during treatment^25–27^. To overcome these limitations, advanced technologies like adsorption^28,29^, membrane filtration^30^, biological treatment^31^, and Advanced Oxidation Processes (AOPs)^32,33^ are being developed and tailored to specific pollutant profiles with ongoing regulatory updates to protect the environment and public health^34,35^. Each method carries distinct benefits and drawbacks. Adsorption efficiently eliminates heavy metals, organic compounds, and dye with minimal maintenance; yet, it demands expensive adsorbent material replacement and lacks universality in pollutant removal. Membrane filtration is effective but entails high costs due to maintenance and fouling concerns. Biological treatments, such as activated sludge and trickling filters, are effective but require ample space and meticulous handling. The appropriate system selection depends on factors like pollutant type, effluent quality, economic feasibility, and environmental repercussions^36–41^.

AOPs, notably photocatalysis, are recognized for effectively combating toxic organic pollutants in environmental and wastewater treatment^42^. Photocatalysis offers eco-friendly, efficient, and cost-effective solutions by utilizing natural energy sources to generate reactive species for sustainable water treatment and environmental remediation^43^.

Bismuth ferrite (BiFeO_3_), a magnetic perovskite, shows promise in photocatalysis due to its low bandgap energy, thermal and chemical stress resistance, non-toxicity, and visible light responsiveness^44,45^. However, rapid recombination of photogenerated charge carriers limits its practical use^46^. Addressing this challenge involves strategically incorporating noble metals like silver (Ag), platinum (Pt), and palladium (Pd) as co-catalysts on BiFeO_3_’s surface^47^. This prevents electron loss to noble metals, enhancing electron management and catalytic efficiency^21,48^. The accumulation of electrons at noble metal surfaces facilitates reduction reactions, while BiFeO_3_’s valence band holes generate reactive hydroxyl radicals, crucial for chemical transformations^46,49^. Composite materials of BiFeO_3_ with noble metals efficiently degrade organic pollutants, outperforming BiFeO_3_ alone^46,48^.

The efficiency of a photocatalyst in degrading pollutants is influenced by various factors, including the catalyst’s properties (such as pore volume and surface area), pollutant characteristics (like composition and concentration), competing compounds, and reaction conditions (e.g., time, pH, catalyst dosage, and light intensity)^50^. Achieving optimal conditions through experimentation can take days to months, especially for degrading MG dyes. However, the standard empirical method may not capture complex interactions among factors affecting efficiency^51,52^. Many photocatalytic materials, especially those responsive to visible light like BiFeO_3_, demonstrate superior performance compared to TiO_2_ but are associated with high costs. Developing an analytical, data-centric template could enhance the optimization of the photocatalytic process, improving its economic feasibility^53^. Such an approach would consider the interconnectedness of factors influencing water quality, streamlining optimization efforts and contributing to the process’s economic viability.

Machine learning (ML) strategically deploys mathematical algorithms to build predictive models from datasets, aiming to inform decisions across qualitative and quantitative dimensions^54^. Until now, the wastewater treatment domain has effectively implemented a variety of basic ML algorithms^55,56^. Gaussian Process Regression (GPR) offers a distribution-free approach, estimating values and uncertainty in predictions, which is ideal for the complex relationships encountered in wastewater treatment^56^.

Scientific studies have applied ML models to wastewater datasets to predict water quality, assess environmental impact, and evaluate treatment performance^57–62^. However, these studies often need more substantial evidence regarding the suitability of the chosen ML algorithm and utilize limited datasets, typically with few input variables. ML approaches may vary in function, leading to variability in estimate precision^58,63^. Therefore, selecting the right ML approach for predicting pollutant degradation in wastewater is crucial.

This study introduces a novel approach to predicting and comparing MG dye photodegradation productivity using a dataset of 1200 observations and ten input factors. It employs Gaussian Process Regression with four kernel functions and optimizes photocatalytic procedure settings across these factors. The research meticulously ensures the accuracy of ML models and analyzes the interdependencies among process factors for MG dye degradation. Post-processing techniques, including sensitivity analysis, evaluate feature effectiveness and shed light on individual input variables’ significance in photodegradation. This multifaceted approach advances the understanding of wastewater treatment and provides a framework for future research in predictive modelling and process optimization.

## Computational methodology

### Methodology

**Figure 1:**
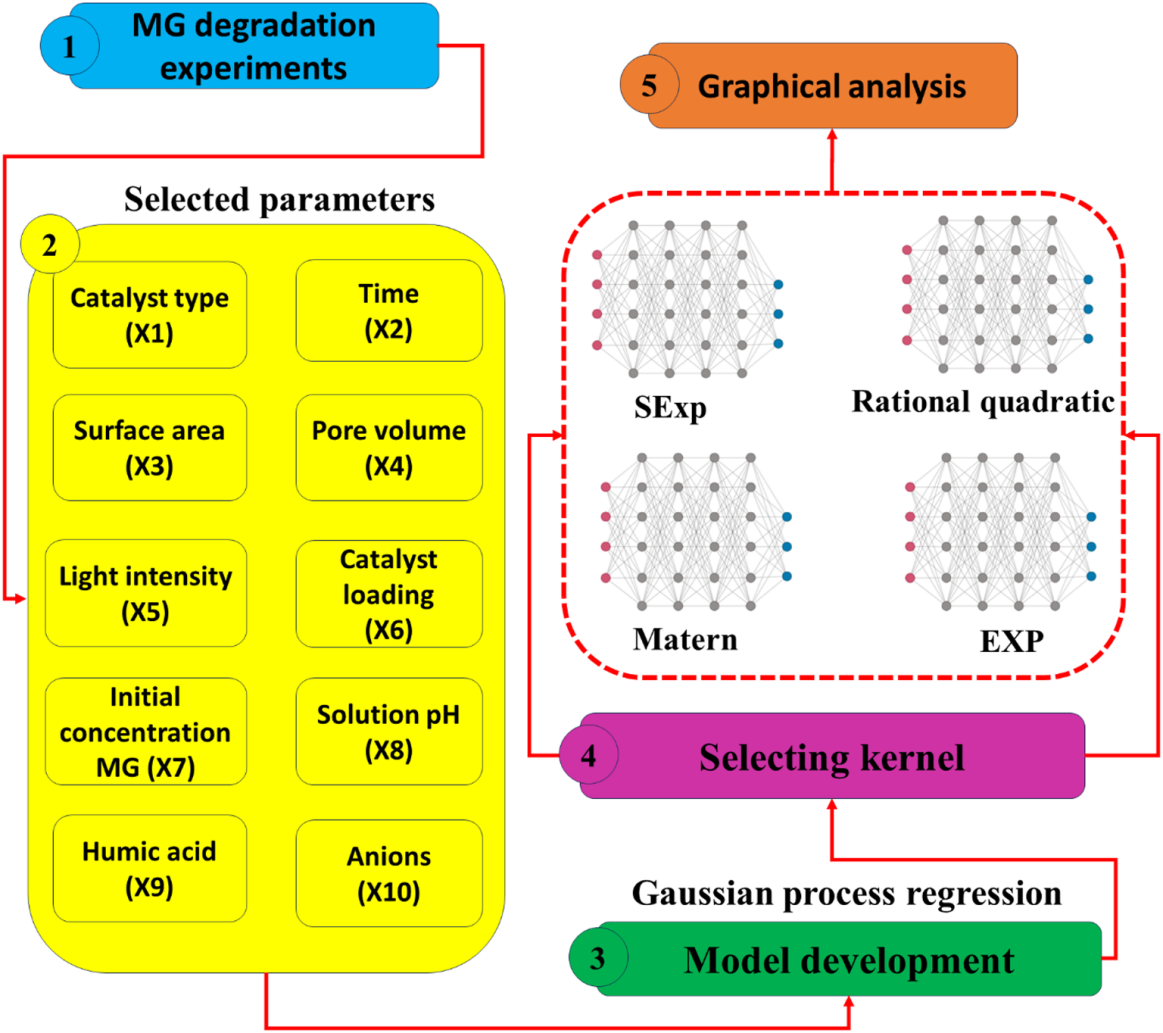
Approach, gathering information, modelling, and additional processing schematic.

The methodology used in this study for modelling and optimizing MG dye photocatalysis using NML-BiFeO_3_ compounds is depicted in Fig. 1. This methodology draws on insights from our previous research. Figure 1 illustrates that the study is conducted through three distinct stages. The initial step involves selecting ten parameters that significantly influence degradation efficiency, followed by designing and collecting 1200 data points through experimentation. In the second phase, an extensive comparison of four different kernel functions in the GPR model is conducted to identify the most suitable configurations for accurately predicting the efficiency of MG dye elimination throughout the photocatalytic procedure. Following this, the development of photocatalytic behaviour is determined by leveraging the four models exhibiting superior performance with higher R-squared values and lower error rates.

### Data preparation

We used 1200 data points in this investigation, which were obtained from a previous photocatalytic study^64^. Table S1 comprehensively lists the providers, concentrations, chemical formulas, labels, and intended applications of all chemicals used in this present investigation.

This study included a comprehensive set of 10 distinct features, meticulously selected because of their relevance and potential influence on the photocatalytic process under examination. These features encompassed a variety of parameters, including the type of catalyst employed, the duration of the experiment (in minutes), the surface area of the catalyst material (expressed in m^2^/g), the pore volume of the material (in cm^3^/g), the intensity of the illumination (in watts), the quantity of catalyst loaded into the system (in g/L), in-solution MG dye concentration (in mg/L), the pH of the solution, the concentration of humic acid (in mg/L), and the presence or absence of specific anions. The output variable was the efficacy of MG dye degradation.

Within the data preparation phase, particular attention focused on two categorical input variables: the anions and catalyst types. We employed a new strategy to convert these attributes into numerical representations. To characterize catalyst types, a linear combination of the normalized surface area and pore volume of the catalysts was chosen. In addition, to characterize anion types, the normalized molecular weight of each anion was considered. It is worth noting that the normalization was carried out within the range of 0 to 1.

This conversion was deemed essential to ensure that the data met the stringent numerical prerequisites of ML algorithms, enabling seamless integration into the subsequent analytical processes. Preceding the commencement of machine learning model construction, a pivotal procedural step entailed randomly partitioning the dataset into two discrete subsets. Explicitly, 75% of the dataset was earmarked for utilization as the training dataset, whereas the remaining 25% was earmarked to serve as the test dataset. This division’s rationale was to facilitate a comprehensive evaluation of the machine learning models post-training. This partitioning strategy ensured that the models were rigorously assessed on unseen data, gauging their generalization capabilities beyond the training phase.

### Gaussian process regression (GPR)

A powerful and well-structured machine learning approach, the GPR model, is well-regarded for its probabilistic and nonparametric characteristics. It can handle complex problems that involve non-linear relationships^65^. A key feature of this approach is the use of Gaussian processes for regression tasks. A significant aspect of its attractiveness arises from its capacity to efficiently incorporate uncertainty within its computational framework^53^.

In the context of GPR modelling, it is conventional to utilize two separate datasets: one allocated explicitly for training purposes (L) and another intended for testing (T). These datasets, T and L, are selected at random and comprise sets ${\left\{{x}_{L\cdot i}\cdot {y}_{L.i}\right\}}_{i=1}^{n}$, and ${\left\{{x}_{T\cdot i}\cdot {y}_{T\cdot i}\right\}}_{i=1}^{n}$, where 'x' denotes the entered parameters and 'y' corresponds to the associated result factors. The following Equation establishes the basis of GPR modelling:

1 $${y}_{L.i}=f\left({x}_{L\cdot i}\right)+{\varepsilon }_{L\cdot i} \cdot \quad  i=1.2.3.\ldots .n$$

2 $$\varepsilon \sim N(0\cdot {\sigma }_{noise}^{2}{I}_{n})$$

Here, ‘xL’ signifies the individual factors, whereas ‘yL’ signifies the consequences linked to the training data sets. Furthermore, ‘ε’ serves as the notation for observation noise, ‘σ^2^_noise_’ represents the noise variance, and ‘In’ denotes the unit array in this context. In the same vein, we can articulate the following for the test dataset:

3 $${y}_{T\cdot i}=f\left({x}_{T\cdot i}\right)+{\varepsilon }_{T\cdot i} \cdot \quad  i=1.2.3.\ldots . n$$

The symbols retain their previously defined meanings, but in this case, they pertain to the test dataset. Consequently, the Gaussian noise model links each computed ‘y’ value to the corresponding ‘f(x)’ function under consideration. As postulated by the GPR paradigm, ‘f(x)’ assumes the role of a stochastic function, and its characterization is contingent upon the concurrent utilization of the mean function’ m(x)’ and the covariance function’ k(x, x′),’ regularly recognized as kernel functions.

4 $$f\left({x}_{L\cdot i}\right) \sim GP(m\left(x\right)\cdot  k(x\cdot {x}^{\prime}))$$

It is possible to find the mean function “m(x)” by using specified basis functions; nonetheless, it is commonly approximated as zero for simplification and computational convenience^66^.

5 $$f\left({x}_{L.i}\right) \sim GP(0\cdot  k(x\cdot {x}^{\prime}))$$

Merging Eqs. (1) and (5) allows us to determine the ‘y.’ distribution.

6 $$y \sim N(0\cdot  k\left(x\cdot {x}^{\prime}\right)+{\sigma }_{noise}^{2}{I}_{n})$$

Concluding the previously mentioned criteria and variables, the following deductions can be made:

7 $$\left[\begin{array}{c}\underset{{f}_{L}}{\to }\\ \underset{{f}_{T}}{\to }\end{array}\right] \sim N\left(0. \left[ \begin{array}{cc}k\left({x}_{L}.{x}_{L}\right)& k\left({x}_{L}.{x}_{T}\right)\\ k\left({x}_{T}.{x}_{L}\right)& k\left({x}_{T}.{x}_{T}\right)\end{array}\right]\right)$$

8 $$\left[\begin{array}{c}\underset{{\varepsilon }_{L}}{\to }\\ \underset{{\varepsilon }_{T}}{\to }\end{array}\right] \sim N\left(0. \left[\begin{array}{cc}{\sigma }_{noise}^{2}{I}_{n}& 0\\ 0& {\sigma }_{noise}^{2}{I}_{n}\end{array}\right]\right)$$

Incorporating the most recent pair of equations, we can derive the subsequent Gaussian expression:

9 $$\left[\begin{array}{c}\underset{{y}_{L}}{\to }\\ \underset{{y}_{T}}{\to }\end{array}\right] \sim N\left(0. \left[\begin{array}{cc}k\left({x}_{L}.{x}_{L}\right)+{\sigma }_{noise}^{2}{I}_{n}& k\left({x}_{L}.{x}_{T}\right)\\ k\left({x}_{T}.{x}_{L}\right)& k\left({x}_{T}.{x}_{T}\right)+{\sigma }_{noise}^{2}{I}_{n}\end{array}\right]\right)$$

By applying the Gaussian conditioning principle, we can acquire the distribution for the variable’ y_T_.’:

10 $$\left({y}_{T}|{y}_{L}\right)\sim N\left({\mu }_{T}.{\Sigma }_{T}\right)$$

11 $${\Sigma }_{T}=k\left({x}_{T}.{x}_{T}\right)=k\left({x}_{T}.{x}_{T}\right)+{\sigma }_{noise}^{2}{I}_{n}-{k\left({x}_{T}.{x}_{L}\right)\left(k\left({x}_{L}.{x}_{L}\right)+{\sigma }_{noise}^{2}{I}_{n}\right)}^{-1}k({x}_{L}.{x}_{T})$$

12 $${\mu }_{T}=m\left(\underset{{y}_{T}}{\to }\right)=k\left({x}_{T}.{x}_{L}\right){\left(k\left({x}_{L}.{x}_{L}\right)+{\sigma }_{noise}^{2}{I}_{n}\right)}^{-1} \underset{{y}_{T}}{\to }$$

In this scenario, Σ_T_ represents the covariance, while μ_T_ signifies the mean value. A GPR model’s predictive power and resilience are influenced by kernel function with a non-singular symmetric template. Four options— Squared exponential, Exponential, Matern, and Rational quadratic—have been selected to identify the best-suited kernel function. Presented below are the selected kernel functions:

Rational quadratic kernel function:

13 $${k}_{RQ}\left(x.{x}^{\prime}\right)={\sigma }^{2}{\left(1+\frac{x-{x}^{{\prime} 2}}{2a{\ell}}\right)}^{-a}$$

Matern kernel function:

14 $${k}_{M}\left(x.{x}^{\prime}\right)={\sigma }^{2}\frac{{2}^{1-v}}{\Gamma \left(v\right)}{\left(\sqrt{2v}\frac{x-{x}^{\prime}}{{\ell}}\right)}^{v}{K}_{v}\left(\sqrt{2v}\frac{x-{x}^{\prime}}{{\ell}}\right)$$

Squared Exponential kernel function:

15 $${k}_{SE}\left(x.{x}^{\prime}\right)={\sigma }^{2}exp\left(-\frac{x-{x}^{{\prime} 2}}{{{\ell}}^{2}}\right)$$

Exponential kernel function:

16 $${k}_{E}\left(x.{x}^{\prime}\right)={\sigma }^{2}exp\left(-\frac{x-{x}^{\prime}}{{\ell}}\right)$$

Within this context, the parameters ℓ, σ^2^, σ, and α correspond to length scale, variance, amplitude and scale mixture, respectively. Furthermore, the symbols v, Γ, and Kv were employed to signify a positive parameter, gamma function, and modified Bessel function, respectively.

### Performance metrics

The performance of the established models depended on the data quality and input factors. To measure model performance, a set of statistical measures were employed: the coefficient of determination (R^2^), root-mean-square error (RMSE), and mean absolute error (MAE). The subsequent equations delineate these parameters:

17 $$MAE= \frac{\left[\sum_{i=1}^{n}\left|{o}_{i}-{p}_{i}\right|\right]}{n}$$

18 $$RMSE= \sqrt{\frac{\left[\sum_{i=1}^{n}{\left({o}_{i}-{p}_{i}\right)}^{2}\right]}{n}}$$

19 $${R}^{2}1-\frac{\sum {\left({o}_{i}-{p}_{i}\right)}^{2}\left({p}_{i}-\overline{p }\right)}{\sum {\left({o}_{i}-\overline{o }\right)}^{2}\sum {\left({p}_{i}-\overline{p }\right)}^{2}}$$

Here, "n" signifies the total number of samples considered. "oi" represents the observed removal efficiencies, whereas "pi" stands for the calculated removal efficacies. Furthermore, "p" holds the significance of being the mean value derived from all anticipated effectiveness quantities.

## Results and discussion

### Model development and testing

This study employed MATLAB software version 2018 to develop GPR models for predicting MG dye photocatalytic degradation. Table 1 compares our findings with previous research on organic pollutant degradation. The GPR models developed here achieved superior R-squared values and lower MAE and RMSE values compared to a significant portion of the existing literature. High R-squared values indicate strong agreement between predicted and experimental degradation values, validating the effectiveness of the models.

**Table 1:** The numerical measures associated with the GPR models are indicated in this research.

Model	Group	R^2^	MRE (%)	MSE	RMSE	STD
Matern	Train data	1.000	0.005	4.83768E-06	0.0022	0.0016
	Test data	1.000	0.009	6.08233E-06	0.0025	0.0018
	Total data	1.000	0.006	5.14884E-06	0.0025	0.0016
Exponential	Train data	1.000	0.120	0.004360388	0.0660	0.0607
	Test data	1.000	0.230	0.00451456	0.0672	0.0624
	Total data	1.000	0.157	0.004398931	0.0672	0.0611
Squared exponential	Train data	1.000	0.363	0.039625283	0.1991	0.1667
	Test data	1.000	0.538	0.060361797	0.2457	0.2134
	Total data	1.000	0.435	0.044809411	0.2457	0.1795
Rational quadratic	Train data	1.000	1.413	0.269581839	0.5192	0.4295
	Test data	1.000	3.356	0.308675869	0.5556	0.4671
	Total data	1.000	2.007	0.279355346	0.5556	0.4390

We examined error characteristics (STD, RMSE, MSE, MRE) to assess the training performance of the recommended GPR models. The error metrics indicate that the models effectively captured patterns and trends in the training data. Notably, the GPR model with an exponential kernel demonstrated excellent accuracy in predicting MG dye degradation for unseen data. Its high R-squared value (1.0) and low error metrics highlight its superior predictive capabilities.

This exceptional performance suggests the model's effectiveness in handling the complexities of MG dye photocatalytic degradation in wastewater, with potential applications in carbon capture and utilization. The multifaceted nature of the experimental design and the inclusion of diverse input features contribute to the richness and comprehensiveness of this study, leading to a more meaningful understanding of the underlying phenomena. Consequently, the GPR model's predictive performance emerges as a more reliable and suitable solution for addressing real-world challenges in this domain.

**Figure 2:**
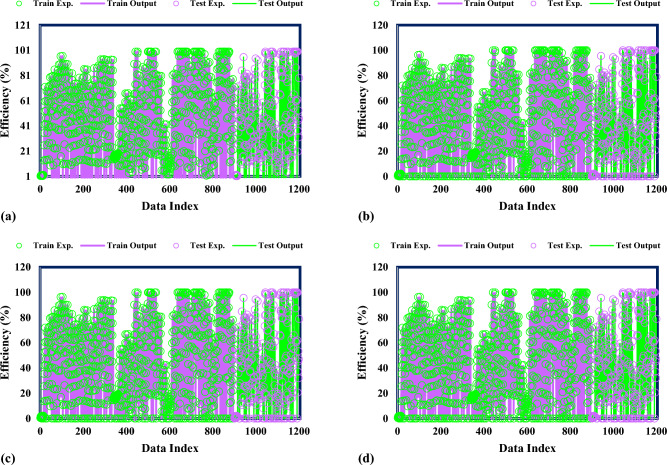
Findings from experiments and the kernel-based GPR algorithm for (**a**) Matern, (**b**) Exponential, (**c**) Squared exponential, (**d**) Rational quadratic.

The correctness of the proven models is further validated by the simultaneous presentation of the anticipated and experimental values for the photocatalytic degradation of the MG dye in Fig. 2. Upon careful examination of the data, it is evident that the photocatalytic destruction of the experimental MG dye aligns with the many GPR models. This agreement precisely demonstrates the models’ ability to predict the MG dye photocatalytic degradation in NML-BiFeO3 composites. A broad investigation of the presented models shows a strong match between the anticipated and observed MG dye photocatalytic degradation rates.

This tight correlation shows that GPR models can accurately predict MG dye photocatalytic degradation in NML-BiFeO_3_. The algorithms' exact alignment between predicted and observed values shows their capacity to precisely capture photocatalytic degradation events, which could impact wastewater treatment. The remarkable effectiveness of GPR models enhances the field of model prediction as researchers gain more confidence in using these models to make predictions about MG dye removal efficiency and improve processes linked to photocatalytic degradation.

**Figure 3:**
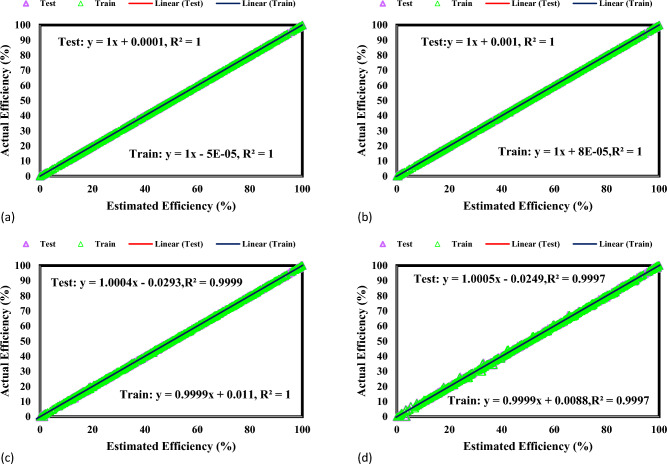
Graphs depicting the Kernel-based GPR system for (**a**) Matern, (**b**) Exponential, (**c**) Squared exponential, (**d**) Rational quadratic.

The visual representation in Fig. 3 illustrates the prediction accuracy of GPR models in the process of MG dye photocatalytic degradation compared to the data collected from experiments. The graph demonstrates an important link above 1.000 between the predicted and experimental outcomes.

The exact synchronization of the matching lines with the 45° line indicates the systems' accuracy in detecting complicated degradation trends. The precise positioning along the dividing line, especially in the GPR model using the Matern kernel function, achieves an impeccable correlation value of 1. The graph is an essential tool for evaluating the accuracy of GPR models in forecasting the photocatalytic degradation of MG dye within the NML-BiFeO_3_ composite. Researchers gain vital knowledge on the accuracy of models, which helps improve wastewater treatment technologies and informs choices in academic and commercial contexts. The excellent accuracy shown by the Matern kernel-equipped GPR model distinguishes it as a noteworthy instrument for forecasting MG dye photocatalytic degradation with unprecedented precision.

**Figure 4:**
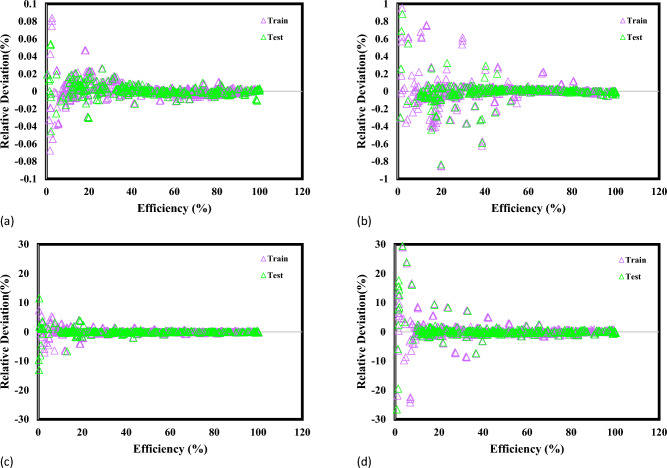
A comparison of the prediction performance of GPR models using (**a**) Exponential, (**b**) Matern, (**c**) Squared exponential, and (**d**) Rational quadratic versus empirical information.

Figure 4 illustrates and communicates crucial information about the predictive efficacy of GPR models in the context of MG dye photocatalytic degradation. The figure prominently displays the differences between experimentally measured MG dye photocatalytic degradation values and the corresponding estimated values obtained from GPR models. The accuracy of different GPR models is evaluated based on their ability to predict MG dye photocatalytic degradation. The Rational Quadratic and Squared Exponential kernel functions are highlighted for their remarkable accuracy. The relative deviation points for these models are reported to be below 30%, demonstrating a tight correlation across expected and investigational results. The relative deviation points of the Exponential kernel function are less than 1%, while the Matern kernel function stands out for its superior accuracy, showcasing absolute deviation points below 0.1%. This suggests a high precision in capturing the underlying behavior of photocatalytic degradation. The accuracy and reliability of the GPR models, especially those using specific kernel functions, are emphasized. This supports their credibility for predicting MG dye photocatalytic degradation in the NML-BiFeO_3_ composite. The discoveries indicate that this information could help scholars choose the most appropriate GPR systems for different purposes, particularly in wastewater treatment and employment inquiry. The overall aim is to contribute to sustainable solutions by improving the understanding and prediction of dye pollutant emissions.

**Figure 5:**
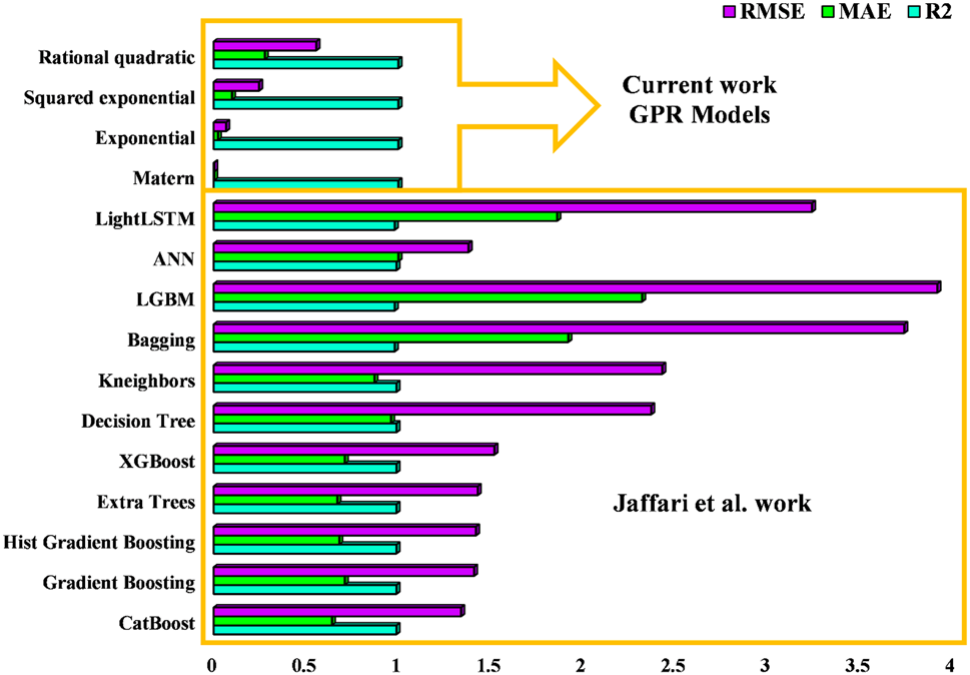
Statistical comparison of the current GPR models with the Jaffari et al.^64^ models.

The insights from Fig. 4 regarding GPR models' MG dye photocatalytic degradation predictions are significant. The emphasis on kernel functions and accuracy levels helps scientists select the best models for specific functions, boosting wastewater treatment and sustainable solutions research. Figure 5 compares the current four GPR models with the models developed by Jaffari et al.^64^ to estimate the efficiency of MG photocatalytic degradation with NML-BiFeO_3_ composites. As can be seen, the current models achieve higher accuracy compared to the literature models, evidenced by lower errors and higher R-squared values.

### Sensitivity analysis

Sensitivity inquiry is conventionally carried out to explore the impact of input factors on the resultant output quantity^67^. As part of this in-depth analysis, it is imperative to consider the relevance factor, represented as ‘r,’ which serves as the primary indicator of the input parameter exerting the most significant impact on MG photocatalytic degradation with NML-BiFeO_3_ composites. This influential parameter can be quantified using the ensuing Equation:

20 $$r=\frac{{\sum }_{i=1}^{n}\left({X}_{k.i}-{\overline{X} }_{k}\right)\left({Y}_{i}-\overline{Y }\right)}{\sqrt{{\sum }_{i=1}^{n}{\left({X}_{k.i}-{\overline{X} }_{k}\right)}^{2}{\sum }_{i=1}^{n}{\left({Y}_{i}-\overline{Y }\right)}^{2}}}$$

**Figure 6:**
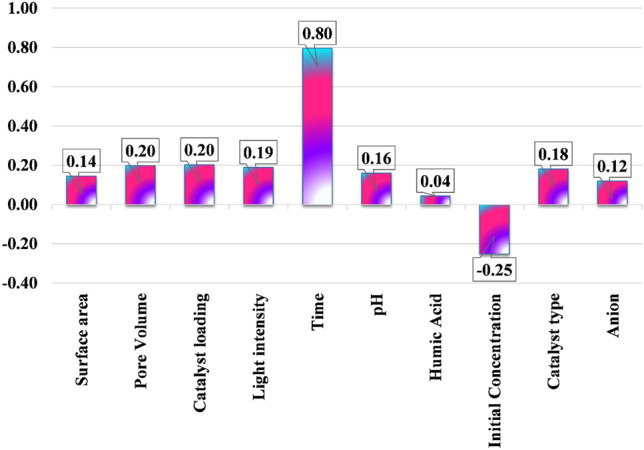
MG photocatalytic degradation with NML-BiFeO_3_ composites input parameter analysis.

Within the presented framework, a variety of notations are employed, each possessing specific meanings: ${X}_{k.i}$ is indicative of the ‘*k*th’ input parameter, ${\overline{X} }_{k}$ represents the average value of input parameters, *Y*_*i*_ signifies the ‘*i*th’ output, $\overline{Y }$ denotes the average of outputs, and ‘*n*’ denotes the total quantity of data points included in the analysis. Typically, the ‘*r*’ value exhibits variation within the range of −1 to + 1. It is worth emphasizing that the absolute value of ‘*r*’ measures how each input variable affects the output variable. A higher absolute value of ‘*r*’ signifies a more pronounced correlation between each input and its output. Notably, negative values represent a situation where higher input values correspond to lower output values, while positive values indicate that higher input values are associated with higher output values^68^. The work includes a visually captivating representation in Fig. 6, which is significant.

The sensitivity study illuminates the complex interplay between input parameters and MG photocatalytic degradation, successfully identifying the crucial factors that contribute to the process.

Analyzing feature significance with the GPR model allows us to comprehend the impact of operational factors on the photodegradation estimate of MG dye. Our investigation focused on understanding how various input features influenced the GPR model's overall accuracy. Figure 6 presents the resulting assessment of the relative importance of these input features.

Pore volume and catalyst loading contribute 20% each, followed by light intensity at 19%. Catalyst type contributes 18%, followed by the pH of the solution at 16%. Anion type contributes 12%, surface area contributes 14%, and humic acid concentration contributes 4%. The most important factor in this situation is the photocatalytic process's time..

Notably, the gap in relative significance between the most critical factor, represented by time, and the least significant factor, exemplified by humic acid concentration, exceeds 80%. It becomes apparent that the degradation of MG dye was markedly impacted by the input factors linked to the circumstances of the photocatalytic process, as illustrated in the inset of Fig. 6. Further scrutiny of the GPR model involved a thorough investigation through a permutation significance assessment. This method discerns the decrement in model effectiveness resulting from the random reshuffling of an individual feature^69^. This procedure creates a disconnect between the input attributes and the effectiveness of MG dye degradation, leading inexorably to a downturn in the model's performance rating, thereby underscoring the model's dependence on these precise attributes.

### Outlier detection

Data points deemed outliers or giving rise to suspicion demonstrate dissimilar behaviour in comparison to the remaining data, and this disparity is frequently attributed to experimental irregularities or instrumental inaccuracies. To enhance the efficiency of the determined model and prevent erroneous analysis, it is imperative to identify and address potentially problematic data within the dataset. To streamline this procedure, we employ the Leverage method, a technique in which the Hat matrix is precisely articulated as follows:

21 $$H=U{({U}^{T}U)}^{-1}{U}^{T}$$

U is characterized as a matrix with sizes i*j, where *i* denotes the parameter count, and *j* represents the number of training data points. A visual depiction known as a Williams plot is produced to evaluate the veracity of the information. This analysis involves plotting standardized residuals against Hat values, allowing for a comprehensive evaluation; any data falling outside a designated region is considered potentially questionable. This dependable zone is a narrow space encompassing Hat values and residuals with a standard deviation between −3 and 3, ranging from 0 to the limits of critical leverage. The calculation for the limits of critical leverage is determined as follows^70,71^:

22 $${H}^{*}=\frac{3(j+1)}{i}$$

**Figure 7:**
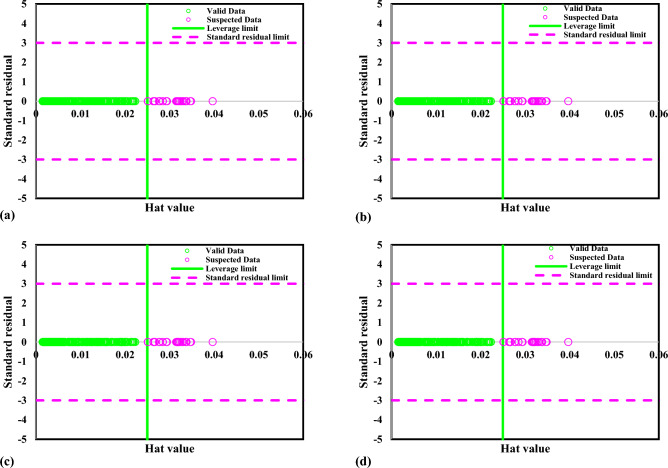
William’s MG photocatalytic degradation data bank visualization for outliers for Kernel-based GPR model of (**a**) Matern, (**b**) Exponential, (**c**) Squared exponential, (**d**) Rational quadratic.

Drawing insights from William’s plot of the MG photocatalytic degradation data bank (Fig. 7), one can infer that a significant portion of the data employed in the analysis is deemed reliable. To provide a more detailed breakdown, out of a total of 1200 data points, only 71, 68, 69, and 66 outliers were identified for the GPR-Rational quadratic models, GPR-Squared Exponential, GPR-Exponential, and GPR-Matern, respectively.

### Implications and drawbacks of the current study

The utilization of NM-BiFeO_3_ composites reveals considerable promise as a viable option for catalyzing the degradation of organic contaminants in aqueous environments. Experimental measurements involving controlled variables are usually employed in the conventional approach to establish the correlation between degradation effectiveness and reaction settings. However, these hands-on experiments often come with high costs, consume significant time, and need help achieving broad approval. This study employed four proficient ML models to illustrate the performance of MG dye photodegradation. This highlights a notable potential for promptly forecasting empirical outcomes using predetermined settings. The study also identified the key attributes of a photocatalyst’s surface characteristics. It assessed their influence on the material’s effectiveness in degrading organic pollutants and facilitating selective conditions during photocatalytic reactions for treating organic wastewater. Applying this method will substantially diminish the necessity for extensive experimental exploration, resulting in cost savings and expediting the utilization of NML-BiFeO_3_ compounds in organic wastewater treatment.

The current investigation underscores ML as a promising avenue for forecasting NML-BiFeO_3_-assisted photodegradation of MG dye compounds under controlled parameters. However, it is important to acknowledge limitations. Photocatalytic performance can be significantly influenced by various other factors, including temperature, pore volume, and catalyst loading. Additionally, this study does not account for the presence of multiple organic contaminants within a real-world wastewater treatment scenario. Fluctuations in these parameters could introduce discrepancies in the model, modify the significance of features, and limit the model's generalizability due to the absence of experimental data for these conditions. Future research will prioritize understanding the influence of these variables on the NML-BiFeO_3_ photocatalytic process. The model will be further refined by incorporating additional data to enhance its precision and broaden its applicability to a wider range of organic pollutants. It is important to note that different organic pollutants may behave differently within photocatalytic systems. Therefore, further exploration using readily available datasets and a comprehensive investigation of these variables' influence on the photocatalytic breakdown of various organic pollutants in wastewater is warranted.

## Conclusions

In this study, we investigated the potential of various Gaussian process regression (GPR) models for predicting malachite green (MG) dye degradation using noble metal-incorporated bismuth ferrite (BiFeO_3_) (NML-BiFeO_3_) photocatalysts. The GPR models significantly outperformed existing methods in predicting MG degradation efficacy, achieving exceptional accuracy. This high accuracy is validated by the high R^2^ values and low error metrics. The exponential kernel-based GPR model demonstrated the most exceptional performance, with a near-perfect R^2^ value of 1.0 and minimal errors. This establishes its exceptional suitability for forecasting MG photocatalytic degradation in wastewater treatment. The close alignment between predicted and experimental results underscores the reliability of the GPR models in estimating degradation rates. This precision strengthens the foundation for utilizing GPR models to guide decision-making and optimize processes related to MG dye degradation.

Notably, the Rational Quadratic and Squared Exponential kernel models exhibited significant accuracy, with deviations below 30%. The Exponential kernel achieved exceptional precision with less than 1% deviation, while the Matern kernel surpassed all others with a deviation of less than 0.1%. These findings highlight the remarkable accuracy of these models, particularly those employing specific kernels, for predicting MG dye degradation using NML-BiFeO_3_ photocatalysts. These insights empower researchers to select the most appropriate GPR systems for wastewater treatment applications, ultimately contributing to advancements in sustainability efforts.

Furthermore, the study identified crucial input factors influencing MG photocatalytic degradation through a comprehensive sensitivity analysis. The direct correlation between the input parameters and the degradation process reveals the complex interplay between these factors. Analyzing feature significance using the GPR model revealed that process time is the most influential factor, followed by pore volume, catalyst loading, light intensity, catalyst type, pH, anion type, surface area, and humic acid concentration.

The reliability of the data employed in the analysis is further supported by insights gleaned from William's plot. Notably, a minimal portion of the 1200 data points (ranging from 66 to 71 data points depending on the GPR model) were identified as outliers. This signifies the robustness of the data employed for model development.

In conclusion, this study demonstrates the promising potential of NML-BiFeO_3_ composites for catalyzing the degradation of organic contaminants in wastewater. The utilization of GPR models for forecasting MG dye photodegradation offers a powerful tool for rapid and efficient prediction of empirical outcomes. Identifying key catalyst surface properties can significantly expedite the application of NML-BiFeO_3_ in organic wastewater treatment, leading to reduced costs and streamlined experimental procedures. Future research endeavors should explore the incorporation of additional variables to further enhance model accuracy and broaden applicability..

### Supplementary Information

Supplementary Information.

## Data Availability

All the data used for model development provides in supplemental information.
