# Supplementary material for: Machine learning-powered estimation of malachite green photocatalytic degradation with NML-BiFeO3 composites
Source: Sci Rep. 2024 Apr 15;14:8676. doi: 10.1038/s41598-024-58976-x (PMC11018770; doi:10.1038/s41598-024-58976-x)
Supplement: Supplementary file 1 — Supplementary Information. [file 41598_2024_58976_MOESM1_ESM.docx]

**Machine Learning-Powered Estimation of Malachite Green Photocatalytic Degradation with NML-BiFeO_3_ Composites**

*Iman Salahshoori^1,2,^Amirhosein Yazdanbakhsh^3^ and Alireza Baghban^4,*^*

*^1^Department of Polymer Processing, Iran Polymer and Petrochemical Institute,* *PO Box 14965-115, Tehran, Iran*

*^2^Department of Chemical Engineering, Science and Research Branch, Islamic Azad University, Tehran, Iran*

*^3^Department of Polymer Engineering, School of Chemical Engineering, College of Engineering, University of Tehran, Tehran, Iran*

*^4^Department of Process Engineering, NISOC Company, Ahvaz, Iran*

***^*^Corresponding author:*** [*Alireza_baghban@ut.ac.ir*](mailto:Alireza_baghban@ut.ac.ir) *(A. Baghban)*

Table S1: Details of data used for model development

| Catalyst type | Surface area | Pore Volume | Catalyst loading (g/L) | Light intensity (W) | time (min) | Solution pH | HA (mg/L) | Ci (mg/L) | Cf (mg/L) | Efficiency (%) | Anions |
| --- | --- | --- | --- | --- | --- | --- | --- | --- | --- | --- | --- |
| no catalyst | 0 | 0 | 0 | 105 | 0 | 5.45 | 0 | 10 | 10 | 0 | without Anion |
| no catalyst | 0 | 0 | 0 | 105 | 30 | 5.45 | 0 | 10 | 9.98 | 0.2 | without Anion |
| no catalyst | 0 | 0 | 0 | 105 | 60 | 5.45 | 0 | 10 | 9.96 | 0.4 | without Anion |
| no catalyst | 0 | 0 | 0 | 105 | 90 | 5.45 | 0 | 10 | 9.94 | 0.6 | without Anion |
| no catalyst | 0 | 0 | 0 | 105 | 120 | 5.45 | 0 | 10 | 9.87 | 1.3 | without Anion |
| no catalyst | 0 | 0 | 0 | 105 | 150 | 5.45 | 0 | 10 | 9.83 | 1.7 | without Anion |
| no catalyst | 0 | 0 | 0 | 105 | 180 | 5.45 | 0 | 10 | 9.82 | 1.8 | without Anion |
| no catalyst | 0 | 0 | 0 | 105 | 210 | 5.45 | 0 | 10 | 9.82 | 1.8 | without Anion |
| no catalyst | 0 | 0 | 0 | 105 | 240 | 5.45 | 0 | 10 | 9.81 | 1.9 | without Anion |
| no catalyst | 0 | 0 | 0 | 105 | 270 | 5.45 | 0 | 10 | 9.8 | 2 | without Anion |
| no catalyst | 0 | 0 | 0 | 105 | 0 | 5.45 | 0 | 10 | 10 | 0 | without Anion |
| no catalyst | 0 | 0 | 0 | 105 | 30 | 5.45 | 0 | 10 | 9.97 | 0.3 | without Anion |
| no catalyst | 0 | 0 | 0 | 105 | 60 | 5.45 | 0 | 10 | 9.95 | 0.5 | without Anion |
| no catalyst | 0 | 0 | 0 | 105 | 90 | 5.45 | 0 | 10 | 9.91 | 0.9 | without Anion |
| no catalyst | 0 | 0 | 0 | 105 | 120 | 5.45 | 0 | 10 | 9.88 | 1.2 | without Anion |
| no catalyst | 0 | 0 | 0 | 105 | 150 | 5.45 | 0 | 10 | 9.85 | 1.5 | without Anion |
| no catalyst | 0 | 0 | 0 | 105 | 180 | 5.45 | 0 | 10 | 9.81 | 1.9 | without Anion |
| no catalyst | 0 | 0 | 0 | 105 | 210 | 5.45 | 0 | 10 | 9.79 | 2.1 | without Anion |
| no catalyst | 0 | 0 | 0 | 105 | 240 | 5.45 | 0 | 10 | 9.79 | 2.1 | without Anion |
| no catalyst | 0 | 0 | 0 | 105 | 270 | 5.45 | 0 | 10 | 9.78 | 2.2 | without Anion |
| no catalyst | 0 | 0 | 0 | 105 | 0 | 5.45 | 0 | 10 | 10 | 0 | without Anion |
| no catalyst | 0 | 0 | 0 | 105 | 30 | 5.45 | 0 | 10 | 9.97 | 0.3 | without Anion |
| no catalyst | 0 | 0 | 0 | 105 | 60 | 5.45 | 0 | 10 | 9.91 | 0.9 | without Anion |
| no catalyst | 0 | 0 | 0 | 105 | 90 | 5.45 | 0 | 10 | 9.88 | 1.2 | without Anion |
| no catalyst | 0 | 0 | 0 | 105 | 120 | 5.45 | 0 | 10 | 9.87 | 1.3 | without Anion |
| no catalyst | 0 | 0 | 0 | 105 | 150 | 5.45 | 0 | 10 | 9.85 | 1.5 | without Anion |
| no catalyst | 0 | 0 | 0 | 105 | 180 | 5.45 | 0 | 10 | 9.83 | 1.7 | without Anion |
| no catalyst | 0 | 0 | 0 | 105 | 210 | 5.45 | 0 | 10 | 9.8 | 2 | without Anion |
| no catalyst | 0 | 0 | 0 | 105 | 240 | 5.45 | 0 | 10 | 9.79 | 2.1 | without Anion |
| no catalyst | 0 | 0 | 0 | 105 | 270 | 5.45 | 0 | 10 | 9.78 | 2.2 | without Anion |
| pure BFO | 11.2 | 0.0028 | 1 | 105 | 0 | 5.45 | 0 | 10 | 10 | 0 | without Anion |
| pure BFO | 11.2 | 0.0028 | 1 | 105 | 30 | 5.45 | 0 | 10 | 8.64 | 13.6 | without Anion |
| pure BFO | 11.2 | 0.0028 | 1 | 105 | 60 | 5.45 | 0 | 10 | 7.44 | 25.6 | without Anion |
| pure BFO | 11.2 | 0.0028 | 1 | 105 | 90 | 5.45 | 0 | 10 | 6.46 | 35.4 | without Anion |
| pure BFO | 11.2 | 0.0028 | 1 | 105 | 120 | 5.45 | 0 | 10 | 5.8 | 42 | without Anion |
| pure BFO | 11.2 | 0.0028 | 1 | 105 | 150 | 5.45 | 0 | 10 | 5.33 | 46.7 | without Anion |
| pure BFO | 11.2 | 0.0028 | 1 | 105 | 180 | 5.45 | 0 | 10 | 4.73 | 52.7 | without Anion |
| pure BFO | 11.2 | 0.0028 | 1 | 105 | 210 | 5.45 | 0 | 10 | 3.95 | 60.5 | without Anion |
| pure BFO | 11.2 | 0.0028 | 1 | 105 | 240 | 5.45 | 0 | 10 | 3.2 | 68 | without Anion |
| pure BFO | 11.2 | 0.0028 | 1 | 105 | 270 | 5.45 | 0 | 10 | 2.8 | 72 | without Anion |
| pure BFO | 11.2 | 0.0028 | 1 | 105 | 0 | 5.45 | 0 | 10 | 10 | 0 | without Anion |
| pure BFO | 11.2 | 0.0028 | 1 | 105 | 30 | 5.45 | 0 | 10 | 8.59 | 14.1 | without Anion |
| pure BFO | 11.2 | 0.0028 | 1 | 105 | 60 | 5.45 | 0 | 10 | 7.4 | 26 | without Anion |
| pure BFO | 11.2 | 0.0028 | 1 | 105 | 90 | 5.45 | 0 | 10 | 6.41 | 35.9 | without Anion |
| pure BFO | 11.2 | 0.0028 | 1 | 105 | 120 | 5.45 | 0 | 10 | 5.75 | 42.5 | without Anion |
| pure BFO | 11.2 | 0.0028 | 1 | 105 | 150 | 5.45 | 0 | 10 | 5.28 | 47.2 | without Anion |
| pure BFO | 11.2 | 0.0028 | 1 | 105 | 180 | 5.45 | 0 | 10 | 4.68 | 53.2 | without Anion |
| pure BFO | 11.2 | 0.0028 | 1 | 105 | 210 | 5.45 | 0 | 10 | 3.9 | 61 | without Anion |
| pure BFO | 11.2 | 0.0028 | 1 | 105 | 240 | 5.45 | 0 | 10 | 3.16 | 68.4 | without Anion |
| pure BFO | 11.2 | 0.0028 | 1 | 105 | 270 | 5.45 | 0 | 10 | 2.76 | 72.4 | without Anion |
| pure BFO | 11.2 | 0.0028 | 1 | 105 | 0 | 5.45 | 0 | 10 | 10 | 0 | without Anion |
| pure BFO | 11.2 | 0.0028 | 1 | 105 | 30 | 5.45 | 0 | 10 | 8.61 | 13.9 | without Anion |
| pure BFO | 11.2 | 0.0028 | 1 | 105 | 60 | 5.45 | 0 | 10 | 7.44 | 25.6 | without Anion |
| pure BFO | 11.2 | 0.0028 | 1 | 105 | 90 | 5.45 | 0 | 10 | 6.45 | 35.5 | without Anion |
| pure BFO | 11.2 | 0.0028 | 1 | 105 | 120 | 5.45 | 0 | 10 | 5.79 | 42.1 | without Anion |
| pure BFO | 11.2 | 0.0028 | 1 | 105 | 150 | 5.45 | 0 | 10 | 5.31 | 46.9 | without Anion |
| pure BFO | 11.2 | 0.0028 | 1 | 105 | 180 | 5.45 | 0 | 10 | 4.71 | 52.9 | without Anion |
| pure BFO | 11.2 | 0.0028 | 1 | 105 | 210 | 5.45 | 0 | 10 | 3.94 | 60.6 | without Anion |
| pure BFO | 11.2 | 0.0028 | 1 | 105 | 240 | 5.45 | 0 | 10 | 3.19 | 68.1 | without Anion |
| pure BFO | 11.2 | 0.0028 | 1 | 105 | 270 | 5.45 | 0 | 10 | 2.79 | 72.1 | without Anion |
| 0.5 wt% Pd-BFO | 13.4 | 0.0033 | 1 | 105 | 0 | 5.45 | 0 | 10 | 10 | 0 | without Anion |
| 0.5 wt% Pd-BFO | 13.4 | 0.0033 | 1 | 105 | 30 | 5.45 | 0 | 10 | 8.55 | 14.5 | without Anion |
| 0.5 wt% Pd-BFO | 13.4 | 0.0033 | 1 | 105 | 60 | 5.45 | 0 | 10 | 7.25 | 27.5 | without Anion |
| 0.5 wt% Pd-BFO | 13.4 | 0.0033 | 1 | 105 | 90 | 5.45 | 0 | 10 | 6.01 | 39.9 | without Anion |
| 0.5 wt% Pd-BFO | 13.4 | 0.0033 | 1 | 105 | 120 | 5.45 | 0 | 10 | 5.39 | 46.1 | without Anion |
| 0.5 wt% Pd-BFO | 13.4 | 0.0033 | 1 | 105 | 150 | 5.45 | 0 | 10 | 4.77 | 52.3 | without Anion |
| 0.5 wt% Pd-BFO | 13.4 | 0.0033 | 1 | 105 | 180 | 5.45 | 0 | 10 | 4.06 | 59.4 | without Anion |
| 0.5 wt% Pd-BFO | 13.4 | 0.0033 | 1 | 105 | 210 | 5.45 | 0 | 10 | 3.26 | 67.4 | without Anion |
| 0.5 wt% Pd-BFO | 13.4 | 0.0033 | 1 | 105 | 240 | 5.45 | 0 | 10 | 2.69 | 73.1 | without Anion |
| 0.5 wt% Pd-BFO | 13.4 | 0.0033 | 1 | 105 | 270 | 5.45 | 0 | 10 | 2.04 | 79.6 | without Anion |
| 0.5 wt% Pd-BFO | 13.4 | 0.0033 | 1 | 105 | 0 | 5.45 | 0 | 10 | 10 | 0 | without Anion |
| 0.5 wt% Pd-BFO | 13.4 | 0.0033 | 1 | 105 | 30 | 5.45 | 0 | 10 | 9.03 | 9.7 | without Anion |
| 0.5 wt% Pd-BFO | 13.4 | 0.0033 | 1 | 105 | 60 | 5.45 | 0 | 10 | 7.21 | 27.9 | without Anion |
| 0.5 wt% Pd-BFO | 13.4 | 0.0033 | 1 | 105 | 90 | 5.45 | 0 | 10 | 5.97 | 40.3 | without Anion |
| 0.5 wt% Pd-BFO | 13.4 | 0.0033 | 1 | 105 | 120 | 5.45 | 0 | 10 | 5.34 | 46.6 | without Anion |
| 0.5 wt% Pd-BFO | 13.4 | 0.0033 | 1 | 105 | 150 | 5.45 | 0 | 10 | 4.72 | 52.8 | without Anion |
| 0.5 wt% Pd-BFO | 13.4 | 0.0033 | 1 | 105 | 180 | 5.45 | 0 | 10 | 4.02 | 59.8 | without Anion |
| 0.5 wt% Pd-BFO | 13.4 | 0.0033 | 1 | 105 | 210 | 5.45 | 0 | 10 | 3.21 | 67.9 | without Anion |
| 0.5 wt% Pd-BFO | 13.4 | 0.0033 | 1 | 105 | 240 | 5.45 | 0 | 10 | 2.63 | 73.7 | without Anion |
| 0.5 wt% Pd-BFO | 13.4 | 0.0033 | 1 | 105 | 270 | 5.45 | 0 | 10 | 1.98 | 80.2 | without Anion |
| 0.5 wt% Pd-BFO | 13.4 | 0.0033 | 1 | 105 | 0 | 5.45 | 0 | 10 | 10 | 0 | without Anion |
| 0.5 wt% Pd-BFO | 13.4 | 0.0033 | 1 | 105 | 30 | 5.45 | 0 | 10 | 8.77 | 12.3 | without Anion |
| 0.5 wt% Pd-BFO | 13.4 | 0.0033 | 1 | 105 | 60 | 5.45 | 0 | 10 | 7.28 | 27.2 | without Anion |
| 0.5 wt% Pd-BFO | 13.4 | 0.0033 | 1 | 105 | 90 | 5.45 | 0 | 10 | 5.94 | 40.6 | without Anion |
| 0.5 wt% Pd-BFO | 13.4 | 0.0033 | 1 | 105 | 120 | 5.45 | 0 | 10 | 5.29 | 47.1 | without Anion |
| 0.5 wt% Pd-BFO | 13.4 | 0.0033 | 1 | 105 | 150 | 5.45 | 0 | 10 | 4.69 | 53.1 | without Anion |
| 0.5 wt% Pd-BFO | 13.4 | 0.0033 | 1 | 105 | 180 | 5.45 | 0 | 10 | 3.97 | 60.3 | without Anion |
| 0.5 wt% Pd-BFO | 13.4 | 0.0033 | 1 | 105 | 210 | 5.45 | 0 | 10 | 3.16 | 68.4 | without Anion |
| 0.5 wt% Pd-BFO | 13.4 | 0.0033 | 1 | 105 | 240 | 5.45 | 0 | 10 | 2.55 | 74.5 | without Anion |
| 0.5 wt% Pd-BFO | 13.4 | 0.0033 | 1 | 105 | 270 | 5.45 | 0 | 10 | 1.93 | 80.7 | without Anion |
| 1 wt% Pd-BFO | 16.7 | 0.0037 | 1 | 105 | 0 | 5.45 | 0 | 10 | 10 | 0 | without Anion |
| 1 wt% Pd-BFO | 16.7 | 0.0037 | 1 | 105 | 30 | 5.45 | 0 | 10 | 9.01 | 9.9 | without Anion |
| 1 wt% Pd-BFO | 16.7 | 0.0037 | 1 | 105 | 60 | 5.45 | 0 | 10 | 7.64 | 23.6 | without Anion |
| 1 wt% Pd-BFO | 16.7 | 0.0037 | 1 | 105 | 90 | 5.45 | 0 | 10 | 5.97 | 40.3 | without Anion |
| 1 wt% Pd-BFO | 16.7 | 0.0037 | 1 | 105 | 120 | 5.45 | 0 | 10 | 5.06 | 49.4 | without Anion |
| 1 wt% Pd-BFO | 16.7 | 0.0037 | 1 | 105 | 150 | 5.45 | 0 | 10 | 4.35 | 56.5 | without Anion |
| 1 wt% Pd-BFO | 16.7 | 0.0037 | 1 | 105 | 180 | 5.45 | 0 | 10 | 3.44 | 65.6 | without Anion |
| 1 wt% Pd-BFO | 16.7 | 0.0037 | 1 | 105 | 210 | 5.45 | 0 | 10 | 2.52 | 74.8 | without Anion |
| 1 wt% Pd-BFO | 16.7 | 0.0037 | 1 | 105 | 240 | 5.45 | 0 | 10 | 1.99 | 80.1 | without Anion |
| 1 wt% Pd-BFO | 16.7 | 0.0037 | 1 | 105 | 270 | 5.45 | 0 | 10 | 1.42 | 85.8 | without Anion |
| 1 wt% Pd-BFO | 16.7 | 0.0037 | 1 | 105 | 0 | 5.45 | 0 | 10 | 10 | 0 | without Anion |
| 1 wt% Pd-BFO | 16.7 | 0.0037 | 1 | 105 | 30 | 5.45 | 0 | 10 | 8.93 | 10.7 | without Anion |
| 1 wt% Pd-BFO | 16.7 | 0.0037 | 1 | 105 | 60 | 5.45 | 0 | 10 | 7.69 | 23.1 | without Anion |
| 1 wt% Pd-BFO | 16.7 | 0.0037 | 1 | 105 | 90 | 5.45 | 0 | 10 | 6.03 | 39.7 | without Anion |
| 1 wt% Pd-BFO | 16.7 | 0.0037 | 1 | 105 | 120 | 5.45 | 0 | 10 | 5.13 | 48.7 | without Anion |
| 1 wt% Pd-BFO | 16.7 | 0.0037 | 1 | 105 | 150 | 5.45 | 0 | 10 | 4.41 | 55.9 | without Anion |
| 1 wt% Pd-BFO | 16.7 | 0.0037 | 1 | 105 | 180 | 5.45 | 0 | 10 | 3.5 | 65 | without Anion |
| 1 wt% Pd-BFO | 16.7 | 0.0037 | 1 | 105 | 210 | 5.45 | 0 | 10 | 2.59 | 74.1 | without Anion |
| 1 wt% Pd-BFO | 16.7 | 0.0037 | 1 | 105 | 240 | 5.45 | 0 | 10 | 2.04 | 79.6 | without Anion |
| 1 wt% Pd-BFO | 16.7 | 0.0037 | 1 | 105 | 270 | 5.45 | 0 | 10 | 1.51 | 84.9 | without Anion |
| 1 wt% Pd-BFO | 16.7 | 0.0037 | 1 | 105 | 0 | 5.45 | 0 | 10 | 10 | 0 | without Anion |
| 1 wt% Pd-BFO | 16.7 | 0.0037 | 1 | 105 | 30 | 5.45 | 0 | 10 | 8.8 | 12 | without Anion |
| 1 wt% Pd-BFO | 16.7 | 0.0037 | 1 | 105 | 60 | 5.45 | 0 | 10 | 7.67 | 23.3 | without Anion |
| 1 wt% Pd-BFO | 16.7 | 0.0037 | 1 | 105 | 90 | 5.45 | 0 | 10 | 6 | 40 | without Anion |
| 1 wt% Pd-BFO | 16.7 | 0.0037 | 1 | 105 | 120 | 5.45 | 0 | 10 | 5.02 | 49.8 | without Anion |
| 1 wt% Pd-BFO | 16.7 | 0.0037 | 1 | 105 | 150 | 5.45 | 0 | 10 | 4.37 | 56.3 | without Anion |
| 1 wt% Pd-BFO | 16.7 | 0.0037 | 1 | 105 | 180 | 5.45 | 0 | 10 | 3.4 | 66 | without Anion |
| 1 wt% Pd-BFO | 16.7 | 0.0037 | 1 | 105 | 210 | 5.45 | 0 | 10 | 2.55 | 74.5 | without Anion |
| 1 wt% Pd-BFO | 16.7 | 0.0037 | 1 | 105 | 240 | 5.45 | 0 | 10 | 2.01 | 79.9 | without Anion |
| 1 wt% Pd-BFO | 16.7 | 0.0037 | 1 | 105 | 270 | 5.45 | 0 | 10 | 1.47 | 85.3 | without Anion |
| 2 wt% Pd-BFO | 22.9 | 0.0046 | 1 | 105 | 0 | 5.45 | 0 | 10 | 10 | 0 | without Anion |
| 2 wt% Pd-BFO | 22.9 | 0.0046 | 1 | 105 | 30 | 5.45 | 0 | 10 | 8.34 | 16.6 | without Anion |
| 2 wt% Pd-BFO | 22.9 | 0.0046 | 1 | 105 | 60 | 5.45 | 0 | 10 | 6.23 | 37.7 | without Anion |
| 2 wt% Pd-BFO | 22.9 | 0.0046 | 1 | 105 | 90 | 5.45 | 0 | 10 | 4.62 | 53.8 | without Anion |
| 2 wt% Pd-BFO | 22.9 | 0.0046 | 1 | 105 | 120 | 5.45 | 0 | 10 | 3.83 | 61.7 | without Anion |
| 2 wt% Pd-BFO | 22.9 | 0.0046 | 1 | 105 | 150 | 5.45 | 0 | 10 | 2.92 | 70.8 | without Anion |
| 2 wt% Pd-BFO | 22.9 | 0.0046 | 1 | 105 | 180 | 5.45 | 0 | 10 | 2.13 | 78.7 | without Anion |
| 2 wt% Pd-BFO | 22.9 | 0.0046 | 1 | 105 | 210 | 5.45 | 0 | 10 | 1.67 | 83.3 | without Anion |
| 2 wt% Pd-BFO | 22.9 | 0.0046 | 1 | 105 | 240 | 5.45 | 0 | 10 | 1.04 | 89.6 | without Anion |
| 2 wt% Pd-BFO | 22.9 | 0.0046 | 1 | 105 | 270 | 5.45 | 0 | 10 | 0.44 | 95.6 | without Anion |
| 2 wt% Pd-BFO | 22.9 | 0.0046 | 1 | 105 | 0 | 5.45 | 0 | 10 | 10 | 0 | without Anion |
| 2 wt% Pd-BFO | 22.9 | 0.0046 | 1 | 105 | 30 | 5.45 | 0 | 10 | 8.27 | 17.3 | without Anion |
| 2 wt% Pd-BFO | 22.9 | 0.0046 | 1 | 105 | 60 | 5.45 | 0 | 10 | 6.18 | 38.2 | without Anion |
| 2 wt% Pd-BFO | 22.9 | 0.0046 | 1 | 105 | 90 | 5.45 | 0 | 10 | 4.56 | 54.4 | without Anion |
| 2 wt% Pd-BFO | 22.9 | 0.0046 | 1 | 105 | 120 | 5.45 | 0 | 10 | 3.77 | 62.3 | without Anion |
| 2 wt% Pd-BFO | 22.9 | 0.0046 | 1 | 105 | 150 | 5.45 | 0 | 10 | 2.88 | 71.2 | without Anion |
| 2 wt% Pd-BFO | 22.9 | 0.0046 | 1 | 105 | 180 | 5.45 | 0 | 10 | 2.07 | 79.3 | without Anion |
| 2 wt% Pd-BFO | 22.9 | 0.0046 | 1 | 105 | 210 | 5.45 | 0 | 10 | 1.61 | 83.9 | without Anion |
| 2 wt% Pd-BFO | 22.9 | 0.0046 | 1 | 105 | 240 | 5.45 | 0 | 10 | 0.98 | 90.2 | without Anion |
| 2 wt% Pd-BFO | 22.9 | 0.0046 | 1 | 105 | 270 | 5.45 | 0 | 10 | 0.37 | 96.3 | without Anion |
| 2 wt% Pd-BFO | 22.9 | 0.0046 | 1 | 105 | 0 | 5.45 | 0 | 10 | 10 | 0 | without Anion |
| 2 wt% Pd-BFO | 22.9 | 0.0046 | 1 | 105 | 30 | 5.45 | 0 | 10 | 8.3 | 17 | without Anion |
| 2 wt% Pd-BFO | 22.9 | 0.0046 | 1 | 105 | 60 | 5.45 | 0 | 10 | 6.2 | 38 | without Anion |
| 2 wt% Pd-BFO | 22.9 | 0.0046 | 1 | 105 | 90 | 5.45 | 0 | 10 | 4.52 | 54.8 | without Anion |
| 2 wt% Pd-BFO | 22.9 | 0.0046 | 1 | 105 | 120 | 5.45 | 0 | 10 | 3.8 | 62 | without Anion |
| 2 wt% Pd-BFO | 22.9 | 0.0046 | 1 | 105 | 150 | 5.45 | 0 | 10 | 2.85 | 71.5 | without Anion |
| 2 wt% Pd-BFO | 22.9 | 0.0046 | 1 | 105 | 180 | 5.45 | 0 | 10 | 2.09 | 79.1 | without Anion |
| 2 wt% Pd-BFO | 22.9 | 0.0046 | 1 | 105 | 210 | 5.45 | 0 | 10 | 1.65 | 83.5 | without Anion |
| 2 wt% Pd-BFO | 22.9 | 0.0046 | 1 | 105 | 240 | 5.45 | 0 | 10 | 1.02 | 89.8 | without Anion |
| 2 wt% Pd-BFO | 22.9 | 0.0046 | 1 | 105 | 270 | 5.45 | 0 | 10 | 0.35 | 96.5 | without Anion |
| 3 wt% Pd-BFO | 19.5 | 0.0042 | 1 | 105 | 0 | 5.45 | 0 | 10 | 10 | 0 | without Anion |
| 3 wt% Pd-BFO | 19.5 | 0.0042 | 1 | 105 | 30 | 5.45 | 0 | 10 | 8.94 | 10.6 | without Anion |
| 3 wt% Pd-BFO | 19.5 | 0.0042 | 1 | 105 | 60 | 5.45 | 0 | 10 | 7.44 | 25.6 | without Anion |
| 3 wt% Pd-BFO | 19.5 | 0.0042 | 1 | 105 | 90 | 5.45 | 0 | 10 | 5.77 | 42.3 | without Anion |
| 3 wt% Pd-BFO | 19.5 | 0.0042 | 1 | 105 | 120 | 5.45 | 0 | 10 | 4.82 | 51.8 | without Anion |
| 3 wt% Pd-BFO | 19.5 | 0.0042 | 1 | 105 | 150 | 5.45 | 0 | 10 | 3.63 | 63.7 | without Anion |
| 3 wt% Pd-BFO | 19.5 | 0.0042 | 1 | 105 | 180 | 5.45 | 0 | 10 | 2.71 | 72.9 | without Anion |
| 3 wt% Pd-BFO | 19.5 | 0.0042 | 1 | 105 | 210 | 5.45 | 0 | 10 | 2.01 | 79.9 | without Anion |
| 3 wt% Pd-BFO | 19.5 | 0.0042 | 1 | 105 | 240 | 5.45 | 0 | 10 | 1.62 | 83.8 | without Anion |
| 3 wt% Pd-BFO | 19.5 | 0.0042 | 1 | 105 | 270 | 5.45 | 0 | 10 | 1.02 | 89.8 | without Anion |
| 3 wt% Pd-BFO | 19.5 | 0.0042 | 1 | 105 | 0 | 5.45 | 0 | 10 | 10 | 0 | without Anion |
| 3 wt% Pd-BFO | 19.5 | 0.0042 | 1 | 105 | 30 | 5.45 | 0 | 10 | 8.89 | 11.1 | without Anion |
| 3 wt% Pd-BFO | 19.5 | 0.0042 | 1 | 105 | 60 | 5.45 | 0 | 10 | 7.36 | 26.4 | without Anion |
| 3 wt% Pd-BFO | 19.5 | 0.0042 | 1 | 105 | 90 | 5.45 | 0 | 10 | 5.71 | 42.9 | without Anion |
| 3 wt% Pd-BFO | 19.5 | 0.0042 | 1 | 105 | 120 | 5.45 | 0 | 10 | 4.74 | 52.6 | without Anion |
| 3 wt% Pd-BFO | 19.5 | 0.0042 | 1 | 105 | 150 | 5.45 | 0 | 10 | 3.58 | 64.2 | without Anion |
| 3 wt% Pd-BFO | 19.5 | 0.0042 | 1 | 105 | 180 | 5.45 | 0 | 10 | 2.66 | 73.4 | without Anion |
| 3 wt% Pd-BFO | 19.5 | 0.0042 | 1 | 105 | 210 | 5.45 | 0 | 10 | 1.97 | 80.3 | without Anion |
| 3 wt% Pd-BFO | 19.5 | 0.0042 | 1 | 105 | 240 | 5.45 | 0 | 10 | 1.58 | 84.2 | without Anion |
| 3 wt% Pd-BFO | 19.5 | 0.0042 | 1 | 105 | 270 | 5.45 | 0 | 10 | 0.97 | 90.3 | without Anion |
| 3 wt% Pd-BFO | 19.5 | 0.0042 | 1 | 105 | 0 | 5.45 | 0 | 10 | 10 | 0 | without Anion |
| 3 wt% Pd-BFO | 19.5 | 0.0042 | 1 | 105 | 30 | 5.45 | 0 | 10 | 8.9 | 11 | without Anion |
| 3 wt% Pd-BFO | 19.5 | 0.0042 | 1 | 105 | 60 | 5.45 | 0 | 10 | 7.4 | 26 | without Anion |
| 3 wt% Pd-BFO | 19.5 | 0.0042 | 1 | 105 | 90 | 5.45 | 0 | 10 | 5.75 | 42.5 | without Anion |
| 3 wt% Pd-BFO | 19.5 | 0.0042 | 1 | 105 | 120 | 5.45 | 0 | 10 | 4.77 | 52.3 | without Anion |
| 3 wt% Pd-BFO | 19.5 | 0.0042 | 1 | 105 | 150 | 5.45 | 0 | 10 | 3.61 | 63.9 | without Anion |
| 3 wt% Pd-BFO | 19.5 | 0.0042 | 1 | 105 | 180 | 5.45 | 0 | 10 | 2.68 | 73.2 | without Anion |
| 3 wt% Pd-BFO | 19.5 | 0.0042 | 1 | 105 | 210 | 5.45 | 0 | 10 | 1.99 | 80.1 | without Anion |
| 3 wt% Pd-BFO | 19.5 | 0.0042 | 1 | 105 | 240 | 5.45 | 0 | 10 | 1.6 | 84 | without Anion |
| 3 wt% Pd-BFO | 19.5 | 0.0042 | 1 | 105 | 270 | 5.45 | 0 | 10 | 0.99 | 90.1 | without Anion |
| commercial TiO2 | 45 | 0.0051 | 1 | 105 | 0 | 5.45 | 0 | 10 | 10 | 0 | without Anion |
| commercial TiO2 | 45 | 0.0051 | 1 | 105 | 30 | 5.45 | 0 | 10 | 8.9 | 11 | without Anion |
| commercial TiO2 | 45 | 0.0051 | 1 | 105 | 60 | 5.45 | 0 | 10 | 7.54 | 24.6 | without Anion |
| commercial TiO2 | 45 | 0.0051 | 1 | 105 | 90 | 5.45 | 0 | 10 | 6.49 | 35.1 | without Anion |
| commercial TiO2 | 45 | 0.0051 | 1 | 105 | 120 | 5.45 | 0 | 10 | 5.51 | 44.9 | without Anion |
| commercial TiO2 | 45 | 0.0051 | 1 | 105 | 150 | 5.45 | 0 | 10 | 4.47 | 55.3 | without Anion |
| commercial TiO2 | 45 | 0.0051 | 1 | 105 | 180 | 5.45 | 0 | 10 | 3.69 | 63.1 | without Anion |
| commercial TiO2 | 45 | 0.0051 | 1 | 105 | 210 | 5.45 | 0 | 10 | 3.04 | 69.6 | without Anion |
| commercial TiO2 | 45 | 0.0051 | 1 | 105 | 240 | 5.45 | 0 | 10 | 2.21 | 77.9 | without Anion |
| commercial TiO2 | 45 | 0.0051 | 1 | 105 | 270 | 5.45 | 0 | 10 | 1.67 | 83.3 | without Anion |
| commercial TiO2 | 45 | 0.0051 | 1 | 105 | 0 | 5.45 | 0 | 10 | 10 | 0 | without Anion |
| commercial TiO2 | 45 | 0.0051 | 1 | 105 | 30 | 5.45 | 0 | 10 | 8.97 | 10.3 | without Anion |
| commercial TiO2 | 45 | 0.0051 | 1 | 105 | 60 | 5.45 | 0 | 10 | 7.62 | 23.8 | without Anion |
| commercial TiO2 | 45 | 0.0051 | 1 | 105 | 90 | 5.45 | 0 | 10 | 6.57 | 34.3 | without Anion |
| commercial TiO2 | 45 | 0.0051 | 1 | 105 | 120 | 5.45 | 0 | 10 | 5.58 | 44.2 | without Anion |
| commercial TiO2 | 45 | 0.0051 | 1 | 105 | 150 | 5.45 | 0 | 10 | 4.54 | 54.6 | without Anion |
| commercial TiO2 | 45 | 0.0051 | 1 | 105 | 180 | 5.45 | 0 | 10 | 3.75 | 62.5 | without Anion |
| commercial TiO2 | 45 | 0.0051 | 1 | 105 | 210 | 5.45 | 0 | 10 | 3.08 | 69.2 | without Anion |
| commercial TiO2 | 45 | 0.0051 | 1 | 105 | 240 | 5.45 | 0 | 10 | 2.28 | 77.2 | without Anion |
| commercial TiO2 | 45 | 0.0051 | 1 | 105 | 270 | 5.45 | 0 | 10 | 1.76 | 82.4 | without Anion |
| commercial TiO2 | 45 | 0.0051 | 1 | 105 | 0 | 5.45 | 0 | 10 | 10 | 0 | without Anion |
| commercial TiO2 | 45 | 0.0051 | 1 | 105 | 30 | 5.45 | 0 | 10 | 9 | 10 | without Anion |
| commercial TiO2 | 45 | 0.0051 | 1 | 105 | 60 | 5.45 | 0 | 10 | 7.64 | 23.6 | without Anion |
| commercial TiO2 | 45 | 0.0051 | 1 | 105 | 90 | 5.45 | 0 | 10 | 6.52 | 34.8 | without Anion |
| commercial TiO2 | 45 | 0.0051 | 1 | 105 | 120 | 5.45 | 0 | 10 | 5.54 | 44.6 | without Anion |
| commercial TiO2 | 45 | 0.0051 | 1 | 105 | 150 | 5.45 | 0 | 10 | 4.5 | 55 | without Anion |
| commercial TiO2 | 45 | 0.0051 | 1 | 105 | 180 | 5.45 | 0 | 10 | 3.74 | 62.6 | without Anion |
| commercial TiO2 | 45 | 0.0051 | 1 | 105 | 210 | 5.45 | 0 | 10 | 3.12 | 68.8 | without Anion |
| commercial TiO2 | 45 | 0.0051 | 1 | 105 | 240 | 5.45 | 0 | 10 | 2.24 | 77.6 | without Anion |
| commercial TiO2 | 45 | 0.0051 | 1 | 105 | 270 | 5.45 | 0 | 10 | 1.72 | 82.8 | without Anion |
| 1 wt% Ag-BFO | 11.9 | 0.0028 | 1 | 105 | 0 | 5.45 | 0 | 10 | 10 | 0 | without Anion |
| 1 wt% Ag-BFO | 11.9 | 0.0028 | 1 | 105 | 30 | 5.45 | 0 | 10 | 8.77 | 12.3 | without Anion |
| 1 wt% Ag-BFO | 11.9 | 0.0028 | 1 | 105 | 60 | 5.45 | 0 | 10 | 7.49 | 25.1 | without Anion |
| 1 wt% Ag-BFO | 11.9 | 0.0028 | 1 | 105 | 90 | 5.45 | 0 | 10 | 6.51 | 34.9 | without Anion |
| 1 wt% Ag-BFO | 11.9 | 0.0028 | 1 | 105 | 120 | 5.45 | 0 | 10 | 5.62 | 43.8 | without Anion |
| 1 wt% Ag-BFO | 11.9 | 0.0028 | 1 | 105 | 150 | 5.45 | 0 | 10 | 5.18 | 48.2 | without Anion |
| 1 wt% Ag-BFO | 11.9 | 0.0028 | 1 | 105 | 180 | 5.45 | 0 | 10 | 4.59 | 54.1 | without Anion |
| 1 wt% Ag-BFO | 11.9 | 0.0028 | 1 | 105 | 210 | 5.45 | 0 | 10 | 3.79 | 62.1 | without Anion |
| 1 wt% Ag-BFO | 11.9 | 0.0028 | 1 | 105 | 240 | 5.45 | 0 | 10 | 3.1 | 69 | without Anion |
| 1 wt% Ag-BFO | 11.9 | 0.0028 | 1 | 105 | 270 | 5.45 | 0 | 10 | 2.58 | 74.2 | without Anion |
| 1 wt% Ag-BFO | 11.9 | 0.0028 | 1 | 105 | 0 | 5.45 | 0 | 10 | 10 | 0 | without Anion |
| 1 wt% Ag-BFO | 11.9 | 0.0028 | 1 | 105 | 30 | 5.45 | 0 | 10 | 8.73 | 12.7 | without Anion |
| 1 wt% Ag-BFO | 11.9 | 0.0028 | 1 | 105 | 60 | 5.45 | 0 | 10 | 7.45 | 25.5 | without Anion |
| 1 wt% Ag-BFO | 11.9 | 0.0028 | 1 | 105 | 90 | 5.45 | 0 | 10 | 6.47 | 35.3 | without Anion |
| 1 wt% Ag-BFO | 11.9 | 0.0028 | 1 | 105 | 120 | 5.45 | 0 | 10 | 5.56 | 44.4 | without Anion |
| 1 wt% Ag-BFO | 11.9 | 0.0028 | 1 | 105 | 150 | 5.45 | 0 | 10 | 5.13 | 48.7 | without Anion |
| 1 wt% Ag-BFO | 11.9 | 0.0028 | 1 | 105 | 180 | 5.45 | 0 | 10 | 4.55 | 54.5 | without Anion |
| 1 wt% Ag-BFO | 11.9 | 0.0028 | 1 | 105 | 210 | 5.45 | 0 | 10 | 3.75 | 62.5 | without Anion |
| 1 wt% Ag-BFO | 11.9 | 0.0028 | 1 | 105 | 240 | 5.45 | 0 | 10 | 3.06 | 69.4 | without Anion |
| 1 wt% Ag-BFO | 11.9 | 0.0028 | 1 | 105 | 270 | 5.45 | 0 | 10 | 2.53 | 74.7 | without Anion |
| 1 wt% Ag-BFO | 11.9 | 0.0028 | 1 | 105 | 0 | 5.45 | 0 | 10 | 10 | 0 | without Anion |
| 1 wt% Ag-BFO | 11.9 | 0.0028 | 1 | 105 | 30 | 5.45 | 0 | 10 | 8.75 | 12.5 | without Anion |
| 1 wt% Ag-BFO | 11.9 | 0.0028 | 1 | 105 | 60 | 5.45 | 0 | 10 | 7.43 | 25.7 | without Anion |
| 1 wt% Ag-BFO | 11.9 | 0.0028 | 1 | 105 | 90 | 5.45 | 0 | 10 | 6.45 | 35.5 | without Anion |
| 1 wt% Ag-BFO | 11.9 | 0.0028 | 1 | 105 | 120 | 5.45 | 0 | 10 | 5.54 | 44.6 | without Anion |
| 1 wt% Ag-BFO | 11.9 | 0.0028 | 1 | 105 | 150 | 5.45 | 0 | 10 | 5.15 | 48.5 | without Anion |
| 1 wt% Ag-BFO | 11.9 | 0.0028 | 1 | 105 | 180 | 5.45 | 0 | 10 | 4.51 | 54.9 | without Anion |
| 1 wt% Ag-BFO | 11.9 | 0.0028 | 1 | 105 | 210 | 5.45 | 0 | 10 | 3.72 | 62.8 | without Anion |
| 1 wt% Ag-BFO | 11.9 | 0.0028 | 1 | 105 | 240 | 5.45 | 0 | 10 | 3.08 | 69.2 | without Anion |
| 1 wt% Ag-BFO | 11.9 | 0.0028 | 1 | 105 | 270 | 5.45 | 0 | 10 | 2.55 | 74.5 | without Anion |
| 2 wt% Ag-BFO | 14.6 | 0.0032 | 1 | 105 | 0 | 5.45 | 0 | 10 | 10 | 0 | without Anion |
| 2 wt% Ag-BFO | 14.6 | 0.0032 | 1 | 105 | 30 | 5.45 | 0 | 10 | 8.64 | 13.6 | without Anion |
| 2 wt% Ag-BFO | 14.6 | 0.0032 | 1 | 105 | 60 | 5.45 | 0 | 10 | 7.38 | 26.2 | without Anion |
| 2 wt% Ag-BFO | 14.6 | 0.0032 | 1 | 105 | 90 | 5.45 | 0 | 10 | 6.29 | 37.1 | without Anion |
| 2 wt% Ag-BFO | 14.6 | 0.0032 | 1 | 105 | 120 | 5.45 | 0 | 10 | 5.48 | 45.2 | without Anion |
| 2 wt% Ag-BFO | 14.6 | 0.0032 | 1 | 105 | 150 | 5.45 | 0 | 10 | 4.95 | 50.5 | without Anion |
| 2 wt% Ag-BFO | 14.6 | 0.0032 | 1 | 105 | 180 | 5.45 | 0 | 10 | 4.34 | 56.6 | without Anion |
| 2 wt% Ag-BFO | 14.6 | 0.0032 | 1 | 105 | 210 | 5.45 | 0 | 10 | 3.59 | 64.1 | without Anion |
| 2 wt% Ag-BFO | 14.6 | 0.0032 | 1 | 105 | 240 | 5.45 | 0 | 10 | 2.83 | 71.7 | without Anion |
| 2 wt% Ag-BFO | 14.6 | 0.0032 | 1 | 105 | 270 | 5.45 | 0 | 10 | 2.07 | 79.3 | without Anion |
| 2 wt% Ag-BFO | 14.6 | 0.0032 | 1 | 105 | 0 | 5.45 | 0 | 10 | 10 | 0 | without Anion |
| 2 wt% Ag-BFO | 14.6 | 0.0032 | 1 | 105 | 30 | 5.45 | 0 | 10 | 8.57 | 14.3 | without Anion |
| 2 wt% Ag-BFO | 14.6 | 0.0032 | 1 | 105 | 60 | 5.45 | 0 | 10 | 7.32 | 26.8 | without Anion |
| 2 wt% Ag-BFO | 14.6 | 0.0032 | 1 | 105 | 90 | 5.45 | 0 | 10 | 6.23 | 37.7 | without Anion |
| 2 wt% Ag-BFO | 14.6 | 0.0032 | 1 | 105 | 120 | 5.45 | 0 | 10 | 5.45 | 45.5 | without Anion |
| 2 wt% Ag-BFO | 14.6 | 0.0032 | 1 | 105 | 150 | 5.45 | 0 | 10 | 4.9 | 51 | without Anion |
| 2 wt% Ag-BFO | 14.6 | 0.0032 | 1 | 105 | 180 | 5.45 | 0 | 10 | 4.16 | 58.4 | without Anion |
| 2 wt% Ag-BFO | 14.6 | 0.0032 | 1 | 105 | 210 | 5.45 | 0 | 10 | 3.48 | 65.2 | without Anion |
| 2 wt% Ag-BFO | 14.6 | 0.0032 | 1 | 105 | 240 | 5.45 | 0 | 10 | 2.76 | 72.4 | without Anion |
| 2 wt% Ag-BFO | 14.6 | 0.0032 | 1 | 105 | 270 | 5.45 | 0 | 10 | 1.99 | 80.1 | without Anion |
| 2 wt% Ag-BFO | 14.6 | 0.0032 | 1 | 105 | 0 | 5.45 | 0 | 10 | 10 | 0 | without Anion |
| 2 wt% Ag-BFO | 14.6 | 0.0032 | 1 | 105 | 30 | 5.45 | 0 | 10 | 8.69 | 13.1 | without Anion |
| 2 wt% Ag-BFO | 14.6 | 0.0032 | 1 | 105 | 60 | 5.45 | 0 | 10 | 7.35 | 26.5 | without Anion |
| 2 wt% Ag-BFO | 14.6 | 0.0032 | 1 | 105 | 90 | 5.45 | 0 | 10 | 6.33 | 36.7 | without Anion |
| 2 wt% Ag-BFO | 14.6 | 0.0032 | 1 | 105 | 120 | 5.45 | 0 | 10 | 5.52 | 44.8 | without Anion |
| 2 wt% Ag-BFO | 14.6 | 0.0032 | 1 | 105 | 150 | 5.45 | 0 | 10 | 5.08 | 49.2 | without Anion |
| 2 wt% Ag-BFO | 14.6 | 0.0032 | 1 | 105 | 180 | 5.45 | 0 | 10 | 4.39 | 56.1 | without Anion |
| 2 wt% Ag-BFO | 14.6 | 0.0032 | 1 | 105 | 210 | 5.45 | 0 | 10 | 3.63 | 63.7 | without Anion |
| 2 wt% Ag-BFO | 14.6 | 0.0032 | 1 | 105 | 240 | 5.45 | 0 | 10 | 2.88 | 71.2 | without Anion |
| 2 wt% Ag-BFO | 14.6 | 0.0032 | 1 | 105 | 270 | 5.45 | 0 | 10 | 2.12 | 78.8 | without Anion |
| 3 wt% Ag-BFO | 17.8 | 0.0039 | 1 | 105 | 0 | 5.45 | 0 | 10 | 10 | 0 | without Anion |
| 3 wt% Ag-BFO | 17.8 | 0.0039 | 1 | 105 | 30 | 5.45 | 0 | 10 | 8.61 | 13.9 | without Anion |
| 3 wt% Ag-BFO | 17.8 | 0.0039 | 1 | 105 | 60 | 5.45 | 0 | 10 | 7.19 | 28.1 | without Anion |
| 3 wt% Ag-BFO | 17.8 | 0.0039 | 1 | 105 | 90 | 5.45 | 0 | 10 | 6.22 | 37.8 | without Anion |
| 3 wt% Ag-BFO | 17.8 | 0.0039 | 1 | 105 | 120 | 5.45 | 0 | 10 | 5.4 | 46 | without Anion |
| 3 wt% Ag-BFO | 17.8 | 0.0039 | 1 | 105 | 150 | 5.45 | 0 | 10 | 4.61 | 53.9 | without Anion |
| 3 wt% Ag-BFO | 17.8 | 0.0039 | 1 | 105 | 180 | 5.45 | 0 | 10 | 3.53 | 64.7 | without Anion |
| 3 wt% Ag-BFO | 17.8 | 0.0039 | 1 | 105 | 210 | 5.45 | 0 | 10 | 2.7 | 73 | without Anion |
| 3 wt% Ag-BFO | 17.8 | 0.0039 | 1 | 105 | 240 | 5.45 | 0 | 10 | 2.01 | 79.9 | without Anion |
| 3 wt% Ag-BFO | 17.8 | 0.0039 | 1 | 105 | 270 | 5.45 | 0 | 10 | 1.38 | 86.2 | without Anion |
| 3 wt% Ag-BFO | 17.8 | 0.0039 | 1 | 105 | 0 | 5.45 | 0 | 10 | 10 | 0 | without Anion |
| 3 wt% Ag-BFO | 17.8 | 0.0039 | 1 | 105 | 30 | 5.45 | 0 | 10 | 8.57 | 14.3 | without Anion |
| 3 wt% Ag-BFO | 17.8 | 0.0039 | 1 | 105 | 60 | 5.45 | 0 | 10 | 7.15 | 28.5 | without Anion |
| 3 wt% Ag-BFO | 17.8 | 0.0039 | 1 | 105 | 90 | 5.45 | 0 | 10 | 6.17 | 38.3 | without Anion |
| 3 wt% Ag-BFO | 17.8 | 0.0039 | 1 | 105 | 120 | 5.45 | 0 | 10 | 5.33 | 46.7 | without Anion |
| 3 wt% Ag-BFO | 17.8 | 0.0039 | 1 | 105 | 150 | 5.45 | 0 | 10 | 4.69 | 53.1 | without Anion |
| 3 wt% Ag-BFO | 17.8 | 0.0039 | 1 | 105 | 180 | 5.45 | 0 | 10 | 3.59 | 64.1 | without Anion |
| 3 wt% Ag-BFO | 17.8 | 0.0039 | 1 | 105 | 210 | 5.45 | 0 | 10 | 2.75 | 72.5 | without Anion |
| 3 wt% Ag-BFO | 17.8 | 0.0039 | 1 | 105 | 240 | 5.45 | 0 | 10 | 2.09 | 79.1 | without Anion |
| 3 wt% Ag-BFO | 17.8 | 0.0039 | 1 | 105 | 270 | 5.45 | 0 | 10 | 1.45 | 85.5 | without Anion |
| 3 wt% Ag-BFO | 17.8 | 0.0039 | 1 | 105 | 0 | 5.45 | 0 | 10 | 10 | 0 | without Anion |
| 3 wt% Ag-BFO | 17.8 | 0.0039 | 1 | 105 | 30 | 5.45 | 0 | 10 | 8.6 | 14 | without Anion |
| 3 wt% Ag-BFO | 17.8 | 0.0039 | 1 | 105 | 60 | 5.45 | 0 | 10 | 7.17 | 28.3 | without Anion |
| 3 wt% Ag-BFO | 17.8 | 0.0039 | 1 | 105 | 90 | 5.45 | 0 | 10 | 6.2 | 38 | without Anion |
| 3 wt% Ag-BFO | 17.8 | 0.0039 | 1 | 105 | 120 | 5.45 | 0 | 10 | 5.38 | 46.2 | without Anion |
| 3 wt% Ag-BFO | 17.8 | 0.0039 | 1 | 105 | 150 | 5.45 | 0 | 10 | 6.5 | 35 | without Anion |
| 3 wt% Ag-BFO | 17.8 | 0.0039 | 1 | 105 | 180 | 5.45 | 0 | 10 | 3.57 | 64.3 | without Anion |
| 3 wt% Ag-BFO | 17.8 | 0.0039 | 1 | 105 | 210 | 5.45 | 0 | 10 | 2.73 | 72.7 | without Anion |
| 3 wt% Ag-BFO | 17.8 | 0.0039 | 1 | 105 | 240 | 5.45 | 0 | 10 | 2.04 | 79.6 | without Anion |
| 3 wt% Ag-BFO | 17.8 | 0.0039 | 1 | 105 | 270 | 5.45 | 0 | 10 | 1.42 | 85.8 | without Anion |
| 4 wt% Ag-BFO | 15.1 | 0.0033 | 1 | 105 | 0 | 5.45 | 0 | 10 | 10 | 0 | without Anion |
| 4 wt% Ag-BFO | 15.1 | 0.0033 | 1 | 105 | 30 | 5.45 | 0 | 10 | 8.67 | 13.3 | without Anion |
| 4 wt% Ag-BFO | 15.1 | 0.0033 | 1 | 105 | 60 | 5.45 | 0 | 10 | 8.3 | 17 | without Anion |
| 4 wt% Ag-BFO | 15.1 | 0.0033 | 1 | 105 | 90 | 5.45 | 0 | 10 | 7.77 | 22.3 | without Anion |
| 4 wt% Ag-BFO | 15.1 | 0.0033 | 1 | 105 | 120 | 5.45 | 0 | 10 | 7.08 | 29.2 | without Anion |
| 4 wt% Ag-BFO | 15.1 | 0.0033 | 1 | 105 | 150 | 5.45 | 0 | 10 | 6.16 | 38.4 | without Anion |
| 4 wt% Ag-BFO | 15.1 | 0.0033 | 1 | 105 | 180 | 5.45 | 0 | 10 | 5.23 | 47.7 | without Anion |
| 4 wt% Ag-BFO | 15.1 | 0.0033 | 1 | 105 | 210 | 5.45 | 0 | 10 | 4.33 | 56.7 | without Anion |
| 4 wt% Ag-BFO | 15.1 | 0.0033 | 1 | 105 | 240 | 5.45 | 0 | 10 | 3.53 | 64.7 | without Anion |
| 4 wt% Ag-BFO | 15.1 | 0.0033 | 1 | 105 | 270 | 5.45 | 0 | 10 | 2.71 | 72.9 | without Anion |
| 4 wt% Ag-BFO | 15.1 | 0.0033 | 1 | 105 | 0 | 5.45 | 0 | 10 | 10 | 0 | without Anion |
| 4 wt% Ag-BFO | 15.1 | 0.0033 | 1 | 105 | 30 | 5.45 | 0 | 10 | 8.72 | 12.8 | without Anion |
| 4 wt% Ag-BFO | 15.1 | 0.0033 | 1 | 105 | 60 | 5.45 | 0 | 10 | 8.34 | 16.6 | without Anion |
| 4 wt% Ag-BFO | 15.1 | 0.0033 | 1 | 105 | 90 | 5.45 | 0 | 10 | 7.81 | 21.9 | without Anion |
| 4 wt% Ag-BFO | 15.1 | 0.0033 | 1 | 105 | 120 | 5.45 | 0 | 10 | 7.13 | 28.7 | without Anion |
| 4 wt% Ag-BFO | 15.1 | 0.0033 | 1 | 105 | 150 | 5.45 | 0 | 10 | 6.21 | 37.9 | without Anion |
| 4 wt% Ag-BFO | 15.1 | 0.0033 | 1 | 105 | 180 | 5.45 | 0 | 10 | 5.29 | 47.1 | without Anion |
| 4 wt% Ag-BFO | 15.1 | 0.0033 | 1 | 105 | 210 | 5.45 | 0 | 10 | 4.4 | 56 | without Anion |
| 4 wt% Ag-BFO | 15.1 | 0.0033 | 1 | 105 | 240 | 5.45 | 0 | 10 | 3.58 | 64.2 | without Anion |
| 4 wt% Ag-BFO | 15.1 | 0.0033 | 1 | 105 | 270 | 5.45 | 0 | 10 | 2.76 | 72.4 | without Anion |
| 4 wt% Ag-BFO | 15.1 | 0.0033 | 1 | 105 | 0 | 5.45 | 0 | 10 | 10 | 0 | without Anion |
| 4 wt% Ag-BFO | 15.1 | 0.0033 | 1 | 105 | 30 | 5.45 | 0 | 10 | 8.76 | 12.4 | without Anion |
| 4 wt% Ag-BFO | 15.1 | 0.0033 | 1 | 105 | 60 | 5.45 | 0 | 10 | 8.39 | 16.1 | without Anion |
| 4 wt% Ag-BFO | 15.1 | 0.0033 | 1 | 105 | 90 | 5.45 | 0 | 10 | 7.85 | 21.5 | without Anion |
| 4 wt% Ag-BFO | 15.1 | 0.0033 | 1 | 105 | 120 | 5.45 | 0 | 10 | 7.18 | 28.2 | without Anion |
| 4 wt% Ag-BFO | 15.1 | 0.0033 | 1 | 105 | 150 | 5.45 | 0 | 10 | 6.26 | 37.4 | without Anion |
| 4 wt% Ag-BFO | 15.1 | 0.0033 | 1 | 105 | 180 | 5.45 | 0 | 10 | 5.36 | 46.4 | without Anion |
| 4 wt% Ag-BFO | 15.1 | 0.0033 | 1 | 105 | 210 | 5.45 | 0 | 10 | 4.28 | 57.2 | without Anion |
| 4 wt% Ag-BFO | 15.1 | 0.0033 | 1 | 105 | 240 | 5.45 | 0 | 10 | 3.48 | 65.2 | without Anion |
| 4 wt% Ag-BFO | 15.1 | 0.0033 | 1 | 105 | 270 | 5.45 | 0 | 10 | 2.66 | 73.4 | without Anion |
| 0.25 wt% Pt-BFO | 13.5 | 0.003 | 1 | 105 | 0 | 5.45 | 0 | 10 | 10 | 0 | without Anion |
| 0.25 wt% Pt-BFO | 13.5 | 0.003 | 1 | 105 | 30 | 5.45 | 0 | 10 | 8.79 | 12.1 | without Anion |
| 0.25 wt% Pt-BFO | 13.5 | 0.003 | 1 | 105 | 60 | 5.45 | 0 | 10 | 7.82 | 21.8 | without Anion |
| 0.25 wt% Pt-BFO | 13.5 | 0.003 | 1 | 105 | 90 | 5.45 | 0 | 10 | 6.75 | 32.5 | without Anion |
| 0.25 wt% Pt-BFO | 13.5 | 0.003 | 1 | 105 | 120 | 5.45 | 0 | 10 | 5.88 | 41.2 | without Anion |
| 0.25 wt% Pt-BFO | 13.5 | 0.003 | 1 | 105 | 150 | 5.45 | 0 | 10 | 4.93 | 50.7 | without Anion |
| 0.25 wt% Pt-BFO | 13.5 | 0.003 | 1 | 105 | 180 | 5.45 | 0 | 10 | 4.01 | 59.9 | without Anion |
| 0.25 wt% Pt-BFO | 13.5 | 0.003 | 1 | 105 | 210 | 5.45 | 0 | 10 | 3.11 | 68.9 | without Anion |
| 0.25 wt% Pt-BFO | 13.5 | 0.003 | 1 | 105 | 240 | 5.45 | 0 | 10 | 2.23 | 77.7 | without Anion |
| 0.25 wt% Pt-BFO | 13.5 | 0.003 | 1 | 105 | 270 | 5.45 | 0 | 10 | 1.32 | 86.8 | without Anion |
| 0.25 wt% Pt-BFO | 13.5 | 0.003 | 1 | 105 | 0 | 5.45 | 0 | 10 | 10 | 0 | without Anion |
| 0.25 wt% Pt-BFO | 13.5 | 0.003 | 1 | 105 | 30 | 5.45 | 0 | 10 | 8.72 | 12.8 | without Anion |
| 0.25 wt% Pt-BFO | 13.5 | 0.003 | 1 | 105 | 60 | 5.45 | 0 | 10 | 7.74 | 22.6 | without Anion |
| 0.25 wt% Pt-BFO | 13.5 | 0.003 | 1 | 105 | 90 | 5.45 | 0 | 10 | 6.72 | 32.8 | without Anion |
| 0.25 wt% Pt-BFO | 13.5 | 0.003 | 1 | 105 | 120 | 5.45 | 0 | 10 | 5.91 | 40.9 | without Anion |
| 0.25 wt% Pt-BFO | 13.5 | 0.003 | 1 | 105 | 150 | 5.45 | 0 | 10 | 4.97 | 50.3 | without Anion |
| 0.25 wt% Pt-BFO | 13.5 | 0.003 | 1 | 105 | 180 | 5.45 | 0 | 10 | 4.08 | 59.2 | without Anion |
| 0.25 wt% Pt-BFO | 13.5 | 0.003 | 1 | 105 | 210 | 5.45 | 0 | 10 | 3.17 | 68.3 | without Anion |
| 0.25 wt% Pt-BFO | 13.5 | 0.003 | 1 | 105 | 240 | 5.45 | 0 | 10 | 2.29 | 77.1 | without Anion |
| 0.25 wt% Pt-BFO | 13.5 | 0.003 | 1 | 105 | 270 | 5.45 | 0 | 10 | 1.36 | 86.4 | without Anion |
| 0.25 wt% Pt-BFO | 13.5 | 0.003 | 1 | 105 | 0 | 5.45 | 0 | 10 | 10 | 0 | without Anion |
| 0.25 wt% Pt-BFO | 13.5 | 0.003 | 1 | 105 | 30 | 5.45 | 0 | 10 | 8.75 | 12.5 | without Anion |
| 0.25 wt% Pt-BFO | 13.5 | 0.003 | 1 | 105 | 60 | 5.45 | 0 | 10 | 7.76 | 22.4 | without Anion |
| 0.25 wt% Pt-BFO | 13.5 | 0.003 | 1 | 105 | 90 | 5.45 | 0 | 10 | 6.7 | 33 | without Anion |
| 0.25 wt% Pt-BFO | 13.5 | 0.003 | 1 | 105 | 120 | 5.45 | 0 | 10 | 5.84 | 41.6 | without Anion |
| 0.25 wt% Pt-BFO | 13.5 | 0.003 | 1 | 105 | 150 | 5.45 | 0 | 10 | 4.9 | 51 | without Anion |
| 0.25 wt% Pt-BFO | 13.5 | 0.003 | 1 | 105 | 180 | 5.45 | 0 | 10 | 4.04 | 59.6 | without Anion |
| 0.25 wt% Pt-BFO | 13.5 | 0.003 | 1 | 105 | 210 | 5.45 | 0 | 10 | 3.07 | 69.3 | without Anion |
| 0.25 wt% Pt-BFO | 13.5 | 0.003 | 1 | 105 | 240 | 5.45 | 0 | 10 | 2.2 | 78 | without Anion |
| 0.25 wt% Pt-BFO | 13.5 | 0.003 | 1 | 105 | 270 | 5.45 | 0 | 10 | 1.28 | 87.2 | without Anion |
| 0.5 wt% Pt-BFO | 19.5 | 0.0039 | 1 | 105 | 0 | 5.45 | 0 | 10 | 10 | 0 | without Anion |
| 0.5 wt% Pt-BFO | 19.5 | 0.0039 | 1 | 105 | 30 | 5.45 | 0 | 10 | 8.87 | 11.3 | without Anion |
| 0.5 wt% Pt-BFO | 19.5 | 0.0039 | 1 | 105 | 60 | 5.45 | 0 | 10 | 7.32 | 26.8 | without Anion |
| 0.5 wt% Pt-BFO | 19.5 | 0.0039 | 1 | 105 | 90 | 5.45 | 0 | 10 | 5.88 | 41.2 | without Anion |
| 0.5 wt% Pt-BFO | 19.5 | 0.0039 | 1 | 105 | 120 | 5.45 | 0 | 10 | 4.67 | 53.3 | without Anion |
| 0.5 wt% Pt-BFO | 19.5 | 0.0039 | 1 | 105 | 150 | 5.45 | 0 | 10 | 3.55 | 64.5 | without Anion |
| 0.5 wt% Pt-BFO | 19.5 | 0.0039 | 1 | 105 | 180 | 5.45 | 0 | 10 | 2.68 | 73.2 | without Anion |
| 0.5 wt% Pt-BFO | 19.5 | 0.0039 | 1 | 105 | 210 | 5.45 | 0 | 10 | 1.82 | 81.8 | without Anion |
| 0.5 wt% Pt-BFO | 19.5 | 0.0039 | 1 | 105 | 240 | 5.45 | 0 | 10 | 1.11 | 88.9 | without Anion |
| 0.5 wt% Pt-BFO | 19.5 | 0.0039 | 1 | 105 | 270 | 5.45 | 0 | 10 | 0.55 | 94.5 | without Anion |
| 0.5 wt% Pt-BFO | 19.5 | 0.0039 | 1 | 105 | 0 | 5.45 | 0 | 10 | 10 | 0 | without Anion |
| 0.5 wt% Pt-BFO | 19.5 | 0.0039 | 1 | 105 | 30 | 5.45 | 0 | 10 | 8.81 | 11.9 | without Anion |
| 0.5 wt% Pt-BFO | 19.5 | 0.0039 | 1 | 105 | 60 | 5.45 | 0 | 10 | 7.36 | 26.4 | without Anion |
| 0.5 wt% Pt-BFO | 19.5 | 0.0039 | 1 | 105 | 90 | 5.45 | 0 | 10 | 5.92 | 40.8 | without Anion |
| 0.5 wt% Pt-BFO | 19.5 | 0.0039 | 1 | 105 | 120 | 5.45 | 0 | 10 | 4.71 | 52.9 | without Anion |
| 0.5 wt% Pt-BFO | 19.5 | 0.0039 | 1 | 105 | 150 | 5.45 | 0 | 10 | 3.6 | 64 | without Anion |
| 0.5 wt% Pt-BFO | 19.5 | 0.0039 | 1 | 105 | 180 | 5.45 | 0 | 10 | 2.7 | 73 | without Anion |
| 0.5 wt% Pt-BFO | 19.5 | 0.0039 | 1 | 105 | 210 | 5.45 | 0 | 10 | 1.85 | 81.5 | without Anion |
| 0.5 wt% Pt-BFO | 19.5 | 0.0039 | 1 | 105 | 240 | 5.45 | 0 | 10 | 1.15 | 88.5 | without Anion |
| 0.5 wt% Pt-BFO | 19.5 | 0.0039 | 1 | 105 | 270 | 5.45 | 0 | 10 | 0.62 | 93.8 | without Anion |
| 0.5 wt% Pt-BFO | 19.5 | 0.0039 | 1 | 105 | 0 | 5.45 | 0 | 10 | 10 | 0 | without Anion |
| 0.5 wt% Pt-BFO | 19.5 | 0.0039 | 1 | 105 | 30 | 5.45 | 0 | 10 | 8.79 | 12.1 | without Anion |
| 0.5 wt% Pt-BFO | 19.5 | 0.0039 | 1 | 105 | 60 | 5.45 | 0 | 10 | 7.28 | 27.2 | without Anion |
| 0.5 wt% Pt-BFO | 19.5 | 0.0039 | 1 | 105 | 90 | 5.45 | 0 | 10 | 5.82 | 41.8 | without Anion |
| 0.5 wt% Pt-BFO | 19.5 | 0.0039 | 1 | 105 | 120 | 5.45 | 0 | 10 | 4.6 | 54 | without Anion |
| 0.5 wt% Pt-BFO | 19.5 | 0.0039 | 1 | 105 | 150 | 5.45 | 0 | 10 | 3.5 | 65 | without Anion |
| 0.5 wt% Pt-BFO | 19.5 | 0.0039 | 1 | 105 | 180 | 5.45 | 0 | 10 | 2.62 | 73.8 | without Anion |
| 0.5 wt% Pt-BFO | 19.5 | 0.0039 | 1 | 105 | 210 | 5.45 | 0 | 10 | 1.79 | 82.1 | without Anion |
| 0.5 wt% Pt-BFO | 19.5 | 0.0039 | 1 | 105 | 240 | 5.45 | 0 | 10 | 1.04 | 89.6 | without Anion |
| 0.5 wt% Pt-BFO | 19.5 | 0.0039 | 1 | 105 | 270 | 5.45 | 0 | 10 | 0.66 | 93.4 | without Anion |
| 1 wt% Pt-BFO | 17.8 | 0.0035 | 1 | 105 | 0 | 5.45 | 0 | 10 | 10 | 0 | without Anion |
| 1 wt% Pt-BFO | 17.8 | 0.0035 | 1 | 105 | 30 | 5.45 | 0 | 10 | 8.94 | 10.6 | without Anion |
| 1 wt% Pt-BFO | 17.8 | 0.0035 | 1 | 105 | 60 | 5.45 | 0 | 10 | 7.49 | 25.1 | without Anion |
| 1 wt% Pt-BFO | 17.8 | 0.0035 | 1 | 105 | 90 | 5.45 | 0 | 10 | 6.52 | 34.8 | without Anion |
| 1 wt% Pt-BFO | 17.8 | 0.0035 | 1 | 105 | 120 | 5.45 | 0 | 10 | 5.34 | 46.6 | without Anion |
| 1 wt% Pt-BFO | 17.8 | 0.0035 | 1 | 105 | 150 | 5.45 | 0 | 10 | 4.11 | 58.9 | without Anion |
| 1 wt% Pt-BFO | 17.8 | 0.0035 | 1 | 105 | 180 | 5.45 | 0 | 10 | 3.21 | 67.9 | without Anion |
| 1 wt% Pt-BFO | 17.8 | 0.0035 | 1 | 105 | 210 | 5.45 | 0 | 10 | 2.28 | 77.2 | without Anion |
| 1 wt% Pt-BFO | 17.8 | 0.0035 | 1 | 105 | 240 | 5.45 | 0 | 10 | 1.37 | 86.3 | without Anion |
| 1 wt% Pt-BFO | 17.8 | 0.0035 | 1 | 105 | 270 | 5.45 | 0 | 10 | 0.6 | 94 | without Anion |
| 1 wt% Pt-BFO | 17.8 | 0.0035 | 1 | 105 | 0 | 5.45 | 0 | 10 | 10 | 0 | without Anion |
| 1 wt% Pt-BFO | 17.8 | 0.0035 | 1 | 105 | 30 | 5.45 | 0 | 10 | 8.88 | 11.2 | without Anion |
| 1 wt% Pt-BFO | 17.8 | 0.0035 | 1 | 105 | 60 | 5.45 | 0 | 10 | 7.55 | 24.5 | without Anion |
| 1 wt% Pt-BFO | 17.8 | 0.0035 | 1 | 105 | 90 | 5.45 | 0 | 10 | 6.57 | 34.3 | without Anion |
| 1 wt% Pt-BFO | 17.8 | 0.0035 | 1 | 105 | 120 | 5.45 | 0 | 10 | 5.31 | 46.9 | without Anion |
| 1 wt% Pt-BFO | 17.8 | 0.0035 | 1 | 105 | 150 | 5.45 | 0 | 10 | 4.08 | 59.2 | without Anion |
| 1 wt% Pt-BFO | 17.8 | 0.0035 | 1 | 105 | 180 | 5.45 | 0 | 10 | 3.15 | 68.5 | without Anion |
| 1 wt% Pt-BFO | 17.8 | 0.0035 | 1 | 105 | 210 | 5.45 | 0 | 10 | 2.26 | 77.4 | without Anion |
| 1 wt% Pt-BFO | 17.8 | 0.0035 | 1 | 105 | 240 | 5.45 | 0 | 10 | 1.34 | 86.6 | without Anion |
| 1 wt% Pt-BFO | 17.8 | 0.0035 | 1 | 105 | 270 | 5.45 | 0 | 10 | 0.65 | 93.5 | without Anion |
| 1 wt% Pt-BFO | 17.8 | 0.0035 | 1 | 105 | 0 | 5.45 | 0 | 10 | 10 | 0 | without Anion |
| 1 wt% Pt-BFO | 17.8 | 0.0035 | 1 | 105 | 30 | 5.45 | 0 | 10 | 8.9 | 11 | without Anion |
| 1 wt% Pt-BFO | 17.8 | 0.0035 | 1 | 105 | 60 | 5.45 | 0 | 10 | 7.6 | 24 | without Anion |
| 1 wt% Pt-BFO | 17.8 | 0.0035 | 1 | 105 | 90 | 5.45 | 0 | 10 | 6.55 | 34.5 | without Anion |
| 1 wt% Pt-BFO | 17.8 | 0.0035 | 1 | 105 | 120 | 5.45 | 0 | 10 | 5.28 | 47.2 | without Anion |
| 1 wt% Pt-BFO | 17.8 | 0.0035 | 1 | 105 | 150 | 5.45 | 0 | 10 | 4.06 | 59.4 | without Anion |
| 1 wt% Pt-BFO | 17.8 | 0.0035 | 1 | 105 | 180 | 5.45 | 0 | 10 | 3.17 | 68.3 | without Anion |
| 1 wt% Pt-BFO | 17.8 | 0.0035 | 1 | 105 | 210 | 5.45 | 0 | 10 | 2.38 | 76.2 | without Anion |
| 1 wt% Pt-BFO | 17.8 | 0.0035 | 1 | 105 | 240 | 5.45 | 0 | 10 | 1.44 | 85.6 | without Anion |
| 1 wt% Pt-BFO | 17.8 | 0.0035 | 1 | 105 | 270 | 5.45 | 0 | 10 | 0.68 | 93.2 | without Anion |
| 2 wt% Pt-BFO | 16.4 | 0.0034 | 1 | 105 | 0 | 5.45 | 0 | 10 | 10 | 0 | without Anion |
| 2 wt% Pt-BFO | 16.4 | 0.0034 | 1 | 105 | 30 | 5.45 | 0 | 10 | 8.57 | 14.3 | without Anion |
| 2 wt% Pt-BFO | 16.4 | 0.0034 | 1 | 105 | 60 | 5.45 | 0 | 10 | 7.62 | 23.8 | without Anion |
| 2 wt% Pt-BFO | 16.4 | 0.0034 | 1 | 105 | 90 | 5.45 | 0 | 10 | 6.71 | 32.9 | without Anion |
| 2 wt% Pt-BFO | 16.4 | 0.0034 | 1 | 105 | 120 | 5.45 | 0 | 10 | 5.52 | 44.8 | without Anion |
| 2 wt% Pt-BFO | 16.4 | 0.0034 | 1 | 105 | 150 | 5.45 | 0 | 10 | 4.37 | 56.3 | without Anion |
| 2 wt% Pt-BFO | 16.4 | 0.0034 | 1 | 105 | 180 | 5.45 | 0 | 10 | 3.44 | 65.6 | without Anion |
| 2 wt% Pt-BFO | 16.4 | 0.0034 | 1 | 105 | 210 | 5.45 | 0 | 10 | 2.49 | 75.1 | without Anion |
| 2 wt% Pt-BFO | 16.4 | 0.0034 | 1 | 105 | 240 | 5.45 | 0 | 10 | 1.51 | 84.9 | without Anion |
| 2 wt% Pt-BFO | 16.4 | 0.0034 | 1 | 105 | 270 | 5.45 | 0 | 10 | 0.69 | 93.1 | without Anion |
| 2 wt% Pt-BFO | 16.4 | 0.0034 | 1 | 105 | 0 | 5.45 | 0 | 10 | 10 | 0 | without Anion |
| 2 wt% Pt-BFO | 16.4 | 0.0034 | 1 | 105 | 30 | 5.45 | 0 | 10 | 8.51 | 14.9 | without Anion |
| 2 wt% Pt-BFO | 16.4 | 0.0034 | 1 | 105 | 60 | 5.45 | 0 | 10 | 7.53 | 24.7 | without Anion |
| 2 wt% Pt-BFO | 16.4 | 0.0034 | 1 | 105 | 90 | 5.45 | 0 | 10 | 6.63 | 33.7 | without Anion |
| 2 wt% Pt-BFO | 16.4 | 0.0034 | 1 | 105 | 120 | 5.45 | 0 | 10 | 5.44 | 45.6 | without Anion |
| 2 wt% Pt-BFO | 16.4 | 0.0034 | 1 | 105 | 150 | 5.45 | 0 | 10 | 4.32 | 56.8 | without Anion |
| 2 wt% Pt-BFO | 16.4 | 0.0034 | 1 | 105 | 180 | 5.45 | 0 | 10 | 3.36 | 66.4 | without Anion |
| 2 wt% Pt-BFO | 16.4 | 0.0034 | 1 | 105 | 210 | 5.45 | 0 | 10 | 2.42 | 75.8 | without Anion |
| 2 wt% Pt-BFO | 16.4 | 0.0034 | 1 | 105 | 240 | 5.45 | 0 | 10 | 1.47 | 85.3 | without Anion |
| 2 wt% Pt-BFO | 16.4 | 0.0034 | 1 | 105 | 270 | 5.45 | 0 | 10 | 0.64 | 93.6 | without Anion |
| 2 wt% Pt-BFO | 16.4 | 0.0034 | 1 | 105 | 0 | 5.45 | 0 | 10 | 10 | 0 | without Anion |
| 2 wt% Pt-BFO | 16.4 | 0.0034 | 1 | 105 | 30 | 5.45 | 0 | 10 | 8.54 | 14.6 | without Anion |
| 2 wt% Pt-BFO | 16.4 | 0.0034 | 1 | 105 | 60 | 5.45 | 0 | 10 | 7.57 | 24.3 | without Anion |
| 2 wt% Pt-BFO | 16.4 | 0.0034 | 1 | 105 | 90 | 5.45 | 0 | 10 | 6.67 | 33.3 | without Anion |
| 2 wt% Pt-BFO | 16.4 | 0.0034 | 1 | 105 | 120 | 5.45 | 0 | 10 | 5.48 | 45.2 | without Anion |
| 2 wt% Pt-BFO | 16.4 | 0.0034 | 1 | 105 | 150 | 5.45 | 0 | 10 | 4.34 | 56.6 | without Anion |
| 2 wt% Pt-BFO | 16.4 | 0.0034 | 1 | 105 | 180 | 5.45 | 0 | 10 | 3.4 | 66 | without Anion |
| 2 wt% Pt-BFO | 16.4 | 0.0034 | 1 | 105 | 210 | 5.45 | 0 | 10 | 2.45 | 75.5 | without Anion |
| 2 wt% Pt-BFO | 16.4 | 0.0034 | 1 | 105 | 240 | 5.45 | 0 | 10 | 1.49 | 85.1 | without Anion |
| 2 wt% Pt-BFO | 16.4 | 0.0034 | 1 | 105 | 270 | 5.45 | 0 | 10 | 0.66 | 93.4 | without Anion |
| 2 wt% Pd-BFO | 22.9 | 0.0046 | 1 | 0 | 0 | 5.45 | 0 | 10 | 10 | 0 | without Anion |
| 2 wt% Pd-BFO | 22.9 | 0.0046 | 1 | 0 | 30 | 5.45 | 0 | 10 | 8.55 | 14.5 | without Anion |
| 2 wt% Pd-BFO | 22.9 | 0.0046 | 1 | 0 | 60 | 5.45 | 0 | 10 | 8.43 | 15.7 | without Anion |
| 2 wt% Pd-BFO | 22.9 | 0.0046 | 1 | 0 | 90 | 5.45 | 0 | 10 | 8.38 | 16.2 | without Anion |
| 2 wt% Pd-BFO | 22.9 | 0.0046 | 1 | 0 | 120 | 5.45 | 0 | 10 | 8.34 | 16.6 | without Anion |
| 2 wt% Pd-BFO | 22.9 | 0.0046 | 1 | 0 | 150 | 5.45 | 0 | 10 | 8.31 | 16.9 | without Anion |
| 2 wt% Pd-BFO | 22.9 | 0.0046 | 1 | 0 | 180 | 5.45 | 0 | 10 | 8.26 | 17.4 | without Anion |
| 2 wt% Pd-BFO | 22.9 | 0.0046 | 1 | 0 | 210 | 5.45 | 0 | 10 | 8.22 | 17.8 | without Anion |
| 2 wt% Pd-BFO | 22.9 | 0.0046 | 1 | 0 | 240 | 5.45 | 0 | 10 | 8.2 | 18 | without Anion |
| 2 wt% Pd-BFO | 22.9 | 0.0046 | 1 | 0 | 270 | 5.45 | 0 | 10 | 8.17 | 18.3 | without Anion |
| 2 wt% Pd-BFO | 22.9 | 0.0046 | 1 | 0 | 0 | 5.45 | 0 | 10 | 10 | 0 | without Anion |
| 2 wt% Pd-BFO | 22.9 | 0.0046 | 1 | 0 | 30 | 5.45 | 0 | 10 | 8.5 | 15 | without Anion |
| 2 wt% Pd-BFO | 22.9 | 0.0046 | 1 | 0 | 60 | 5.45 | 0 | 10 | 8.41 | 15.9 | without Anion |
| 2 wt% Pd-BFO | 22.9 | 0.0046 | 1 | 0 | 90 | 5.45 | 0 | 10 | 8.35 | 16.5 | without Anion |
| 2 wt% Pd-BFO | 22.9 | 0.0046 | 1 | 0 | 120 | 5.45 | 0 | 10 | 8.3 | 17 | without Anion |
| 2 wt% Pd-BFO | 22.9 | 0.0046 | 1 | 0 | 150 | 5.45 | 0 | 10 | 8.26 | 17.4 | without Anion |
| 2 wt% Pd-BFO | 22.9 | 0.0046 | 1 | 0 | 180 | 5.45 | 0 | 10 | 8.22 | 17.8 | without Anion |
| 2 wt% Pd-BFO | 22.9 | 0.0046 | 1 | 0 | 210 | 5.45 | 0 | 10 | 8.19 | 18.1 | without Anion |
| 2 wt% Pd-BFO | 22.9 | 0.0046 | 1 | 0 | 240 | 5.45 | 0 | 10 | 8.14 | 18.6 | without Anion |
| 2 wt% Pd-BFO | 22.9 | 0.0046 | 1 | 0 | 270 | 5.45 | 0 | 10 | 8.11 | 18.9 | without Anion |
| 2 wt% Pd-BFO | 22.9 | 0.0046 | 1 | 0 | 0 | 5.45 | 0 | 10 | 10 | 0 | without Anion |
| 2 wt% Pd-BFO | 22.9 | 0.0046 | 1 | 0 | 30 | 5.45 | 0 | 10 | 8.47 | 15.3 | without Anion |
| 2 wt% Pd-BFO | 22.9 | 0.0046 | 1 | 0 | 60 | 5.45 | 0 | 10 | 8.39 | 16.1 | without Anion |
| 2 wt% Pd-BFO | 22.9 | 0.0046 | 1 | 0 | 90 | 5.45 | 0 | 10 | 8.3 | 17 | without Anion |
| 2 wt% Pd-BFO | 22.9 | 0.0046 | 1 | 0 | 120 | 5.45 | 0 | 10 | 8.24 | 17.6 | without Anion |
| 2 wt% Pd-BFO | 22.9 | 0.0046 | 1 | 0 | 150 | 5.45 | 0 | 10 | 8.17 | 18.3 | without Anion |
| 2 wt% Pd-BFO | 22.9 | 0.0046 | 1 | 0 | 180 | 5.45 | 0 | 10 | 8.14 | 18.6 | without Anion |
| 2 wt% Pd-BFO | 22.9 | 0.0046 | 1 | 0 | 210 | 5.45 | 0 | 10 | 8.1 | 19 | without Anion |
| 2 wt% Pd-BFO | 22.9 | 0.0046 | 1 | 0 | 240 | 5.45 | 0 | 10 | 8.06 | 19.4 | without Anion |
| 2 wt% Pd-BFO | 22.9 | 0.0046 | 1 | 0 | 270 | 5.45 | 0 | 10 | 8.05 | 19.5 | without Anion |
| 2 wt% Pd-BFO | 22.9 | 0.0046 | 1 | 25 | 0 | 5.45 | 0 | 10 | 10 | 0 | without Anion |
| 2 wt% Pd-BFO | 22.9 | 0.0046 | 1 | 25 | 30 | 5.45 | 0 | 10 | 9.03 | 9.7 | without Anion |
| 2 wt% Pd-BFO | 22.9 | 0.0046 | 1 | 25 | 60 | 5.45 | 0 | 10 | 8.42 | 15.8 | without Anion |
| 2 wt% Pd-BFO | 22.9 | 0.0046 | 1 | 25 | 90 | 5.45 | 0 | 10 | 7.73 | 22.7 | without Anion |
| 2 wt% Pd-BFO | 22.9 | 0.0046 | 1 | 25 | 120 | 5.45 | 0 | 10 | 7.24 | 27.6 | without Anion |
| 2 wt% Pd-BFO | 22.9 | 0.0046 | 1 | 25 | 150 | 5.45 | 0 | 10 | 6.6 | 34 | without Anion |
| 2 wt% Pd-BFO | 22.9 | 0.0046 | 1 | 25 | 180 | 5.45 | 0 | 10 | 5.97 | 40.3 | without Anion |
| 2 wt% Pd-BFO | 22.9 | 0.0046 | 1 | 25 | 210 | 5.45 | 0 | 10 | 5.39 | 46.1 | without Anion |
| 2 wt% Pd-BFO | 22.9 | 0.0046 | 1 | 25 | 240 | 5.45 | 0 | 10 | 4.93 | 50.7 | without Anion |
| 2 wt% Pd-BFO | 22.9 | 0.0046 | 1 | 25 | 270 | 5.45 | 0 | 10 | 4.5 | 55 | without Anion |
| 2 wt% Pd-BFO | 22.9 | 0.0046 | 1 | 25 | 0 | 5.45 | 0 | 10 | 10 | 0 | without Anion |
| 2 wt% Pd-BFO | 22.9 | 0.0046 | 1 | 25 | 30 | 5.45 | 0 | 10 | 9.08 | 9.2 | without Anion |
| 2 wt% Pd-BFO | 22.9 | 0.0046 | 1 | 25 | 60 | 5.45 | 0 | 10 | 8.47 | 15.3 | without Anion |
| 2 wt% Pd-BFO | 22.9 | 0.0046 | 1 | 25 | 90 | 5.45 | 0 | 10 | 7.78 | 22.2 | without Anion |
| 2 wt% Pd-BFO | 22.9 | 0.0046 | 1 | 25 | 120 | 5.45 | 0 | 10 | 7.29 | 27.1 | without Anion |
| 2 wt% Pd-BFO | 22.9 | 0.0046 | 1 | 25 | 150 | 5.45 | 0 | 10 | 6.65 | 33.5 | without Anion |
| 2 wt% Pd-BFO | 22.9 | 0.0046 | 1 | 25 | 180 | 5.45 | 0 | 10 | 6.01 | 39.9 | without Anion |
| 2 wt% Pd-BFO | 22.9 | 0.0046 | 1 | 25 | 210 | 5.45 | 0 | 10 | 5.43 | 45.7 | without Anion |
| 2 wt% Pd-BFO | 22.9 | 0.0046 | 1 | 25 | 240 | 5.45 | 0 | 10 | 4.98 | 50.2 | without Anion |
| 2 wt% Pd-BFO | 22.9 | 0.0046 | 1 | 25 | 270 | 5.45 | 0 | 10 | 4.54 | 54.6 | without Anion |
| 2 wt% Pd-BFO | 22.9 | 0.0046 | 1 | 25 | 0 | 5.45 | 0 | 10 | 10 | 0 | without Anion |
| 2 wt% Pd-BFO | 22.9 | 0.0046 | 1 | 25 | 30 | 5.45 | 0 | 10 | 9.05 | 9.5 | without Anion |
| 2 wt% Pd-BFO | 22.9 | 0.0046 | 1 | 25 | 60 | 5.45 | 0 | 10 | 8.45 | 15.5 | without Anion |
| 2 wt% Pd-BFO | 22.9 | 0.0046 | 1 | 25 | 90 | 5.45 | 0 | 10 | 7.75 | 22.5 | without Anion |
| 2 wt% Pd-BFO | 22.9 | 0.0046 | 1 | 25 | 120 | 5.45 | 0 | 10 | 7.28 | 27.2 | without Anion |
| 2 wt% Pd-BFO | 22.9 | 0.0046 | 1 | 25 | 150 | 5.45 | 0 | 10 | 6.6 | 34 | without Anion |
| 2 wt% Pd-BFO | 22.9 | 0.0046 | 1 | 25 | 180 | 5.45 | 0 | 10 | 5.95 | 40.5 | without Anion |
| 2 wt% Pd-BFO | 22.9 | 0.0046 | 1 | 25 | 210 | 5.45 | 0 | 10 | 5.4 | 46 | without Anion |
| 2 wt% Pd-BFO | 22.9 | 0.0046 | 1 | 25 | 240 | 5.45 | 0 | 10 | 4.96 | 50.4 | without Anion |
| 2 wt% Pd-BFO | 22.9 | 0.0046 | 1 | 25 | 270 | 5.45 | 0 | 10 | 4.48 | 55.2 | without Anion |
| 2 wt% Pd-BFO | 22.9 | 0.0046 | 1 | 55 | 0 | 5.45 | 0 | 10 | 10 | 0 | without Anion |
| 2 wt% Pd-BFO | 22.9 | 0.0046 | 1 | 55 | 30 | 5.45 | 0 | 10 | 8.94 | 10.6 | without Anion |
| 2 wt% Pd-BFO | 22.9 | 0.0046 | 1 | 55 | 60 | 5.45 | 0 | 10 | 8.25 | 17.5 | without Anion |
| 2 wt% Pd-BFO | 22.9 | 0.0046 | 1 | 55 | 90 | 5.45 | 0 | 10 | 7.57 | 24.3 | without Anion |
| 2 wt% Pd-BFO | 22.9 | 0.0046 | 1 | 55 | 120 | 5.45 | 0 | 10 | 6.83 | 31.7 | without Anion |
| 2 wt% Pd-BFO | 22.9 | 0.0046 | 1 | 55 | 150 | 5.45 | 0 | 10 | 6.15 | 38.5 | without Anion |
| 2 wt% Pd-BFO | 22.9 | 0.0046 | 1 | 55 | 180 | 5.45 | 0 | 10 | 5.47 | 45.3 | without Anion |
| 2 wt% Pd-BFO | 22.9 | 0.0046 | 1 | 55 | 210 | 5.45 | 0 | 10 | 4.76 | 52.4 | without Anion |
| 2 wt% Pd-BFO | 22.9 | 0.0046 | 1 | 55 | 240 | 5.45 | 0 | 10 | 4.01 | 59.9 | without Anion |
| 2 wt% Pd-BFO | 22.9 | 0.0046 | 1 | 55 | 270 | 5.45 | 0 | 10 | 3.32 | 66.8 | without Anion |
| 2 wt% Pd-BFO | 22.9 | 0.0046 | 1 | 55 | 0 | 5.45 | 0 | 10 | 10 | 0 | without Anion |
| 2 wt% Pd-BFO | 22.9 | 0.0046 | 1 | 55 | 30 | 5.45 | 0 | 10 | 8.91 | 10.9 | without Anion |
| 2 wt% Pd-BFO | 22.9 | 0.0046 | 1 | 55 | 60 | 5.45 | 0 | 10 | 8.19 | 18.1 | without Anion |
| 2 wt% Pd-BFO | 22.9 | 0.0046 | 1 | 55 | 90 | 5.45 | 0 | 10 | 7.63 | 23.7 | without Anion |
| 2 wt% Pd-BFO | 22.9 | 0.0046 | 1 | 55 | 120 | 5.45 | 0 | 10 | 6.89 | 31.1 | without Anion |
| 2 wt% Pd-BFO | 22.9 | 0.0046 | 1 | 55 | 150 | 5.45 | 0 | 10 | 6.23 | 37.7 | without Anion |
| 2 wt% Pd-BFO | 22.9 | 0.0046 | 1 | 55 | 180 | 5.45 | 0 | 10 | 5.55 | 44.5 | without Anion |
| 2 wt% Pd-BFO | 22.9 | 0.0046 | 1 | 55 | 210 | 5.45 | 0 | 10 | 4.83 | 51.7 | without Anion |
| 2 wt% Pd-BFO | 22.9 | 0.0046 | 1 | 55 | 240 | 5.45 | 0 | 10 | 4.08 | 59.2 | without Anion |
| 2 wt% Pd-BFO | 22.9 | 0.0046 | 1 | 55 | 270 | 5.45 | 0 | 10 | 3.37 | 66.3 | without Anion |
| 2 wt% Pd-BFO | 22.9 | 0.0046 | 1 | 55 | 0 | 5.45 | 0 | 10 | 10 | 0 | without Anion |
| 2 wt% Pd-BFO | 22.9 | 0.0046 | 1 | 55 | 30 | 5.45 | 0 | 10 | 8.9 | 11 | without Anion |
| 2 wt% Pd-BFO | 22.9 | 0.0046 | 1 | 55 | 60 | 5.45 | 0 | 10 | 8.22 | 17.8 | without Anion |
| 2 wt% Pd-BFO | 22.9 | 0.0046 | 1 | 55 | 90 | 5.45 | 0 | 10 | 7.6 | 24 | without Anion |
| 2 wt% Pd-BFO | 22.9 | 0.0046 | 1 | 55 | 120 | 5.45 | 0 | 10 | 6.85 | 31.5 | without Anion |
| 2 wt% Pd-BFO | 22.9 | 0.0046 | 1 | 55 | 150 | 5.45 | 0 | 10 | 6.2 | 38 | without Anion |
| 2 wt% Pd-BFO | 22.9 | 0.0046 | 1 | 55 | 180 | 5.45 | 0 | 10 | 5.51 | 44.9 | without Anion |
| 2 wt% Pd-BFO | 22.9 | 0.0046 | 1 | 55 | 210 | 5.45 | 0 | 10 | 4.8 | 52 | without Anion |
| 2 wt% Pd-BFO | 22.9 | 0.0046 | 1 | 55 | 240 | 5.45 | 0 | 10 | 4.05 | 59.5 | without Anion |
| 2 wt% Pd-BFO | 22.9 | 0.0046 | 1 | 55 | 270 | 5.45 | 0 | 10 | 3.3 | 67 | without Anion |
| 2 wt% Pd-BFO | 22.9 | 0.0046 | 0.5 | 105 | 0 | 5.45 | 0 | 10 | 10 | 0 | without Anion |
| 2 wt% Pd-BFO | 22.9 | 0.0046 | 0.5 | 105 | 30 | 5.45 | 0 | 10 | 9.63 | 3.7 | without Anion |
| 2 wt% Pd-BFO | 22.9 | 0.0046 | 0.5 | 105 | 60 | 5.45 | 0 | 10 | 9.31 | 6.9 | without Anion |
| 2 wt% Pd-BFO | 22.9 | 0.0046 | 0.5 | 105 | 90 | 5.45 | 0 | 10 | 8.48 | 15.2 | without Anion |
| 2 wt% Pd-BFO | 22.9 | 0.0046 | 0.5 | 105 | 120 | 5.45 | 0 | 10 | 7.42 | 25.8 | without Anion |
| 2 wt% Pd-BFO | 22.9 | 0.0046 | 0.5 | 105 | 150 | 5.45 | 0 | 10 | 6.59 | 34.1 | without Anion |
| 2 wt% Pd-BFO | 22.9 | 0.0046 | 0.5 | 105 | 180 | 5.45 | 0 | 10 | 5.83 | 41.7 | without Anion |
| 2 wt% Pd-BFO | 22.9 | 0.0046 | 0.5 | 105 | 210 | 5.45 | 0 | 10 | 5.11 | 48.9 | without Anion |
| 2 wt% Pd-BFO | 22.9 | 0.0046 | 0.5 | 105 | 240 | 5.45 | 0 | 10 | 4.25 | 57.5 | without Anion |
| 2 wt% Pd-BFO | 22.9 | 0.0046 | 0.5 | 105 | 270 | 5.45 | 0 | 10 | 3.8 | 62 | without Anion |
| 2 wt% Pd-BFO | 22.9 | 0.0046 | 0.5 | 105 | 0 | 5.45 | 0 | 10 | 10 | 0 | without Anion |
| 2 wt% Pd-BFO | 22.9 | 0.0046 | 0.5 | 105 | 30 | 5.45 | 0 | 10 | 9.31 | 6.9 | without Anion |
| 2 wt% Pd-BFO | 22.9 | 0.0046 | 0.5 | 105 | 60 | 5.45 | 0 | 10 | 9.09 | 9.1 | without Anion |
| 2 wt% Pd-BFO | 22.9 | 0.0046 | 0.5 | 105 | 90 | 5.45 | 0 | 10 | 8.5 | 15 | without Anion |
| 2 wt% Pd-BFO | 22.9 | 0.0046 | 0.5 | 105 | 120 | 5.45 | 0 | 10 | 7.55 | 24.5 | without Anion |
| 2 wt% Pd-BFO | 22.9 | 0.0046 | 0.5 | 105 | 150 | 5.45 | 0 | 10 | 6.85 | 31.5 | without Anion |
| 2 wt% Pd-BFO | 22.9 | 0.0046 | 0.5 | 105 | 180 | 5.45 | 0 | 10 | 6.11 | 38.9 | without Anion |
| 2 wt% Pd-BFO | 22.9 | 0.0046 | 0.5 | 105 | 210 | 5.45 | 0 | 10 | 5.26 | 47.4 | without Anion |
| 2 wt% Pd-BFO | 22.9 | 0.0046 | 0.5 | 105 | 240 | 5.45 | 0 | 10 | 4.62 | 53.8 | without Anion |
| 2 wt% Pd-BFO | 22.9 | 0.0046 | 0.5 | 105 | 270 | 5.45 | 0 | 10 | 4.13 | 58.7 | without Anion |
| 2 wt% Pd-BFO | 22.9 | 0.0046 | 0.5 | 105 | 0 | 5.45 | 0 | 10 | 10 | 0 | without Anion |
| 2 wt% Pd-BFO | 22.9 | 0.0046 | 0.5 | 105 | 30 | 5.45 | 0 | 10 | 9.56 | 4.4 | without Anion |
| 2 wt% Pd-BFO | 22.9 | 0.0046 | 0.5 | 105 | 60 | 5.45 | 0 | 10 | 9.22 | 7.8 | without Anion |
| 2 wt% Pd-BFO | 22.9 | 0.0046 | 0.5 | 105 | 90 | 5.45 | 0 | 10 | 8.47 | 15.3 | without Anion |
| 2 wt% Pd-BFO | 22.9 | 0.0046 | 0.5 | 105 | 120 | 5.45 | 0 | 10 | 7.49 | 25.1 | without Anion |
| 2 wt% Pd-BFO | 22.9 | 0.0046 | 0.5 | 105 | 150 | 5.45 | 0 | 10 | 6.68 | 33.2 | without Anion |
| 2 wt% Pd-BFO | 22.9 | 0.0046 | 0.5 | 105 | 180 | 5.45 | 0 | 10 | 5.94 | 40.6 | without Anion |
| 2 wt% Pd-BFO | 22.9 | 0.0046 | 0.5 | 105 | 210 | 5.45 | 0 | 10 | 5.18 | 48.2 | without Anion |
| 2 wt% Pd-BFO | 22.9 | 0.0046 | 0.5 | 105 | 240 | 5.45 | 0 | 10 | 4.39 | 56.1 | without Anion |
| 2 wt% Pd-BFO | 22.9 | 0.0046 | 0.5 | 105 | 270 | 5.45 | 0 | 10 | 3.87 | 61.3 | without Anion |
| 2 wt% Pd-BFO | 22.9 | 0.0046 | 1.5 | 105 | 0 | 5.45 | 0 | 10 | 10 | 0 | without Anion |
| 2 wt% Pd-BFO | 22.9 | 0.0046 | 1.5 | 105 | 30 | 5.45 | 0 | 10 | 8.01 | 19.9 | without Anion |
| 2 wt% Pd-BFO | 22.9 | 0.0046 | 1.5 | 105 | 60 | 5.45 | 0 | 10 | 5.85 | 41.5 | without Anion |
| 2 wt% Pd-BFO | 22.9 | 0.0046 | 1.5 | 105 | 90 | 5.45 | 0 | 10 | 4.32 | 56.8 | without Anion |
| 2 wt% Pd-BFO | 22.9 | 0.0046 | 1.5 | 105 | 120 | 5.45 | 0 | 10 | 3.38 | 66.2 | without Anion |
| 2 wt% Pd-BFO | 22.9 | 0.0046 | 1.5 | 105 | 150 | 5.45 | 0 | 10 | 2.67 | 73.3 | without Anion |
| 2 wt% Pd-BFO | 22.9 | 0.0046 | 1.5 | 105 | 180 | 5.45 | 0 | 10 | 1.83 | 81.7 | without Anion |
| 2 wt% Pd-BFO | 22.9 | 0.0046 | 1.5 | 105 | 210 | 5.45 | 0 | 10 | 0.98 | 90.2 | without Anion |
| 2 wt% Pd-BFO | 22.9 | 0.0046 | 1.5 | 105 | 240 | 5.45 | 0 | 10 | 0.12 | 98.8 | without Anion |
| 2 wt% Pd-BFO | 22.9 | 0.0046 | 1.5 | 105 | 270 | 5.45 | 0 | 10 | 0 | 100 | without Anion |
| 2 wt% Pd-BFO | 22.9 | 0.0046 | 1.5 | 105 | 0 | 5.45 | 0 | 10 | 10 | 0 | without Anion |
| 2 wt% Pd-BFO | 22.9 | 0.0046 | 1.5 | 105 | 30 | 5.45 | 0 | 10 | 8.05 | 19.5 | without Anion |
| 2 wt% Pd-BFO | 22.9 | 0.0046 | 1.5 | 105 | 60 | 5.45 | 0 | 10 | 5.9 | 41 | without Anion |
| 2 wt% Pd-BFO | 22.9 | 0.0046 | 1.5 | 105 | 90 | 5.45 | 0 | 10 | 4.38 | 56.2 | without Anion |
| 2 wt% Pd-BFO | 22.9 | 0.0046 | 1.5 | 105 | 120 | 5.45 | 0 | 10 | 3.31 | 66.9 | without Anion |
| 2 wt% Pd-BFO | 22.9 | 0.0046 | 1.5 | 105 | 150 | 5.45 | 0 | 10 | 2.6 | 74 | without Anion |
| 2 wt% Pd-BFO | 22.9 | 0.0046 | 1.5 | 105 | 180 | 5.45 | 0 | 10 | 1.76 | 82.4 | without Anion |
| 2 wt% Pd-BFO | 22.9 | 0.0046 | 1.5 | 105 | 210 | 5.45 | 0 | 10 | 1.03 | 89.7 | without Anion |
| 2 wt% Pd-BFO | 22.9 | 0.0046 | 1.5 | 105 | 240 | 5.45 | 0 | 10 | 0.17 | 98.3 | without Anion |
| 2 wt% Pd-BFO | 22.9 | 0.0046 | 1.5 | 105 | 270 | 5.45 | 0 | 10 | 0 | 100 | without Anion |
| 2 wt% Pd-BFO | 22.9 | 0.0046 | 1.5 | 105 | 0 | 5.45 | 0 | 10 | 10 | 0 | without Anion |
| 2 wt% Pd-BFO | 22.9 | 0.0046 | 1.5 | 105 | 30 | 5.45 | 0 | 10 | 8.07 | 19.3 | without Anion |
| 2 wt% Pd-BFO | 22.9 | 0.0046 | 1.5 | 105 | 60 | 5.45 | 0 | 10 | 5.87 | 41.3 | without Anion |
| 2 wt% Pd-BFO | 22.9 | 0.0046 | 1.5 | 105 | 90 | 5.45 | 0 | 10 | 4.34 | 56.6 | without Anion |
| 2 wt% Pd-BFO | 22.9 | 0.0046 | 1.5 | 105 | 120 | 5.45 | 0 | 10 | 3.35 | 66.5 | without Anion |
| 2 wt% Pd-BFO | 22.9 | 0.0046 | 1.5 | 105 | 150 | 5.45 | 0 | 10 | 2.63 | 73.7 | without Anion |
| 2 wt% Pd-BFO | 22.9 | 0.0046 | 1.5 | 105 | 180 | 5.45 | 0 | 10 | 1.8 | 82 | without Anion |
| 2 wt% Pd-BFO | 22.9 | 0.0046 | 1.5 | 105 | 210 | 5.45 | 0 | 10 | 1.01 | 89.9 | without Anion |
| 2 wt% Pd-BFO | 22.9 | 0.0046 | 1.5 | 105 | 240 | 5.45 | 0 | 10 | 0.14 | 98.6 | without Anion |
| 2 wt% Pd-BFO | 22.9 | 0.0046 | 1.5 | 105 | 270 | 5.45 | 0 | 10 | 0 | 100 | without Anion |
| 2 wt% Pd-BFO | 22.9 | 0.0046 | 2 | 105 | 0 | 5.45 | 0 | 10 | 10 | 0 | without Anion |
| 2 wt% Pd-BFO | 22.9 | 0.0046 | 2 | 105 | 30 | 5.45 | 0 | 10 | 8.87 | 11.3 | without Anion |
| 2 wt% Pd-BFO | 22.9 | 0.0046 | 2 | 105 | 60 | 5.45 | 0 | 10 | 8.14 | 18.6 | without Anion |
| 2 wt% Pd-BFO | 22.9 | 0.0046 | 2 | 105 | 90 | 5.45 | 0 | 10 | 7.21 | 27.9 | without Anion |
| 2 wt% Pd-BFO | 22.9 | 0.0046 | 2 | 105 | 120 | 5.45 | 0 | 10 | 6.19 | 38.1 | without Anion |
| 2 wt% Pd-BFO | 22.9 | 0.0046 | 2 | 105 | 150 | 5.45 | 0 | 10 | 5.32 | 46.8 | without Anion |
| 2 wt% Pd-BFO | 22.9 | 0.0046 | 2 | 105 | 180 | 5.45 | 0 | 10 | 4.18 | 58.2 | without Anion |
| 2 wt% Pd-BFO | 22.9 | 0.0046 | 2 | 105 | 210 | 5.45 | 0 | 10 | 3.03 | 69.7 | without Anion |
| 2 wt% Pd-BFO | 22.9 | 0.0046 | 2 | 105 | 240 | 5.45 | 0 | 10 | 1.87 | 81.3 | without Anion |
| 2 wt% Pd-BFO | 22.9 | 0.0046 | 2 | 105 | 270 | 5.45 | 0 | 10 | 0.79 | 92.1 | without Anion |
| 2 wt% Pd-BFO | 22.9 | 0.0046 | 2 | 105 | 0 | 5.45 | 0 | 10 | 10 | 0 | without Anion |
| 2 wt% Pd-BFO | 22.9 | 0.0046 | 2 | 105 | 30 | 5.45 | 0 | 10 | 8.77 | 12.3 | without Anion |
| 2 wt% Pd-BFO | 22.9 | 0.0046 | 2 | 105 | 60 | 5.45 | 0 | 10 | 8.04 | 19.6 | without Anion |
| 2 wt% Pd-BFO | 22.9 | 0.0046 | 2 | 105 | 90 | 5.45 | 0 | 10 | 7.11 | 28.9 | without Anion |
| 2 wt% Pd-BFO | 22.9 | 0.0046 | 2 | 105 | 120 | 5.45 | 0 | 10 | 6.08 | 39.2 | without Anion |
| 2 wt% Pd-BFO | 22.9 | 0.0046 | 2 | 105 | 150 | 5.45 | 0 | 10 | 5.19 | 48.1 | without Anion |
| 2 wt% Pd-BFO | 22.9 | 0.0046 | 2 | 105 | 180 | 5.45 | 0 | 10 | 4.09 | 59.1 | without Anion |
| 2 wt% Pd-BFO | 22.9 | 0.0046 | 2 | 105 | 210 | 5.45 | 0 | 10 | 3.01 | 69.9 | without Anion |
| 2 wt% Pd-BFO | 22.9 | 0.0046 | 2 | 105 | 240 | 5.45 | 0 | 10 | 1.92 | 80.8 | without Anion |
| 2 wt% Pd-BFO | 22.9 | 0.0046 | 2 | 105 | 270 | 5.45 | 0 | 10 | 0.71 | 92.9 | without Anion |
| 2 wt% Pd-BFO | 22.9 | 0.0046 | 2 | 105 | 0 | 5.45 | 0 | 10 | 10 | 0 | without Anion |
| 2 wt% Pd-BFO | 22.9 | 0.0046 | 2 | 105 | 30 | 5.45 | 0 | 10 | 8.84 | 11.6 | without Anion |
| 2 wt% Pd-BFO | 22.9 | 0.0046 | 2 | 105 | 60 | 5.45 | 0 | 10 | 8 | 20 | without Anion |
| 2 wt% Pd-BFO | 22.9 | 0.0046 | 2 | 105 | 90 | 5.45 | 0 | 10 | 7.16 | 28.4 | without Anion |
| 2 wt% Pd-BFO | 22.9 | 0.0046 | 2 | 105 | 120 | 5.45 | 0 | 10 | 6.11 | 38.9 | without Anion |
| 2 wt% Pd-BFO | 22.9 | 0.0046 | 2 | 105 | 150 | 5.45 | 0 | 10 | 5.25 | 47.5 | without Anion |
| 2 wt% Pd-BFO | 22.9 | 0.0046 | 2 | 105 | 180 | 5.45 | 0 | 10 | 4.12 | 58.8 | without Anion |
| 2 wt% Pd-BFO | 22.9 | 0.0046 | 2 | 105 | 210 | 5.45 | 0 | 10 | 3 | 70 | without Anion |
| 2 wt% Pd-BFO | 22.9 | 0.0046 | 2 | 105 | 240 | 5.45 | 0 | 10 | 1.95 | 80.5 | without Anion |
| 2 wt% Pd-BFO | 22.9 | 0.0046 | 2 | 105 | 270 | 5.45 | 0 | 10 | 0.68 | 93.2 | without Anion |
| 2 wt% Pd-BFO | 22.9 | 0.0046 | 2.5 | 105 | 0 | 5.45 | 0 | 10 | 10 | 0 | without Anion |
| 2 wt% Pd-BFO | 22.9 | 0.0046 | 2.5 | 105 | 30 | 5.45 | 0 | 10 | 9.36 | 6.4 | without Anion |
| 2 wt% Pd-BFO | 22.9 | 0.0046 | 2.5 | 105 | 60 | 5.45 | 0 | 10 | 8.41 | 15.9 | without Anion |
| 2 wt% Pd-BFO | 22.9 | 0.0046 | 2.5 | 105 | 90 | 5.45 | 0 | 10 | 7.33 | 26.7 | without Anion |
| 2 wt% Pd-BFO | 22.9 | 0.0046 | 2.5 | 105 | 120 | 5.45 | 0 | 10 | 6.27 | 37.3 | without Anion |
| 2 wt% Pd-BFO | 22.9 | 0.0046 | 2.5 | 105 | 150 | 5.45 | 0 | 10 | 5.34 | 46.6 | without Anion |
| 2 wt% Pd-BFO | 22.9 | 0.0046 | 2.5 | 105 | 180 | 5.45 | 0 | 10 | 4.29 | 57.1 | without Anion |
| 2 wt% Pd-BFO | 22.9 | 0.0046 | 2.5 | 105 | 210 | 5.45 | 0 | 10 | 3.41 | 65.9 | without Anion |
| 2 wt% Pd-BFO | 22.9 | 0.0046 | 2.5 | 105 | 240 | 5.45 | 0 | 10 | 2.36 | 76.4 | without Anion |
| 2 wt% Pd-BFO | 22.9 | 0.0046 | 2.5 | 105 | 270 | 5.45 | 0 | 10 | 1.23 | 87.7 | without Anion |
| 2 wt% Pd-BFO | 22.9 | 0.0046 | 2.5 | 105 | 0 | 5.45 | 0 | 10 | 10 | 0 | without Anion |
| 2 wt% Pd-BFO | 22.9 | 0.0046 | 2.5 | 105 | 30 | 5.45 | 0 | 10 | 9.17 | 8.3 | without Anion |
| 2 wt% Pd-BFO | 22.9 | 0.0046 | 2.5 | 105 | 60 | 5.45 | 0 | 10 | 8.29 | 17.1 | without Anion |
| 2 wt% Pd-BFO | 22.9 | 0.0046 | 2.5 | 105 | 90 | 5.45 | 0 | 10 | 7.36 | 26.4 | without Anion |
| 2 wt% Pd-BFO | 22.9 | 0.0046 | 2.5 | 105 | 120 | 5.45 | 0 | 10 | 6.42 | 35.8 | without Anion |
| 2 wt% Pd-BFO | 22.9 | 0.0046 | 2.5 | 105 | 150 | 5.45 | 0 | 10 | 5.47 | 45.3 | without Anion |
| 2 wt% Pd-BFO | 22.9 | 0.0046 | 2.5 | 105 | 180 | 5.45 | 0 | 10 | 4.35 | 56.5 | without Anion |
| 2 wt% Pd-BFO | 22.9 | 0.0046 | 2.5 | 105 | 210 | 5.45 | 0 | 10 | 3.56 | 64.4 | without Anion |
| 2 wt% Pd-BFO | 22.9 | 0.0046 | 2.5 | 105 | 240 | 5.45 | 0 | 10 | 2.49 | 75.1 | without Anion |
| 2 wt% Pd-BFO | 22.9 | 0.0046 | 2.5 | 105 | 270 | 5.45 | 0 | 10 | 1.33 | 86.7 | without Anion |
| 2 wt% Pd-BFO | 22.9 | 0.0046 | 2.5 | 105 | 0 | 5.45 | 0 | 10 | 10 | 0 | without Anion |
| 2 wt% Pd-BFO | 22.9 | 0.0046 | 2.5 | 105 | 30 | 5.45 | 0 | 10 | 9.24 | 7.6 | without Anion |
| 2 wt% Pd-BFO | 22.9 | 0.0046 | 2.5 | 105 | 60 | 5.45 | 0 | 10 | 8.35 | 16.5 | without Anion |
| 2 wt% Pd-BFO | 22.9 | 0.0046 | 2.5 | 105 | 90 | 5.45 | 0 | 10 | 7.3 | 27 | without Anion |
| 2 wt% Pd-BFO | 22.9 | 0.0046 | 2.5 | 105 | 120 | 5.45 | 0 | 10 | 6.39 | 36.1 | without Anion |
| 2 wt% Pd-BFO | 22.9 | 0.0046 | 2.5 | 105 | 150 | 5.45 | 0 | 10 | 5.41 | 45.9 | without Anion |
| 2 wt% Pd-BFO | 22.9 | 0.0046 | 2.5 | 105 | 180 | 5.45 | 0 | 10 | 4.33 | 56.7 | without Anion |
| 2 wt% Pd-BFO | 22.9 | 0.0046 | 2.5 | 105 | 210 | 5.45 | 0 | 10 | 3.47 | 65.3 | without Anion |
| 2 wt% Pd-BFO | 22.9 | 0.0046 | 2.5 | 105 | 240 | 5.45 | 0 | 10 | 2.42 | 75.8 | without Anion |
| 2 wt% Pd-BFO | 22.9 | 0.0046 | 2.5 | 105 | 270 | 5.45 | 0 | 10 | 1.28 | 87.2 | without Anion |
| 2 wt% Pd-BFO | 22.9 | 0.0046 | 1.5 | 105 | 0 | 5.45 | 0 | 5 | 5 | 0 | without Anion |
| 2 wt% Pd-BFO | 22.9 | 0.0046 | 1.5 | 105 | 30 | 5.45 | 0 | 5 | 3.99 | 20.2 | without Anion |
| 2 wt% Pd-BFO | 22.9 | 0.0046 | 1.5 | 105 | 60 | 5.45 | 0 | 5 | 2.75 | 45 | without Anion |
| 2 wt% Pd-BFO | 22.9 | 0.0046 | 1.5 | 105 | 90 | 5.45 | 0 | 5 | 1.87 | 62.6 | without Anion |
| 2 wt% Pd-BFO | 22.9 | 0.0046 | 1.5 | 105 | 120 | 5.45 | 0 | 5 | 1.01 | 79.8 | without Anion |
| 2 wt% Pd-BFO | 22.9 | 0.0046 | 1.5 | 105 | 150 | 5.45 | 0 | 5 | 0.44 | 91.2 | without Anion |
| 2 wt% Pd-BFO | 22.9 | 0.0046 | 1.5 | 105 | 180 | 5.45 | 0 | 5 | 0 | 100 | without Anion |
| 2 wt% Pd-BFO | 22.9 | 0.0046 | 1.5 | 105 | 210 | 5.45 | 0 | 5 | 0 | 100 | without Anion |
| 2 wt% Pd-BFO | 22.9 | 0.0046 | 1.5 | 105 | 240 | 5.45 | 0 | 5 | 0 | 100 | without Anion |
| 2 wt% Pd-BFO | 22.9 | 0.0046 | 1.5 | 105 | 270 | 5.45 | 0 | 5 | 0 | 100 | without Anion |
| 2 wt% Pd-BFO | 22.9 | 0.0046 | 1.5 | 105 | 0 | 5.45 | 0 | 5 | 5 | 0 | without Anion |
| 2 wt% Pd-BFO | 22.9 | 0.0046 | 1.5 | 105 | 30 | 5.45 | 0 | 5 | 4.02 | 19.6 | without Anion |
| 2 wt% Pd-BFO | 22.9 | 0.0046 | 1.5 | 105 | 60 | 5.45 | 0 | 5 | 2.83 | 43.4 | without Anion |
| 2 wt% Pd-BFO | 22.9 | 0.0046 | 1.5 | 105 | 90 | 5.45 | 0 | 5 | 1.98 | 60.4 | without Anion |
| 2 wt% Pd-BFO | 22.9 | 0.0046 | 1.5 | 105 | 120 | 5.45 | 0 | 5 | 1.05 | 79 | without Anion |
| 2 wt% Pd-BFO | 22.9 | 0.0046 | 1.5 | 105 | 150 | 5.45 | 0 | 5 | 0.51 | 89.8 | without Anion |
| 2 wt% Pd-BFO | 22.9 | 0.0046 | 1.5 | 105 | 180 | 5.45 | 0 | 5 | 0.03 | 99.4 | without Anion |
| 2 wt% Pd-BFO | 22.9 | 0.0046 | 1.5 | 105 | 210 | 5.45 | 0 | 5 | 0 | 100 | without Anion |
| 2 wt% Pd-BFO | 22.9 | 0.0046 | 1.5 | 105 | 240 | 5.45 | 0 | 5 | 0 | 100 | without Anion |
| 2 wt% Pd-BFO | 22.9 | 0.0046 | 1.5 | 105 | 270 | 5.45 | 0 | 5 | 0 | 100 | without Anion |
| 2 wt% Pd-BFO | 22.9 | 0.0046 | 1.5 | 105 | 0 | 5.45 | 0 | 5 | 5 | 0 | without Anion |
| 2 wt% Pd-BFO | 22.9 | 0.0046 | 1.5 | 105 | 30 | 5.45 | 0 | 5 | 4.06 | 18.8 | without Anion |
| 2 wt% Pd-BFO | 22.9 | 0.0046 | 1.5 | 105 | 60 | 5.45 | 0 | 5 | 2.85 | 43 | without Anion |
| 2 wt% Pd-BFO | 22.9 | 0.0046 | 1.5 | 105 | 90 | 5.45 | 0 | 5 | 2.02 | 59.6 | without Anion |
| 2 wt% Pd-BFO | 22.9 | 0.0046 | 1.5 | 105 | 120 | 5.45 | 0 | 5 | 1.12 | 77.6 | without Anion |
| 2 wt% Pd-BFO | 22.9 | 0.0046 | 1.5 | 105 | 150 | 5.45 | 0 | 5 | 0.66 | 86.8 | without Anion |
| 2 wt% Pd-BFO | 22.9 | 0.0046 | 1.5 | 105 | 180 | 5.45 | 0 | 5 | 0.09 | 98.2 | without Anion |
| 2 wt% Pd-BFO | 22.9 | 0.0046 | 1.5 | 105 | 210 | 5.45 | 0 | 5 | 0 | 100 | without Anion |
| 2 wt% Pd-BFO | 22.9 | 0.0046 | 1.5 | 105 | 240 | 5.45 | 0 | 5 | 0 | 100 | without Anion |
| 2 wt% Pd-BFO | 22.9 | 0.0046 | 1.5 | 105 | 270 | 5.45 | 0 | 5 | 0 | 100 | without Anion |
| 2 wt% Pd-BFO | 22.9 | 0.0046 | 1.5 | 105 | 0 | 5.45 | 0 | 10 | 10 | 0 | without Anion |
| 2 wt% Pd-BFO | 22.9 | 0.0046 | 1.5 | 105 | 30 | 5.45 | 0 | 10 | 8.01 | 19.9 | without Anion |
| 2 wt% Pd-BFO | 22.9 | 0.0046 | 1.5 | 105 | 60 | 5.45 | 0 | 10 | 5.85 | 41.5 | without Anion |
| 2 wt% Pd-BFO | 22.9 | 0.0046 | 1.5 | 105 | 90 | 5.45 | 0 | 10 | 4.32 | 56.8 | without Anion |
| 2 wt% Pd-BFO | 22.9 | 0.0046 | 1.5 | 105 | 120 | 5.45 | 0 | 10 | 3.38 | 66.2 | without Anion |
| 2 wt% Pd-BFO | 22.9 | 0.0046 | 1.5 | 105 | 150 | 5.45 | 0 | 10 | 2.67 | 73.3 | without Anion |
| 2 wt% Pd-BFO | 22.9 | 0.0046 | 1.5 | 105 | 180 | 5.45 | 0 | 10 | 1.83 | 81.7 | without Anion |
| 2 wt% Pd-BFO | 22.9 | 0.0046 | 1.5 | 105 | 210 | 5.45 | 0 | 10 | 0.98 | 90.2 | without Anion |
| 2 wt% Pd-BFO | 22.9 | 0.0046 | 1.5 | 105 | 240 | 5.45 | 0 | 10 | 0.12 | 98.8 | without Anion |
| 2 wt% Pd-BFO | 22.9 | 0.0046 | 1.5 | 105 | 270 | 5.45 | 0 | 10 | 0 | 100 | without Anion |
| 2 wt% Pd-BFO | 22.9 | 0.0046 | 1.5 | 105 | 0 | 5.45 | 0 | 10 | 10 | 0 | without Anion |
| 2 wt% Pd-BFO | 22.9 | 0.0046 | 1.5 | 105 | 30 | 5.45 | 0 | 10 | 8.05 | 19.5 | without Anion |
| 2 wt% Pd-BFO | 22.9 | 0.0046 | 1.5 | 105 | 60 | 5.45 | 0 | 10 | 5.9 | 41 | without Anion |
| 2 wt% Pd-BFO | 22.9 | 0.0046 | 1.5 | 105 | 90 | 5.45 | 0 | 10 | 4.38 | 56.2 | without Anion |
| 2 wt% Pd-BFO | 22.9 | 0.0046 | 1.5 | 105 | 120 | 5.45 | 0 | 10 | 3.31 | 66.9 | without Anion |
| 2 wt% Pd-BFO | 22.9 | 0.0046 | 1.5 | 105 | 150 | 5.45 | 0 | 10 | 2.6 | 74 | without Anion |
| 2 wt% Pd-BFO | 22.9 | 0.0046 | 1.5 | 105 | 180 | 5.45 | 0 | 10 | 1.76 | 82.4 | without Anion |
| 2 wt% Pd-BFO | 22.9 | 0.0046 | 1.5 | 105 | 210 | 5.45 | 0 | 10 | 1.03 | 89.7 | without Anion |
| 2 wt% Pd-BFO | 22.9 | 0.0046 | 1.5 | 105 | 240 | 5.45 | 0 | 10 | 0.17 | 98.3 | without Anion |
| 2 wt% Pd-BFO | 22.9 | 0.0046 | 1.5 | 105 | 270 | 5.45 | 0 | 10 | 0 | 100 | without Anion |
| 2 wt% Pd-BFO | 22.9 | 0.0046 | 1.5 | 105 | 0 | 5.45 | 0 | 10 | 10 | 0 | without Anion |
| 2 wt% Pd-BFO | 22.9 | 0.0046 | 1.5 | 105 | 30 | 5.45 | 0 | 10 | 8.07 | 19.3 | without Anion |
| 2 wt% Pd-BFO | 22.9 | 0.0046 | 1.5 | 105 | 60 | 5.45 | 0 | 10 | 5.87 | 41.3 | without Anion |
| 2 wt% Pd-BFO | 22.9 | 0.0046 | 1.5 | 105 | 90 | 5.45 | 0 | 10 | 4.34 | 56.6 | without Anion |
| 2 wt% Pd-BFO | 22.9 | 0.0046 | 1.5 | 105 | 120 | 5.45 | 0 | 10 | 3.35 | 66.5 | without Anion |
| 2 wt% Pd-BFO | 22.9 | 0.0046 | 1.5 | 105 | 150 | 5.45 | 0 | 10 | 2.63 | 73.7 | without Anion |
| 2 wt% Pd-BFO | 22.9 | 0.0046 | 1.5 | 105 | 180 | 5.45 | 0 | 10 | 1.8 | 82 | without Anion |
| 2 wt% Pd-BFO | 22.9 | 0.0046 | 1.5 | 105 | 210 | 5.45 | 0 | 10 | 1.01 | 89.9 | without Anion |
| 2 wt% Pd-BFO | 22.9 | 0.0046 | 1.5 | 105 | 240 | 5.45 | 0 | 10 | 0.14 | 98.6 | without Anion |
| 2 wt% Pd-BFO | 22.9 | 0.0046 | 1.5 | 105 | 270 | 5.45 | 0 | 10 | 0 | 100 | without Anion |
| 2 wt% Pd-BFO | 22.9 | 0.0046 | 1.5 | 105 | 0 | 5.45 | 0 | 20 | 20 | 0 | without Anion |
| 2 wt% Pd-BFO | 22.9 | 0.0046 | 1.5 | 105 | 30 | 5.45 | 0 | 20 | 16.39 | 18.05 | without Anion |
| 2 wt% Pd-BFO | 22.9 | 0.0046 | 1.5 | 105 | 60 | 5.45 | 0 | 20 | 14.81 | 25.95 | without Anion |
| 2 wt% Pd-BFO | 22.9 | 0.0046 | 1.5 | 105 | 90 | 5.45 | 0 | 20 | 12.97 | 35.15 | without Anion |
| 2 wt% Pd-BFO | 22.9 | 0.0046 | 1.5 | 105 | 120 | 5.45 | 0 | 20 | 11.15 | 44.25 | without Anion |
| 2 wt% Pd-BFO | 22.9 | 0.0046 | 1.5 | 105 | 150 | 5.45 | 0 | 20 | 9.37 | 53.15 | without Anion |
| 2 wt% Pd-BFO | 22.9 | 0.0046 | 1.5 | 105 | 180 | 5.45 | 0 | 20 | 7.85 | 60.75 | without Anion |
| 2 wt% Pd-BFO | 22.9 | 0.0046 | 1.5 | 105 | 210 | 5.45 | 0 | 20 | 6.59 | 67.05 | without Anion |
| 2 wt% Pd-BFO | 22.9 | 0.0046 | 1.5 | 105 | 240 | 5.45 | 0 | 20 | 5.01 | 74.95 | without Anion |
| 2 wt% Pd-BFO | 22.9 | 0.0046 | 1.5 | 105 | 270 | 5.45 | 0 | 20 | 3.89 | 80.55 | without Anion |
| 2 wt% Pd-BFO | 22.9 | 0.0046 | 1.5 | 105 | 0 | 5.45 | 0 | 20 | 20 | 0 | without Anion |
| 2 wt% Pd-BFO | 22.9 | 0.0046 | 1.5 | 105 | 30 | 5.45 | 0 | 20 | 16.31 | 18.45 | without Anion |
| 2 wt% Pd-BFO | 22.9 | 0.0046 | 1.5 | 105 | 60 | 5.45 | 0 | 20 | 14.74 | 26.3 | without Anion |
| 2 wt% Pd-BFO | 22.9 | 0.0046 | 1.5 | 105 | 90 | 5.45 | 0 | 20 | 12.89 | 35.55 | without Anion |
| 2 wt% Pd-BFO | 22.9 | 0.0046 | 1.5 | 105 | 120 | 5.45 | 0 | 20 | 11.07 | 44.65 | without Anion |
| 2 wt% Pd-BFO | 22.9 | 0.0046 | 1.5 | 105 | 150 | 5.45 | 0 | 20 | 9.29 | 53.55 | without Anion |
| 2 wt% Pd-BFO | 22.9 | 0.0046 | 1.5 | 105 | 180 | 5.45 | 0 | 20 | 7.77 | 61.15 | without Anion |
| 2 wt% Pd-BFO | 22.9 | 0.0046 | 1.5 | 105 | 210 | 5.45 | 0 | 20 | 6.52 | 67.4 | without Anion |
| 2 wt% Pd-BFO | 22.9 | 0.0046 | 1.5 | 105 | 240 | 5.45 | 0 | 20 | 4.94 | 75.3 | without Anion |
| 2 wt% Pd-BFO | 22.9 | 0.0046 | 1.5 | 105 | 270 | 5.45 | 0 | 20 | 3.82 | 80.9 | without Anion |
| 2 wt% Pd-BFO | 22.9 | 0.0046 | 1.5 | 105 | 0 | 5.45 | 0 | 20 | 20 | 0 | without Anion |
| 2 wt% Pd-BFO | 22.9 | 0.0046 | 1.5 | 105 | 30 | 5.45 | 0 | 20 | 16.35 | 18.25 | without Anion |
| 2 wt% Pd-BFO | 22.9 | 0.0046 | 1.5 | 105 | 60 | 5.45 | 0 | 20 | 14.77 | 26.15 | without Anion |
| 2 wt% Pd-BFO | 22.9 | 0.0046 | 1.5 | 105 | 90 | 5.45 | 0 | 20 | 12.92 | 35.4 | without Anion |
| 2 wt% Pd-BFO | 22.9 | 0.0046 | 1.5 | 105 | 120 | 5.45 | 0 | 20 | 11.1 | 44.5 | without Anion |
| 2 wt% Pd-BFO | 22.9 | 0.0046 | 1.5 | 105 | 150 | 5.45 | 0 | 20 | 9.33 | 53.35 | without Anion |
| 2 wt% Pd-BFO | 22.9 | 0.0046 | 1.5 | 105 | 180 | 5.45 | 0 | 20 | 7.8 | 61 | without Anion |
| 2 wt% Pd-BFO | 22.9 | 0.0046 | 1.5 | 105 | 210 | 5.45 | 0 | 20 | 6.55 | 67.25 | without Anion |
| 2 wt% Pd-BFO | 22.9 | 0.0046 | 1.5 | 105 | 240 | 5.45 | 0 | 20 | 4.97 | 75.15 | without Anion |
| 2 wt% Pd-BFO | 22.9 | 0.0046 | 1.5 | 105 | 270 | 5.45 | 0 | 20 | 3.85 | 80.75 | without Anion |
| 2 wt% Pd-BFO | 22.9 | 0.0046 | 1.5 | 105 | 0 | 5.45 | 0 | 40 | 40 | 0 | without Anion |
| 2 wt% Pd-BFO | 22.9 | 0.0046 | 1.5 | 105 | 30 | 5.45 | 0 | 40 | 37.72 | 5.7 | without Anion |
| 2 wt% Pd-BFO | 22.9 | 0.0046 | 1.5 | 105 | 60 | 5.45 | 0 | 40 | 35.03 | 12.425 | without Anion |
| 2 wt% Pd-BFO | 22.9 | 0.0046 | 1.5 | 105 | 90 | 5.45 | 0 | 40 | 33.28 | 16.8 | without Anion |
| 2 wt% Pd-BFO | 22.9 | 0.0046 | 1.5 | 105 | 120 | 5.45 | 0 | 40 | 31.55 | 21.125 | without Anion |
| 2 wt% Pd-BFO | 22.9 | 0.0046 | 1.5 | 105 | 150 | 5.45 | 0 | 40 | 29.74 | 25.65 | without Anion |
| 2 wt% Pd-BFO | 22.9 | 0.0046 | 1.5 | 105 | 180 | 5.45 | 0 | 40 | 28.02 | 29.95 | without Anion |
| 2 wt% Pd-BFO | 22.9 | 0.0046 | 1.5 | 105 | 210 | 5.45 | 0 | 40 | 26.24 | 34.4 | without Anion |
| 2 wt% Pd-BFO | 22.9 | 0.0046 | 1.5 | 105 | 240 | 5.45 | 0 | 40 | 24.57 | 38.575 | without Anion |
| 2 wt% Pd-BFO | 22.9 | 0.0046 | 1.5 | 105 | 270 | 5.45 | 0 | 40 | 23.06 | 42.35 | without Anion |
| 2 wt% Pd-BFO | 22.9 | 0.0046 | 1.5 | 105 | 0 | 5.45 | 0 | 40 | 40 | 0 | without Anion |
| 2 wt% Pd-BFO | 22.9 | 0.0046 | 1.5 | 105 | 30 | 5.45 | 0 | 40 | 37.81 | 5.475 | without Anion |
| 2 wt% Pd-BFO | 22.9 | 0.0046 | 1.5 | 105 | 60 | 5.45 | 0 | 40 | 35.11 | 12.225 | without Anion |
| 2 wt% Pd-BFO | 22.9 | 0.0046 | 1.5 | 105 | 90 | 5.45 | 0 | 40 | 33.37 | 16.575 | without Anion |
| 2 wt% Pd-BFO | 22.9 | 0.0046 | 1.5 | 105 | 120 | 5.45 | 0 | 40 | 31.62 | 20.95 | without Anion |
| 2 wt% Pd-BFO | 22.9 | 0.0046 | 1.5 | 105 | 150 | 5.45 | 0 | 40 | 29.82 | 25.45 | without Anion |
| 2 wt% Pd-BFO | 22.9 | 0.0046 | 1.5 | 105 | 180 | 5.45 | 0 | 40 | 28.11 | 29.725 | without Anion |
| 2 wt% Pd-BFO | 22.9 | 0.0046 | 1.5 | 105 | 210 | 5.45 | 0 | 40 | 26.32 | 34.2 | without Anion |
| 2 wt% Pd-BFO | 22.9 | 0.0046 | 1.5 | 105 | 240 | 5.45 | 0 | 40 | 24.65 | 38.375 | without Anion |
| 2 wt% Pd-BFO | 22.9 | 0.0046 | 1.5 | 105 | 270 | 5.45 | 0 | 40 | 23.12 | 42.2 | without Anion |
| 2 wt% Pd-BFO | 22.9 | 0.0046 | 1.5 | 105 | 0 | 5.45 | 0 | 40 | 40 | 0 | without Anion |
| 2 wt% Pd-BFO | 22.9 | 0.0046 | 1.5 | 105 | 30 | 5.45 | 0 | 40 | 37.75 | 5.625 | without Anion |
| 2 wt% Pd-BFO | 22.9 | 0.0046 | 1.5 | 105 | 60 | 5.45 | 0 | 40 | 35.06 | 12.35 | without Anion |
| 2 wt% Pd-BFO | 22.9 | 0.0046 | 1.5 | 105 | 90 | 5.45 | 0 | 40 | 33.31 | 16.725 | without Anion |
| 2 wt% Pd-BFO | 22.9 | 0.0046 | 1.5 | 105 | 120 | 5.45 | 0 | 40 | 31.6 | 21 | without Anion |
| 2 wt% Pd-BFO | 22.9 | 0.0046 | 1.5 | 105 | 150 | 5.45 | 0 | 40 | 29.77 | 25.575 | without Anion |
| 2 wt% Pd-BFO | 22.9 | 0.0046 | 1.5 | 105 | 180 | 5.45 | 0 | 40 | 28.06 | 29.85 | without Anion |
| 2 wt% Pd-BFO | 22.9 | 0.0046 | 1.5 | 105 | 210 | 5.45 | 0 | 40 | 26.28 | 34.3 | without Anion |
| 2 wt% Pd-BFO | 22.9 | 0.0046 | 1.5 | 105 | 240 | 5.45 | 0 | 40 | 24.6 | 38.5 | without Anion |
| 2 wt% Pd-BFO | 22.9 | 0.0046 | 1.5 | 105 | 270 | 5.45 | 0 | 40 | 23.09 | 42.275 | without Anion |
| 2 wt% Pd-BFO | 22.9 | 0.0046 | 1.5 | 105 | 0 | 5.45 | 0 | 80 | 80 | 0 | without Anion |
| 2 wt% Pd-BFO | 22.9 | 0.0046 | 1.5 | 105 | 30 | 5.45 | 0 | 80 | 77.87 | 2.6625 | without Anion |
| 2 wt% Pd-BFO | 22.9 | 0.0046 | 1.5 | 105 | 60 | 5.45 | 0 | 80 | 76.09 | 4.8875 | without Anion |
| 2 wt% Pd-BFO | 22.9 | 0.0046 | 1.5 | 105 | 90 | 5.45 | 0 | 80 | 74.42 | 6.975 | without Anion |
| 2 wt% Pd-BFO | 22.9 | 0.0046 | 1.5 | 105 | 120 | 5.45 | 0 | 80 | 72.71 | 9.1125 | without Anion |
| 2 wt% Pd-BFO | 22.9 | 0.0046 | 1.5 | 105 | 150 | 5.45 | 0 | 80 | 70.94 | 11.325 | without Anion |
| 2 wt% Pd-BFO | 22.9 | 0.0046 | 1.5 | 105 | 180 | 5.45 | 0 | 80 | 69.33 | 13.3375 | without Anion |
| 2 wt% Pd-BFO | 22.9 | 0.0046 | 1.5 | 105 | 210 | 5.45 | 0 | 80 | 67.59 | 15.5125 | without Anion |
| 2 wt% Pd-BFO | 22.9 | 0.0046 | 1.5 | 105 | 240 | 5.45 | 0 | 80 | 65.82 | 17.725 | without Anion |
| 2 wt% Pd-BFO | 22.9 | 0.0046 | 1.5 | 105 | 270 | 5.45 | 0 | 80 | 64.19 | 19.7625 | without Anion |
| 2 wt% Pd-BFO | 22.9 | 0.0046 | 1.5 | 105 | 0 | 5.45 | 0 | 80 | 80 | 0 | without Anion |
| 2 wt% Pd-BFO | 22.9 | 0.0046 | 1.5 | 105 | 30 | 5.45 | 0 | 80 | 77.95 | 2.5625 | without Anion |
| 2 wt% Pd-BFO | 22.9 | 0.0046 | 1.5 | 105 | 60 | 5.45 | 0 | 80 | 76.17 | 4.7875 | without Anion |
| 2 wt% Pd-BFO | 22.9 | 0.0046 | 1.5 | 105 | 90 | 5.45 | 0 | 80 | 74.49 | 6.8875 | without Anion |
| 2 wt% Pd-BFO | 22.9 | 0.0046 | 1.5 | 105 | 120 | 5.45 | 0 | 80 | 72.78 | 9.025 | without Anion |
| 2 wt% Pd-BFO | 22.9 | 0.0046 | 1.5 | 105 | 150 | 5.45 | 0 | 80 | 71.01 | 11.2375 | without Anion |
| 2 wt% Pd-BFO | 22.9 | 0.0046 | 1.5 | 105 | 180 | 5.45 | 0 | 80 | 69.4 | 13.25 | without Anion |
| 2 wt% Pd-BFO | 22.9 | 0.0046 | 1.5 | 105 | 210 | 5.45 | 0 | 80 | 67.66 | 15.425 | without Anion |
| 2 wt% Pd-BFO | 22.9 | 0.0046 | 1.5 | 105 | 240 | 5.45 | 0 | 80 | 65.89 | 17.6375 | without Anion |
| 2 wt% Pd-BFO | 22.9 | 0.0046 | 1.5 | 105 | 270 | 5.45 | 0 | 80 | 64.26 | 19.675 | without Anion |
| 2 wt% Pd-BFO | 22.9 | 0.0046 | 1.5 | 105 | 0 | 5.45 | 0 | 80 | 80 | 0 | without Anion |
| 2 wt% Pd-BFO | 22.9 | 0.0046 | 1.5 | 105 | 30 | 5.45 | 0 | 80 | 77.9 | 2.625 | without Anion |
| 2 wt% Pd-BFO | 22.9 | 0.0046 | 1.5 | 105 | 60 | 5.45 | 0 | 80 | 76.11 | 4.8625 | without Anion |
| 2 wt% Pd-BFO | 22.9 | 0.0046 | 1.5 | 105 | 90 | 5.45 | 0 | 80 | 74.43 | 6.9625 | without Anion |
| 2 wt% Pd-BFO | 22.9 | 0.0046 | 1.5 | 105 | 120 | 5.45 | 0 | 80 | 72.75 | 9.0625 | without Anion |
| 2 wt% Pd-BFO | 22.9 | 0.0046 | 1.5 | 105 | 150 | 5.45 | 0 | 80 | 70.99 | 11.2625 | without Anion |
| 2 wt% Pd-BFO | 22.9 | 0.0046 | 1.5 | 105 | 180 | 5.45 | 0 | 80 | 69.37 | 13.2875 | without Anion |
| 2 wt% Pd-BFO | 22.9 | 0.0046 | 1.5 | 105 | 210 | 5.45 | 0 | 80 | 67.62 | 15.475 | without Anion |
| 2 wt% Pd-BFO | 22.9 | 0.0046 | 1.5 | 105 | 240 | 5.45 | 0 | 80 | 65.85 | 17.6875 | without Anion |
| 2 wt% Pd-BFO | 22.9 | 0.0046 | 1.5 | 105 | 270 | 5.45 | 0 | 80 | 64.22 | 19.725 | without Anion |
| 2 wt% Pd-BFO | 22.9 | 0.0046 | 1.5 | 105 | 0 | 3 | 0 | 5 | 5 | 0 | without Anion |
| 2 wt% Pd-BFO | 22.9 | 0.0046 | 1.5 | 105 | 30 | 3 | 0 | 5 | 4.58 | 8.4 | without Anion |
| 2 wt% Pd-BFO | 22.9 | 0.0046 | 1.5 | 105 | 60 | 3 | 0 | 5 | 4.13 | 17.4 | without Anion |
| 2 wt% Pd-BFO | 22.9 | 0.0046 | 1.5 | 105 | 90 | 3 | 0 | 5 | 3.77 | 24.6 | without Anion |
| 2 wt% Pd-BFO | 22.9 | 0.0046 | 1.5 | 105 | 120 | 3 | 0 | 5 | 3.32 | 33.6 | without Anion |
| 2 wt% Pd-BFO | 22.9 | 0.0046 | 1.5 | 105 | 150 | 3 | 0 | 5 | 2.89 | 42.2 | without Anion |
| 2 wt% Pd-BFO | 22.9 | 0.0046 | 1.5 | 105 | 180 | 3 | 0 | 5 | 2.44 | 51.2 | without Anion |
| 2 wt% Pd-BFO | 22.9 | 0.0046 | 1.5 | 105 | 210 | 3 | 0 | 5 | 2.01 | 59.8 | without Anion |
| 2 wt% Pd-BFO | 22.9 | 0.0046 | 1.5 | 105 | 240 | 3 | 0 | 5 | 1.57 | 68.6 | without Anion |
| 2 wt% Pd-BFO | 22.9 | 0.0046 | 1.5 | 105 | 270 | 3 | 0 | 5 | 0.93 | 81.4 | without Anion |
| 2 wt% Pd-BFO | 22.9 | 0.0046 | 1.5 | 105 | 0 | 3 | 0 | 5 | 5 | 0 | without Anion |
| 2 wt% Pd-BFO | 22.9 | 0.0046 | 1.5 | 105 | 30 | 3 | 0 | 5 | 4.52 | 9.6 | without Anion |
| 2 wt% Pd-BFO | 22.9 | 0.0046 | 1.5 | 105 | 60 | 3 | 0 | 5 | 4.06 | 18.8 | without Anion |
| 2 wt% Pd-BFO | 22.9 | 0.0046 | 1.5 | 105 | 90 | 3 | 0 | 5 | 3.71 | 25.8 | without Anion |
| 2 wt% Pd-BFO | 22.9 | 0.0046 | 1.5 | 105 | 120 | 3 | 0 | 5 | 3.25 | 35 | without Anion |
| 2 wt% Pd-BFO | 22.9 | 0.0046 | 1.5 | 105 | 150 | 3 | 0 | 5 | 2.81 | 43.8 | without Anion |
| 2 wt% Pd-BFO | 22.9 | 0.0046 | 1.5 | 105 | 180 | 3 | 0 | 5 | 2.37 | 52.6 | without Anion |
| 2 wt% Pd-BFO | 22.9 | 0.0046 | 1.5 | 105 | 210 | 3 | 0 | 5 | 1.94 | 61.2 | without Anion |
| 2 wt% Pd-BFO | 22.9 | 0.0046 | 1.5 | 105 | 240 | 3 | 0 | 5 | 1.5 | 70 | without Anion |
| 2 wt% Pd-BFO | 22.9 | 0.0046 | 1.5 | 105 | 270 | 3 | 0 | 5 | 0.87 | 82.6 | without Anion |
| 2 wt% Pd-BFO | 22.9 | 0.0046 | 1.5 | 105 | 0 | 3 | 0 | 5 | 5 | 0 | without Anion |
| 2 wt% Pd-BFO | 22.9 | 0.0046 | 1.5 | 105 | 30 | 3 | 0 | 5 | 4.55 | 9 | without Anion |
| 2 wt% Pd-BFO | 22.9 | 0.0046 | 1.5 | 105 | 60 | 3 | 0 | 5 | 4.1 | 18 | without Anion |
| 2 wt% Pd-BFO | 22.9 | 0.0046 | 1.5 | 105 | 90 | 3 | 0 | 5 | 3.72 | 25.6 | without Anion |
| 2 wt% Pd-BFO | 22.9 | 0.0046 | 1.5 | 105 | 120 | 3 | 0 | 5 | 3.3 | 34 | without Anion |
| 2 wt% Pd-BFO | 22.9 | 0.0046 | 1.5 | 105 | 150 | 3 | 0 | 5 | 2.83 | 43.4 | without Anion |
| 2 wt% Pd-BFO | 22.9 | 0.0046 | 1.5 | 105 | 180 | 3 | 0 | 5 | 2.4 | 52 | without Anion |
| 2 wt% Pd-BFO | 22.9 | 0.0046 | 1.5 | 105 | 210 | 3 | 0 | 5 | 1.97 | 60.6 | without Anion |
| 2 wt% Pd-BFO | 22.9 | 0.0046 | 1.5 | 105 | 240 | 3 | 0 | 5 | 1.51 | 69.8 | without Anion |
| 2 wt% Pd-BFO | 22.9 | 0.0046 | 1.5 | 105 | 270 | 3 | 0 | 5 | 0.9 | 82 | without Anion |
| 2 wt% Pd-BFO | 22.9 | 0.0046 | 1.5 | 105 | 0 | 5 | 0 | 5 | 5 | 0 | without Anion |
| 2 wt% Pd-BFO | 22.9 | 0.0046 | 1.5 | 105 | 30 | 5 | 0 | 5 | 4.39 | 12.2 | without Anion |
| 2 wt% Pd-BFO | 22.9 | 0.0046 | 1.5 | 105 | 60 | 5 | 0 | 5 | 3.36 | 32.8 | without Anion |
| 2 wt% Pd-BFO | 22.9 | 0.0046 | 1.5 | 105 | 90 | 5 | 0 | 5 | 2.55 | 49 | without Anion |
| 2 wt% Pd-BFO | 22.9 | 0.0046 | 1.5 | 105 | 120 | 5 | 0 | 5 | 1.68 | 66.4 | without Anion |
| 2 wt% Pd-BFO | 22.9 | 0.0046 | 1.5 | 105 | 150 | 5 | 0 | 5 | 1.03 | 79.4 | without Anion |
| 2 wt% Pd-BFO | 22.9 | 0.0046 | 1.5 | 105 | 180 | 5 | 0 | 5 | 0.68 | 86.4 | without Anion |
| 2 wt% Pd-BFO | 22.9 | 0.0046 | 1.5 | 105 | 210 | 5 | 0 | 5 | 0.11 | 97.8 | without Anion |
| 2 wt% Pd-BFO | 22.9 | 0.0046 | 1.5 | 105 | 240 | 5 | 0 | 5 | 0 | 100 | without Anion |
| 2 wt% Pd-BFO | 22.9 | 0.0046 | 1.5 | 105 | 270 | 5 | 0 | 5 | 0 | 100 | without Anion |
| 2 wt% Pd-BFO | 22.9 | 0.0046 | 1.5 | 105 | 0 | 5 | 0 | 5 | 5 | 0 | without Anion |
| 2 wt% Pd-BFO | 22.9 | 0.0046 | 1.5 | 105 | 30 | 5 | 0 | 5 | 4.45 | 11 | without Anion |
| 2 wt% Pd-BFO | 22.9 | 0.0046 | 1.5 | 105 | 60 | 5 | 0 | 5 | 3.42 | 31.6 | without Anion |
| 2 wt% Pd-BFO | 22.9 | 0.0046 | 1.5 | 105 | 90 | 5 | 0 | 5 | 2.61 | 47.8 | without Anion |
| 2 wt% Pd-BFO | 22.9 | 0.0046 | 1.5 | 105 | 120 | 5 | 0 | 5 | 1.73 | 65.4 | without Anion |
| 2 wt% Pd-BFO | 22.9 | 0.0046 | 1.5 | 105 | 150 | 5 | 0 | 5 | 1.08 | 78.4 | without Anion |
| 2 wt% Pd-BFO | 22.9 | 0.0046 | 1.5 | 105 | 180 | 5 | 0 | 5 | 0.75 | 85 | without Anion |
| 2 wt% Pd-BFO | 22.9 | 0.0046 | 1.5 | 105 | 210 | 5 | 0 | 5 | 0.18 | 96.4 | without Anion |
| 2 wt% Pd-BFO | 22.9 | 0.0046 | 1.5 | 105 | 240 | 5 | 0 | 5 | 0 | 100 | without Anion |
| 2 wt% Pd-BFO | 22.9 | 0.0046 | 1.5 | 105 | 270 | 5 | 0 | 5 | 0 | 100 | without Anion |
| 2 wt% Pd-BFO | 22.9 | 0.0046 | 1.5 | 105 | 0 | 5 | 0 | 5 | 5 | 0 | without Anion |
| 2 wt% Pd-BFO | 22.9 | 0.0046 | 1.5 | 105 | 30 | 5 | 0 | 5 | 4.41 | 11.8 | without Anion |
| 2 wt% Pd-BFO | 22.9 | 0.0046 | 1.5 | 105 | 60 | 5 | 0 | 5 | 3.38 | 32.4 | without Anion |
| 2 wt% Pd-BFO | 22.9 | 0.0046 | 1.5 | 105 | 90 | 5 | 0 | 5 | 2.58 | 48.4 | without Anion |
| 2 wt% Pd-BFO | 22.9 | 0.0046 | 1.5 | 105 | 120 | 5 | 0 | 5 | 1.7 | 66 | without Anion |
| 2 wt% Pd-BFO | 22.9 | 0.0046 | 1.5 | 105 | 150 | 5 | 0 | 5 | 1.05 | 79 | without Anion |
| 2 wt% Pd-BFO | 22.9 | 0.0046 | 1.5 | 105 | 180 | 5 | 0 | 5 | 0.7 | 86 | without Anion |
| 2 wt% Pd-BFO | 22.9 | 0.0046 | 1.5 | 105 | 210 | 5 | 0 | 5 | 0.15 | 97 | without Anion |
| 2 wt% Pd-BFO | 22.9 | 0.0046 | 1.5 | 105 | 240 | 5 | 0 | 5 | 0 | 100 | without Anion |
| 2 wt% Pd-BFO | 22.9 | 0.0046 | 1.5 | 105 | 270 | 5 | 0 | 5 | 0 | 100 | without Anion |
| 2 wt% Pd-BFO | 22.9 | 0.0046 | 1.5 | 105 | 0 | 7 | 0 | 5 | 5 | 0 | without Anion |
| 2 wt% Pd-BFO | 22.9 | 0.0046 | 1.5 | 105 | 30 | 7 | 0 | 5 | 4 | 20 | without Anion |
| 2 wt% Pd-BFO | 22.9 | 0.0046 | 1.5 | 105 | 60 | 7 | 0 | 5 | 2.38 | 52.4 | without Anion |
| 2 wt% Pd-BFO | 22.9 | 0.0046 | 1.5 | 105 | 90 | 7 | 0 | 5 | 1.47 | 70.6 | without Anion |
| 2 wt% Pd-BFO | 22.9 | 0.0046 | 1.5 | 105 | 120 | 7 | 0 | 5 | 0.72 | 85.6 | without Anion |
| 2 wt% Pd-BFO | 22.9 | 0.0046 | 1.5 | 105 | 150 | 7 | 0 | 5 | 0.02 | 99.6 | without Anion |
| 2 wt% Pd-BFO | 22.9 | 0.0046 | 1.5 | 105 | 180 | 7 | 0 | 5 | 0 | 100 | without Anion |
| 2 wt% Pd-BFO | 22.9 | 0.0046 | 1.5 | 105 | 210 | 7 | 0 | 5 | 0 | 100 | without Anion |
| 2 wt% Pd-BFO | 22.9 | 0.0046 | 1.5 | 105 | 240 | 7 | 0 | 5 | 0 | 100 | without Anion |
| 2 wt% Pd-BFO | 22.9 | 0.0046 | 1.5 | 105 | 270 | 7 | 0 | 5 | 0 | 100 | without Anion |
| 2 wt% Pd-BFO | 22.9 | 0.0046 | 1.5 | 105 | 0 | 7 | 0 | 5 | 5 | 0 | without Anion |
| 2 wt% Pd-BFO | 22.9 | 0.0046 | 1.5 | 105 | 30 | 7 | 0 | 5 | 3.95 | 21 | without Anion |
| 2 wt% Pd-BFO | 22.9 | 0.0046 | 1.5 | 105 | 60 | 7 | 0 | 5 | 2.41 | 51.8 | without Anion |
| 2 wt% Pd-BFO | 22.9 | 0.0046 | 1.5 | 105 | 90 | 7 | 0 | 5 | 1.55 | 69 | without Anion |
| 2 wt% Pd-BFO | 22.9 | 0.0046 | 1.5 | 105 | 120 | 7 | 0 | 5 | 0.85 | 83 | without Anion |
| 2 wt% Pd-BFO | 22.9 | 0.0046 | 1.5 | 105 | 150 | 7 | 0 | 5 | 0.08 | 98.4 | without Anion |
| 2 wt% Pd-BFO | 22.9 | 0.0046 | 1.5 | 105 | 180 | 7 | 0 | 5 | 0 | 100 | without Anion |
| 2 wt% Pd-BFO | 22.9 | 0.0046 | 1.5 | 105 | 210 | 7 | 0 | 5 | 0 | 100 | without Anion |
| 2 wt% Pd-BFO | 22.9 | 0.0046 | 1.5 | 105 | 240 | 7 | 0 | 5 | 0 | 100 | without Anion |
| 2 wt% Pd-BFO | 22.9 | 0.0046 | 1.5 | 105 | 270 | 7 | 0 | 5 | 0 | 100 | without Anion |
| 2 wt% Pd-BFO | 22.9 | 0.0046 | 1.5 | 105 | 0 | 7 | 0 | 5 | 5 | 0 | without Anion |
| 2 wt% Pd-BFO | 22.9 | 0.0046 | 1.5 | 105 | 30 | 7 | 0 | 5 | 4.02 | 19.6 | without Anion |
| 2 wt% Pd-BFO | 22.9 | 0.0046 | 1.5 | 105 | 60 | 7 | 0 | 5 | 2.44 | 51.2 | without Anion |
| 2 wt% Pd-BFO | 22.9 | 0.0046 | 1.5 | 105 | 90 | 7 | 0 | 5 | 1.5 | 70 | without Anion |
| 2 wt% Pd-BFO | 22.9 | 0.0046 | 1.5 | 105 | 120 | 7 | 0 | 5 | 0.8 | 84 | without Anion |
| 2 wt% Pd-BFO | 22.9 | 0.0046 | 1.5 | 105 | 150 | 7 | 0 | 5 | 0.04 | 99.2 | without Anion |
| 2 wt% Pd-BFO | 22.9 | 0.0046 | 1.5 | 105 | 180 | 7 | 0 | 5 | 0 | 100 | without Anion |
| 2 wt% Pd-BFO | 22.9 | 0.0046 | 1.5 | 105 | 210 | 7 | 0 | 5 | 0 | 100 | without Anion |
| 2 wt% Pd-BFO | 22.9 | 0.0046 | 1.5 | 105 | 240 | 7 | 0 | 5 | 0 | 100 | without Anion |
| 2 wt% Pd-BFO | 22.9 | 0.0046 | 1.5 | 105 | 270 | 7 | 0 | 5 | 0 | 100 | without Anion |
| 2 wt% Pd-BFO | 22.9 | 0.0046 | 1.5 | 105 | 0 | 9 | 0 | 5 | 5 | 0 | without Anion |
| 2 wt% Pd-BFO | 22.9 | 0.0046 | 1.5 | 105 | 30 | 9 | 0 | 5 | 4.59 | 8.2 | without Anion |
| 2 wt% Pd-BFO | 22.9 | 0.0046 | 1.5 | 105 | 60 | 9 | 0 | 5 | 4.01 | 19.8 | without Anion |
| 2 wt% Pd-BFO | 22.9 | 0.0046 | 1.5 | 105 | 90 | 9 | 0 | 5 | 3.47 | 30.6 | without Anion |
| 2 wt% Pd-BFO | 22.9 | 0.0046 | 1.5 | 105 | 120 | 9 | 0 | 5 | 2.84 | 43.2 | without Anion |
| 2 wt% Pd-BFO | 22.9 | 0.0046 | 1.5 | 105 | 150 | 9 | 0 | 5 | 2.11 | 57.8 | without Anion |
| 2 wt% Pd-BFO | 22.9 | 0.0046 | 1.5 | 105 | 180 | 9 | 0 | 5 | 1.66 | 66.8 | without Anion |
| 2 wt% Pd-BFO | 22.9 | 0.0046 | 1.5 | 105 | 210 | 9 | 0 | 5 | 1.01 | 79.8 | without Anion |
| 2 wt% Pd-BFO | 22.9 | 0.0046 | 1.5 | 105 | 240 | 9 | 0 | 5 | 0.54 | 89.2 | without Anion |
| 2 wt% Pd-BFO | 22.9 | 0.0046 | 1.5 | 105 | 270 | 9 | 0 | 5 | 0.11 | 97.8 | without Anion |
| 2 wt% Pd-BFO | 22.9 | 0.0046 | 1.5 | 105 | 0 | 9 | 0 | 5 | 5 | 0 | without Anion |
| 2 wt% Pd-BFO | 22.9 | 0.0046 | 1.5 | 105 | 30 | 9 | 0 | 5 | 4.64 | 7.2 | without Anion |
| 2 wt% Pd-BFO | 22.9 | 0.0046 | 1.5 | 105 | 60 | 9 | 0 | 5 | 4.09 | 18.2 | without Anion |
| 2 wt% Pd-BFO | 22.9 | 0.0046 | 1.5 | 105 | 90 | 9 | 0 | 5 | 3.55 | 29 | without Anion |
| 2 wt% Pd-BFO | 22.9 | 0.0046 | 1.5 | 105 | 120 | 9 | 0 | 5 | 2.89 | 42.2 | without Anion |
| 2 wt% Pd-BFO | 22.9 | 0.0046 | 1.5 | 105 | 150 | 9 | 0 | 5 | 2.18 | 56.4 | without Anion |
| 2 wt% Pd-BFO | 22.9 | 0.0046 | 1.5 | 105 | 180 | 9 | 0 | 5 | 1.73 | 65.4 | without Anion |
| 2 wt% Pd-BFO | 22.9 | 0.0046 | 1.5 | 105 | 210 | 9 | 0 | 5 | 1.07 | 78.6 | without Anion |
| 2 wt% Pd-BFO | 22.9 | 0.0046 | 1.5 | 105 | 240 | 9 | 0 | 5 | 0.61 | 87.8 | without Anion |
| 2 wt% Pd-BFO | 22.9 | 0.0046 | 1.5 | 105 | 270 | 9 | 0 | 5 | 0.19 | 96.2 | without Anion |
| 2 wt% Pd-BFO | 22.9 | 0.0046 | 1.5 | 105 | 0 | 9 | 0 | 5 | 5 | 0 | without Anion |
| 2 wt% Pd-BFO | 22.9 | 0.0046 | 1.5 | 105 | 30 | 9 | 0 | 5 | 4.6 | 8 | without Anion |
| 2 wt% Pd-BFO | 22.9 | 0.0046 | 1.5 | 105 | 60 | 9 | 0 | 5 | 4.07 | 18.6 | without Anion |
| 2 wt% Pd-BFO | 22.9 | 0.0046 | 1.5 | 105 | 90 | 9 | 0 | 5 | 3.5 | 30 | without Anion |
| 2 wt% Pd-BFO | 22.9 | 0.0046 | 1.5 | 105 | 120 | 9 | 0 | 5 | 2.9 | 42 | without Anion |
| 2 wt% Pd-BFO | 22.9 | 0.0046 | 1.5 | 105 | 150 | 9 | 0 | 5 | 2.15 | 57 | without Anion |
| 2 wt% Pd-BFO | 22.9 | 0.0046 | 1.5 | 105 | 180 | 9 | 0 | 5 | 1.7 | 66 | without Anion |
| 2 wt% Pd-BFO | 22.9 | 0.0046 | 1.5 | 105 | 210 | 9 | 0 | 5 | 1.03 | 79.4 | without Anion |
| 2 wt% Pd-BFO | 22.9 | 0.0046 | 1.5 | 105 | 240 | 9 | 0 | 5 | 0.58 | 88.4 | without Anion |
| 2 wt% Pd-BFO | 22.9 | 0.0046 | 1.5 | 105 | 270 | 9 | 0 | 5 | 0.15 | 97 | without Anion |
| 2 wt% Pd-BFO | 22.9 | 0.0046 | 1.5 | 105 | 0 | 7 | 2 | 5 | 5 | 0 | without Anion |
| 2 wt% Pd-BFO | 22.9 | 0.0046 | 1.5 | 105 | 30 | 7 | 2 | 5 | 4.26 | 14.8 | without Anion |
| 2 wt% Pd-BFO | 22.9 | 0.0046 | 1.5 | 105 | 60 | 7 | 2 | 5 | 2.98 | 40.4 | without Anion |
| 2 wt% Pd-BFO | 22.9 | 0.0046 | 1.5 | 105 | 90 | 7 | 2 | 5 | 2.22 | 55.6 | without Anion |
| 2 wt% Pd-BFO | 22.9 | 0.0046 | 1.5 | 105 | 120 | 7 | 2 | 5 | 1.44 | 71.2 | without Anion |
| 2 wt% Pd-BFO | 22.9 | 0.0046 | 1.5 | 105 | 150 | 7 | 2 | 5 | 0.75 | 85 | without Anion |
| 2 wt% Pd-BFO | 22.9 | 0.0046 | 1.5 | 105 | 180 | 7 | 2 | 5 | 0.22 | 95.6 | without Anion |
| 2 wt% Pd-BFO | 22.9 | 0.0046 | 1.5 | 105 | 210 | 7 | 2 | 5 | 0 | 100 | without Anion |
| 2 wt% Pd-BFO | 22.9 | 0.0046 | 1.5 | 105 | 240 | 7 | 2 | 5 | 0 | 100 | without Anion |
| 2 wt% Pd-BFO | 22.9 | 0.0046 | 1.5 | 105 | 270 | 7 | 2 | 5 | 0 | 100 | without Anion |
| 2 wt% Pd-BFO | 22.9 | 0.0046 | 1.5 | 105 | 0 | 7 | 2 | 5 | 5 | 0 | without Anion |
| 2 wt% Pd-BFO | 22.9 | 0.0046 | 1.5 | 105 | 30 | 7 | 2 | 5 | 4.31 | 13.8 | without Anion |
| 2 wt% Pd-BFO | 22.9 | 0.0046 | 1.5 | 105 | 60 | 7 | 2 | 5 | 3.03 | 39.4 | without Anion |
| 2 wt% Pd-BFO | 22.9 | 0.0046 | 1.5 | 105 | 90 | 7 | 2 | 5 | 2.28 | 54.4 | without Anion |
| 2 wt% Pd-BFO | 22.9 | 0.0046 | 1.5 | 105 | 120 | 7 | 2 | 5 | 1.51 | 69.8 | without Anion |
| 2 wt% Pd-BFO | 22.9 | 0.0046 | 1.5 | 105 | 150 | 7 | 2 | 5 | 0.82 | 83.6 | without Anion |
| 2 wt% Pd-BFO | 22.9 | 0.0046 | 1.5 | 105 | 180 | 7 | 2 | 5 | 0.29 | 94.2 | without Anion |
| 2 wt% Pd-BFO | 22.9 | 0.0046 | 1.5 | 105 | 210 | 7 | 2 | 5 | 0.02 | 99.6 | without Anion |
| 2 wt% Pd-BFO | 22.9 | 0.0046 | 1.5 | 105 | 240 | 7 | 2 | 5 | 0 | 100 | without Anion |
| 2 wt% Pd-BFO | 22.9 | 0.0046 | 1.5 | 105 | 270 | 7 | 2 | 5 | 0 | 100 | without Anion |
| 2 wt% Pd-BFO | 22.9 | 0.0046 | 1.5 | 105 | 0 | 7 | 2 | 5 | 5 | 0 | without Anion |
| 2 wt% Pd-BFO | 22.9 | 0.0046 | 1.5 | 105 | 30 | 7 | 2 | 5 | 4.33 | 13.4 | without Anion |
| 2 wt% Pd-BFO | 22.9 | 0.0046 | 1.5 | 105 | 60 | 7 | 2 | 5 | 3.06 | 38.8 | without Anion |
| 2 wt% Pd-BFO | 22.9 | 0.0046 | 1.5 | 105 | 90 | 7 | 2 | 5 | 2.31 | 53.8 | without Anion |
| 2 wt% Pd-BFO | 22.9 | 0.0046 | 1.5 | 105 | 120 | 7 | 2 | 5 | 1.55 | 69 | without Anion |
| 2 wt% Pd-BFO | 22.9 | 0.0046 | 1.5 | 105 | 150 | 7 | 2 | 5 | 0.85 | 83 | without Anion |
| 2 wt% Pd-BFO | 22.9 | 0.0046 | 1.5 | 105 | 180 | 7 | 2 | 5 | 0.35 | 93 | without Anion |
| 2 wt% Pd-BFO | 22.9 | 0.0046 | 1.5 | 105 | 210 | 7 | 2 | 5 | 0.03 | 99.4 | without Anion |
| 2 wt% Pd-BFO | 22.9 | 0.0046 | 1.5 | 105 | 240 | 7 | 2 | 5 | 0 | 100 | without Anion |
| 2 wt% Pd-BFO | 22.9 | 0.0046 | 1.5 | 105 | 270 | 7 | 2 | 5 | 0 | 100 | without Anion |
| 2 wt% Pd-BFO | 22.9 | 0.0046 | 1.5 | 105 | 0 | 7 | 5 | 5 | 5 | 0 | without Anion |
| 2 wt% Pd-BFO | 22.9 | 0.0046 | 1.5 | 105 | 30 | 7 | 5 | 5 | 4.41 | 11.8 | without Anion |
| 2 wt% Pd-BFO | 22.9 | 0.0046 | 1.5 | 105 | 60 | 7 | 5 | 5 | 3.24 | 35.2 | without Anion |
| 2 wt% Pd-BFO | 22.9 | 0.0046 | 1.5 | 105 | 90 | 7 | 5 | 5 | 2.55 | 49 | without Anion |
| 2 wt% Pd-BFO | 22.9 | 0.0046 | 1.5 | 105 | 120 | 7 | 5 | 5 | 1.73 | 65.4 | without Anion |
| 2 wt% Pd-BFO | 22.9 | 0.0046 | 1.5 | 105 | 150 | 7 | 5 | 5 | 1.01 | 79.8 | without Anion |
| 2 wt% Pd-BFO | 22.9 | 0.0046 | 1.5 | 105 | 180 | 7 | 5 | 5 | 0.57 | 88.6 | without Anion |
| 2 wt% Pd-BFO | 22.9 | 0.0046 | 1.5 | 105 | 210 | 7 | 5 | 5 | 0.11 | 97.8 | without Anion |
| 2 wt% Pd-BFO | 22.9 | 0.0046 | 1.5 | 105 | 240 | 7 | 5 | 5 | 0.1 | 98 | without Anion |
| 2 wt% Pd-BFO | 22.9 | 0.0046 | 1.5 | 105 | 270 | 7 | 5 | 5 | 0 | 100 | without Anion |
| 2 wt% Pd-BFO | 22.9 | 0.0046 | 1.5 | 105 | 0 | 7 | 5 | 5 | 5 | 0 | without Anion |
| 2 wt% Pd-BFO | 22.9 | 0.0046 | 1.5 | 105 | 30 | 7 | 5 | 5 | 4.47 | 10.6 | without Anion |
| 2 wt% Pd-BFO | 22.9 | 0.0046 | 1.5 | 105 | 60 | 7 | 5 | 5 | 3.29 | 34.2 | without Anion |
| 2 wt% Pd-BFO | 22.9 | 0.0046 | 1.5 | 105 | 90 | 7 | 5 | 5 | 2.61 | 47.8 | without Anion |
| 2 wt% Pd-BFO | 22.9 | 0.0046 | 1.5 | 105 | 120 | 7 | 5 | 5 | 1.76 | 64.8 | without Anion |
| 2 wt% Pd-BFO | 22.9 | 0.0046 | 1.5 | 105 | 150 | 7 | 5 | 5 | 1.08 | 78.4 | without Anion |
| 2 wt% Pd-BFO | 22.9 | 0.0046 | 1.5 | 105 | 180 | 7 | 5 | 5 | 0.64 | 87.2 | without Anion |
| 2 wt% Pd-BFO | 22.9 | 0.0046 | 1.5 | 105 | 210 | 7 | 5 | 5 | 0.14 | 97.2 | without Anion |
| 2 wt% Pd-BFO | 22.9 | 0.0046 | 1.5 | 105 | 240 | 7 | 5 | 5 | 0 | 100 | without Anion |
| 2 wt% Pd-BFO | 22.9 | 0.0046 | 1.5 | 105 | 270 | 7 | 5 | 5 | 0 | 100 | without Anion |
| 2 wt% Pd-BFO | 22.9 | 0.0046 | 1.5 | 105 | 0 | 7 | 5 | 5 | 5 | 0 | without Anion |
| 2 wt% Pd-BFO | 22.9 | 0.0046 | 1.5 | 105 | 30 | 7 | 5 | 5 | 4.43 | 11.4 | without Anion |
| 2 wt% Pd-BFO | 22.9 | 0.0046 | 1.5 | 105 | 60 | 7 | 5 | 5 | 3.2 | 36 | without Anion |
| 2 wt% Pd-BFO | 22.9 | 0.0046 | 1.5 | 105 | 90 | 7 | 5 | 5 | 2.51 | 49.8 | without Anion |
| 2 wt% Pd-BFO | 22.9 | 0.0046 | 1.5 | 105 | 120 | 7 | 5 | 5 | 1.69 | 66.2 | without Anion |
| 2 wt% Pd-BFO | 22.9 | 0.0046 | 1.5 | 105 | 150 | 7 | 5 | 5 | 0.99 | 80.2 | without Anion |
| 2 wt% Pd-BFO | 22.9 | 0.0046 | 1.5 | 105 | 180 | 7 | 5 | 5 | 0.52 | 89.6 | without Anion |
| 2 wt% Pd-BFO | 22.9 | 0.0046 | 1.5 | 105 | 210 | 7 | 5 | 5 | 0.04 | 99.2 | without Anion |
| 2 wt% Pd-BFO | 22.9 | 0.0046 | 1.5 | 105 | 240 | 7 | 5 | 5 | 0 | 100 | without Anion |
| 2 wt% Pd-BFO | 22.9 | 0.0046 | 1.5 | 105 | 270 | 7 | 5 | 5 | 0 | 100 | without Anion |
| 2 wt% Pd-BFO | 22.9 | 0.0046 | 1.5 | 105 | 0 | 7 | 7 | 5 | 5 | 0 | without Anion |
| 2 wt% Pd-BFO | 22.9 | 0.0046 | 1.5 | 105 | 30 | 7 | 7 | 5 | 4.77 | 4.6 | without Anion |
| 2 wt% Pd-BFO | 22.9 | 0.0046 | 1.5 | 105 | 60 | 7 | 7 | 5 | 3.98 | 20.4 | without Anion |
| 2 wt% Pd-BFO | 22.9 | 0.0046 | 1.5 | 105 | 90 | 7 | 7 | 5 | 3.14 | 37.2 | without Anion |
| 2 wt% Pd-BFO | 22.9 | 0.0046 | 1.5 | 105 | 120 | 7 | 7 | 5 | 2.46 | 50.8 | without Anion |
| 2 wt% Pd-BFO | 22.9 | 0.0046 | 1.5 | 105 | 150 | 7 | 7 | 5 | 1.72 | 65.6 | without Anion |
| 2 wt% Pd-BFO | 22.9 | 0.0046 | 1.5 | 105 | 180 | 7 | 7 | 5 | 1.21 | 75.8 | without Anion |
| 2 wt% Pd-BFO | 22.9 | 0.0046 | 1.5 | 105 | 210 | 7 | 7 | 5 | 0.84 | 83.2 | without Anion |
| 2 wt% Pd-BFO | 22.9 | 0.0046 | 1.5 | 105 | 240 | 7 | 7 | 5 | 0.42 | 91.6 | without Anion |
| 2 wt% Pd-BFO | 22.9 | 0.0046 | 1.5 | 105 | 270 | 7 | 7 | 5 | 0.05 | 99 | without Anion |
| 2 wt% Pd-BFO | 22.9 | 0.0046 | 1.5 | 105 | 0 | 7 | 7 | 5 | 5 | 0 | without Anion |
| 2 wt% Pd-BFO | 22.9 | 0.0046 | 1.5 | 105 | 30 | 7 | 7 | 5 | 4.8 | 4 | without Anion |
| 2 wt% Pd-BFO | 22.9 | 0.0046 | 1.5 | 105 | 60 | 7 | 7 | 5 | 4.05 | 19 | without Anion |
| 2 wt% Pd-BFO | 22.9 | 0.0046 | 1.5 | 105 | 90 | 7 | 7 | 5 | 3.21 | 35.8 | without Anion |
| 2 wt% Pd-BFO | 22.9 | 0.0046 | 1.5 | 105 | 120 | 7 | 7 | 5 | 2.52 | 49.6 | without Anion |
| 2 wt% Pd-BFO | 22.9 | 0.0046 | 1.5 | 105 | 150 | 7 | 7 | 5 | 1.81 | 63.8 | without Anion |
| 2 wt% Pd-BFO | 22.9 | 0.0046 | 1.5 | 105 | 180 | 7 | 7 | 5 | 1.27 | 74.6 | without Anion |
| 2 wt% Pd-BFO | 22.9 | 0.0046 | 1.5 | 105 | 210 | 7 | 7 | 5 | 0.91 | 81.8 | without Anion |
| 2 wt% Pd-BFO | 22.9 | 0.0046 | 1.5 | 105 | 240 | 7 | 7 | 5 | 0.49 | 90.2 | without Anion |
| 2 wt% Pd-BFO | 22.9 | 0.0046 | 1.5 | 105 | 270 | 7 | 7 | 5 | 0.1 | 98 | without Anion |
| 2 wt% Pd-BFO | 22.9 | 0.0046 | 1.5 | 105 | 0 | 7 | 7 | 5 | 5 | 0 | without Anion |
| 2 wt% Pd-BFO | 22.9 | 0.0046 | 1.5 | 105 | 30 | 7 | 7 | 5 | 4.75 | 5 | without Anion |
| 2 wt% Pd-BFO | 22.9 | 0.0046 | 1.5 | 105 | 60 | 7 | 7 | 5 | 3.95 | 21 | without Anion |
| 2 wt% Pd-BFO | 22.9 | 0.0046 | 1.5 | 105 | 90 | 7 | 7 | 5 | 3.08 | 38.4 | without Anion |
| 2 wt% Pd-BFO | 22.9 | 0.0046 | 1.5 | 105 | 120 | 7 | 7 | 5 | 2.42 | 51.6 | without Anion |
| 2 wt% Pd-BFO | 22.9 | 0.0046 | 1.5 | 105 | 150 | 7 | 7 | 5 | 1.68 | 66.4 | without Anion |
| 2 wt% Pd-BFO | 22.9 | 0.0046 | 1.5 | 105 | 180 | 7 | 7 | 5 | 1.18 | 76.4 | without Anion |
| 2 wt% Pd-BFO | 22.9 | 0.0046 | 1.5 | 105 | 210 | 7 | 7 | 5 | 0.79 | 84.2 | without Anion |
| 2 wt% Pd-BFO | 22.9 | 0.0046 | 1.5 | 105 | 240 | 7 | 7 | 5 | 0.39 | 92.2 | without Anion |
| 2 wt% Pd-BFO | 22.9 | 0.0046 | 1.5 | 105 | 270 | 7 | 7 | 5 | 0 | 100 | without Anion |
| 2 wt% Pd-BFO | 22.9 | 0.0046 | 1.5 | 105 | 0 | 7 | 10 | 5 | 5 | 0 | without Anion |
| 2 wt% Pd-BFO | 22.9 | 0.0046 | 1.5 | 105 | 30 | 7 | 10 | 5 | 4.92 | 1.6 | without Anion |
| 2 wt% Pd-BFO | 22.9 | 0.0046 | 1.5 | 105 | 60 | 7 | 10 | 5 | 4.34 | 13.2 | without Anion |
| 2 wt% Pd-BFO | 22.9 | 0.0046 | 1.5 | 105 | 90 | 7 | 10 | 5 | 3.72 | 25.6 | without Anion |
| 2 wt% Pd-BFO | 22.9 | 0.0046 | 1.5 | 105 | 120 | 7 | 10 | 5 | 3.26 | 34.8 | without Anion |
| 2 wt% Pd-BFO | 22.9 | 0.0046 | 1.5 | 105 | 150 | 7 | 10 | 5 | 2.81 | 43.8 | without Anion |
| 2 wt% Pd-BFO | 22.9 | 0.0046 | 1.5 | 105 | 180 | 7 | 10 | 5 | 2.19 | 56.2 | without Anion |
| 2 wt% Pd-BFO | 22.9 | 0.0046 | 1.5 | 105 | 210 | 7 | 10 | 5 | 1.67 | 66.6 | without Anion |
| 2 wt% Pd-BFO | 22.9 | 0.0046 | 1.5 | 105 | 240 | 7 | 10 | 5 | 1.05 | 79 | without Anion |
| 2 wt% Pd-BFO | 22.9 | 0.0046 | 1.5 | 105 | 270 | 7 | 10 | 5 | 0.47 | 90.6 | without Anion |
| 2 wt% Pd-BFO | 22.9 | 0.0046 | 1.5 | 105 | 0 | 7 | 10 | 5 | 5 | 0 | without Anion |
| 2 wt% Pd-BFO | 22.9 | 0.0046 | 1.5 | 105 | 30 | 7 | 10 | 5 | 4.89 | 2.2 | without Anion |
| 2 wt% Pd-BFO | 22.9 | 0.0046 | 1.5 | 105 | 60 | 7 | 10 | 5 | 4.29 | 14.2 | without Anion |
| 2 wt% Pd-BFO | 22.9 | 0.0046 | 1.5 | 105 | 90 | 7 | 10 | 5 | 3.68 | 26.4 | without Anion |
| 2 wt% Pd-BFO | 22.9 | 0.0046 | 1.5 | 105 | 120 | 7 | 10 | 5 | 3.2 | 36 | without Anion |
| 2 wt% Pd-BFO | 22.9 | 0.0046 | 1.5 | 105 | 150 | 7 | 10 | 5 | 2.75 | 45 | without Anion |
| 2 wt% Pd-BFO | 22.9 | 0.0046 | 1.5 | 105 | 180 | 7 | 10 | 5 | 2.13 | 57.4 | without Anion |
| 2 wt% Pd-BFO | 22.9 | 0.0046 | 1.5 | 105 | 210 | 7 | 10 | 5 | 1.6 | 68 | without Anion |
| 2 wt% Pd-BFO | 22.9 | 0.0046 | 1.5 | 105 | 240 | 7 | 10 | 5 | 0.99 | 80.2 | without Anion |
| 2 wt% Pd-BFO | 22.9 | 0.0046 | 1.5 | 105 | 270 | 7 | 10 | 5 | 0.42 | 91.6 | without Anion |
| 2 wt% Pd-BFO | 22.9 | 0.0046 | 1.5 | 105 | 0 | 7 | 10 | 5 | 5 | 0 | without Anion |
| 2 wt% Pd-BFO | 22.9 | 0.0046 | 1.5 | 105 | 30 | 7 | 10 | 5 | 4.85 | 3 | without Anion |
| 2 wt% Pd-BFO | 22.9 | 0.0046 | 1.5 | 105 | 60 | 7 | 10 | 5 | 4.31 | 13.8 | without Anion |
| 2 wt% Pd-BFO | 22.9 | 0.0046 | 1.5 | 105 | 90 | 7 | 10 | 5 | 3.66 | 26.8 | without Anion |
| 2 wt% Pd-BFO | 22.9 | 0.0046 | 1.5 | 105 | 120 | 7 | 10 | 5 | 3.23 | 35.4 | without Anion |
| 2 wt% Pd-BFO | 22.9 | 0.0046 | 1.5 | 105 | 150 | 7 | 10 | 5 | 2.77 | 44.6 | without Anion |
| 2 wt% Pd-BFO | 22.9 | 0.0046 | 1.5 | 105 | 180 | 7 | 10 | 5 | 2.16 | 56.8 | without Anion |
| 2 wt% Pd-BFO | 22.9 | 0.0046 | 1.5 | 105 | 210 | 7 | 10 | 5 | 1.62 | 67.6 | without Anion |
| 2 wt% Pd-BFO | 22.9 | 0.0046 | 1.5 | 105 | 240 | 7 | 10 | 5 | 1.01 | 79.8 | without Anion |
| 2 wt% Pd-BFO | 22.9 | 0.0046 | 1.5 | 105 | 270 | 7 | 10 | 5 | 0.45 | 91 | without Anion |
| 2 wt% Pd-BFO | 22.9 | 0.0046 | 1.5 | 105 | 0 | 7 | 0 | 5 | 5 | 0 | NaCl |
| 2 wt% Pd-BFO | 22.9 | 0.0046 | 1.5 | 105 | 30 | 7 | 0 | 5 | 4.56 | 8.8 | NaCl |
| 2 wt% Pd-BFO | 22.9 | 0.0046 | 1.5 | 105 | 60 | 7 | 0 | 5 | 4.21 | 15.8 | NaCl |
| 2 wt% Pd-BFO | 22.9 | 0.0046 | 1.5 | 105 | 90 | 7 | 0 | 5 | 3.88 | 22.4 | NaCl |
| 2 wt% Pd-BFO | 22.9 | 0.0046 | 1.5 | 105 | 120 | 7 | 0 | 5 | 3.5 | 30 | NaCl |
| 2 wt% Pd-BFO | 22.9 | 0.0046 | 1.5 | 105 | 150 | 7 | 0 | 5 | 3.14 | 37.2 | NaCl |
| 2 wt% Pd-BFO | 22.9 | 0.0046 | 1.5 | 105 | 180 | 7 | 0 | 5 | 2.8 | 44 | NaCl |
| 2 wt% Pd-BFO | 22.9 | 0.0046 | 1.5 | 105 | 210 | 7 | 0 | 5 | 2.48 | 50.4 | NaCl |
| 2 wt% Pd-BFO | 22.9 | 0.0046 | 1.5 | 105 | 240 | 7 | 0 | 5 | 2.15 | 57 | NaCl |
| 2 wt% Pd-BFO | 22.9 | 0.0046 | 1.5 | 105 | 270 | 7 | 0 | 5 | 1.89 | 62.2 | NaCl |
| 2 wt% Pd-BFO | 22.9 | 0.0046 | 1.5 | 105 | 0 | 7 | 0 | 5 | 5 | 0 | NaCl |
| 2 wt% Pd-BFO | 22.9 | 0.0046 | 1.5 | 105 | 30 | 7 | 0 | 5 | 4.53 | 9.4 | NaCl |
| 2 wt% Pd-BFO | 22.9 | 0.0046 | 1.5 | 105 | 60 | 7 | 0 | 5 | 4.24 | 15.2 | NaCl |
| 2 wt% Pd-BFO | 22.9 | 0.0046 | 1.5 | 105 | 90 | 7 | 0 | 5 | 3.86 | 22.8 | NaCl |
| 2 wt% Pd-BFO | 22.9 | 0.0046 | 1.5 | 105 | 120 | 7 | 0 | 5 | 3.46 | 30.8 | NaCl |
| 2 wt% Pd-BFO | 22.9 | 0.0046 | 1.5 | 105 | 150 | 7 | 0 | 5 | 3.11 | 37.8 | NaCl |
| 2 wt% Pd-BFO | 22.9 | 0.0046 | 1.5 | 105 | 180 | 7 | 0 | 5 | 2.76 | 44.8 | NaCl |
| 2 wt% Pd-BFO | 22.9 | 0.0046 | 1.5 | 105 | 210 | 7 | 0 | 5 | 2.45 | 51 | NaCl |
| 2 wt% Pd-BFO | 22.9 | 0.0046 | 1.5 | 105 | 240 | 7 | 0 | 5 | 2.11 | 57.8 | NaCl |
| 2 wt% Pd-BFO | 22.9 | 0.0046 | 1.5 | 105 | 270 | 7 | 0 | 5 | 1.86 | 62.8 | NaCl |
| 2 wt% Pd-BFO | 22.9 | 0.0046 | 1.5 | 105 | 0 | 7 | 0 | 5 | 5 | 0 | NaCl |
| 2 wt% Pd-BFO | 22.9 | 0.0046 | 1.5 | 105 | 30 | 7 | 0 | 5 | 4.5 | 10 | NaCl |
| 2 wt% Pd-BFO | 22.9 | 0.0046 | 1.5 | 105 | 60 | 7 | 0 | 5 | 4.19 | 16.2 | NaCl |
| 2 wt% Pd-BFO | 22.9 | 0.0046 | 1.5 | 105 | 90 | 7 | 0 | 5 | 3.82 | 23.6 | NaCl |
| 2 wt% Pd-BFO | 22.9 | 0.0046 | 1.5 | 105 | 120 | 7 | 0 | 5 | 3.41 | 31.8 | NaCl |
| 2 wt% Pd-BFO | 22.9 | 0.0046 | 1.5 | 105 | 150 | 7 | 0 | 5 | 3.08 | 38.4 | NaCl |
| 2 wt% Pd-BFO | 22.9 | 0.0046 | 1.5 | 105 | 180 | 7 | 0 | 5 | 2.7 | 46 | NaCl |
| 2 wt% Pd-BFO | 22.9 | 0.0046 | 1.5 | 105 | 210 | 7 | 0 | 5 | 2.41 | 51.8 | NaCl |
| 2 wt% Pd-BFO | 22.9 | 0.0046 | 1.5 | 105 | 240 | 7 | 0 | 5 | 2.04 | 59.2 | NaCl |
| 2 wt% Pd-BFO | 22.9 | 0.0046 | 1.5 | 105 | 270 | 7 | 0 | 5 | 1.79 | 64.2 | NaCl |
| 2 wt% Pd-BFO | 22.9 | 0.0046 | 1.5 | 105 | 0 | 7 | 0 | 5 | 5 | 0 | Na2SO4 |
| 2 wt% Pd-BFO | 22.9 | 0.0046 | 1.5 | 105 | 30 | 7 | 0 | 5 | 4.01 | 19.8 | Na2SO4 |
| 2 wt% Pd-BFO | 22.9 | 0.0046 | 1.5 | 105 | 60 | 7 | 0 | 5 | 3.45 | 31 | Na2SO4 |
| 2 wt% Pd-BFO | 22.9 | 0.0046 | 1.5 | 105 | 90 | 7 | 0 | 5 | 2.91 | 41.8 | Na2SO4 |
| 2 wt% Pd-BFO | 22.9 | 0.0046 | 1.5 | 105 | 120 | 7 | 0 | 5 | 2.3 | 54 | Na2SO4 |
| 2 wt% Pd-BFO | 22.9 | 0.0046 | 1.5 | 105 | 150 | 7 | 0 | 5 | 1.79 | 64.2 | Na2SO4 |
| 2 wt% Pd-BFO | 22.9 | 0.0046 | 1.5 | 105 | 180 | 7 | 0 | 5 | 1.22 | 75.6 | Na2SO4 |
| 2 wt% Pd-BFO | 22.9 | 0.0046 | 1.5 | 105 | 210 | 7 | 0 | 5 | 0.7 | 86 | Na2SO4 |
| 2 wt% Pd-BFO | 22.9 | 0.0046 | 1.5 | 105 | 240 | 7 | 0 | 5 | 0.19 | 96.2 | Na2SO4 |
| 2 wt% Pd-BFO | 22.9 | 0.0046 | 1.5 | 105 | 270 | 7 | 0 | 5 | 0 | 100 | Na2SO4 |
| 2 wt% Pd-BFO | 22.9 | 0.0046 | 1.5 | 105 | 0 | 7 | 0 | 5 | 5 | 0 | Na2SO4 |
| 2 wt% Pd-BFO | 22.9 | 0.0046 | 1.5 | 105 | 30 | 7 | 0 | 5 | 3.98 | 20.4 | Na2SO4 |
| 2 wt% Pd-BFO | 22.9 | 0.0046 | 1.5 | 105 | 60 | 7 | 0 | 5 | 3.47 | 30.6 | Na2SO4 |
| 2 wt% Pd-BFO | 22.9 | 0.0046 | 1.5 | 105 | 90 | 7 | 0 | 5 | 2.95 | 41 | Na2SO4 |
| 2 wt% Pd-BFO | 22.9 | 0.0046 | 1.5 | 105 | 120 | 7 | 0 | 5 | 2.34 | 53.2 | Na2SO4 |
| 2 wt% Pd-BFO | 22.9 | 0.0046 | 1.5 | 105 | 150 | 7 | 0 | 5 | 1.76 | 64.8 | Na2SO4 |
| 2 wt% Pd-BFO | 22.9 | 0.0046 | 1.5 | 105 | 180 | 7 | 0 | 5 | 1.19 | 76.2 | Na2SO4 |
| 2 wt% Pd-BFO | 22.9 | 0.0046 | 1.5 | 105 | 210 | 7 | 0 | 5 | 0.66 | 86.8 | Na2SO4 |
| 2 wt% Pd-BFO | 22.9 | 0.0046 | 1.5 | 105 | 240 | 7 | 0 | 5 | 0.22 | 95.6 | Na2SO4 |
| 2 wt% Pd-BFO | 22.9 | 0.0046 | 1.5 | 105 | 270 | 7 | 0 | 5 | 0 | 100 | Na2SO4 |
| 2 wt% Pd-BFO | 22.9 | 0.0046 | 1.5 | 105 | 0 | 7 | 0 | 5 | 5 | 0 | Na2SO4 |
| 2 wt% Pd-BFO | 22.9 | 0.0046 | 1.5 | 105 | 30 | 7 | 0 | 5 | 3.93 | 21.4 | Na2SO4 |
| 2 wt% Pd-BFO | 22.9 | 0.0046 | 1.5 | 105 | 60 | 7 | 0 | 5 | 3.4 | 32 | Na2SO4 |
| 2 wt% Pd-BFO | 22.9 | 0.0046 | 1.5 | 105 | 90 | 7 | 0 | 5 | 2.84 | 43.2 | Na2SO4 |
| 2 wt% Pd-BFO | 22.9 | 0.0046 | 1.5 | 105 | 120 | 7 | 0 | 5 | 2.25 | 55 | Na2SO4 |
| 2 wt% Pd-BFO | 22.9 | 0.0046 | 1.5 | 105 | 150 | 7 | 0 | 5 | 1.7 | 66 | Na2SO4 |
| 2 wt% Pd-BFO | 22.9 | 0.0046 | 1.5 | 105 | 180 | 7 | 0 | 5 | 1.15 | 77 | Na2SO4 |
| 2 wt% Pd-BFO | 22.9 | 0.0046 | 1.5 | 105 | 210 | 7 | 0 | 5 | 0.6 | 88 | Na2SO4 |
| 2 wt% Pd-BFO | 22.9 | 0.0046 | 1.5 | 105 | 240 | 7 | 0 | 5 | 0.14 | 97.2 | Na2SO4 |
| 2 wt% Pd-BFO | 22.9 | 0.0046 | 1.5 | 105 | 270 | 7 | 0 | 5 | 0 | 100 | Na2SO4 |
| 2 wt% Pd-BFO | 22.9 | 0.0046 | 1.5 | 105 | 0 | 7 | 0 | 5 | 5 | 0 | NaCO3 |
| 2 wt% Pd-BFO | 22.9 | 0.0046 | 1.5 | 105 | 30 | 7 | 0 | 5 | 1.7 | 66 | NaCO3 |
| 2 wt% Pd-BFO | 22.9 | 0.0046 | 1.5 | 105 | 60 | 7 | 0 | 5 | 1.14 | 77.2 | NaCO3 |
| 2 wt% Pd-BFO | 22.9 | 0.0046 | 1.5 | 105 | 90 | 7 | 0 | 5 | 0.72 | 85.6 | NaCO3 |
| 2 wt% Pd-BFO | 22.9 | 0.0046 | 1.5 | 105 | 120 | 7 | 0 | 5 | 0.36 | 92.8 | NaCO3 |
| 2 wt% Pd-BFO | 22.9 | 0.0046 | 1.5 | 105 | 150 | 7 | 0 | 5 | 0.02 | 99.6 | NaCO3 |
| 2 wt% Pd-BFO | 22.9 | 0.0046 | 1.5 | 105 | 180 | 7 | 0 | 5 | 0 | 100 | NaCO3 |
| 2 wt% Pd-BFO | 22.9 | 0.0046 | 1.5 | 105 | 210 | 7 | 0 | 5 | 0 | 100 | NaCO3 |
| 2 wt% Pd-BFO | 22.9 | 0.0046 | 1.5 | 105 | 240 | 7 | 0 | 5 | 0 | 100 | NaCO3 |
| 2 wt% Pd-BFO | 22.9 | 0.0046 | 1.5 | 105 | 270 | 7 | 0 | 5 | 0 | 100 | NaCO3 |
| 2 wt% Pd-BFO | 22.9 | 0.0046 | 1.5 | 105 | 0 | 7 | 0 | 5 | 5 | 0 | NaCO3 |
| 2 wt% Pd-BFO | 22.9 | 0.0046 | 1.5 | 105 | 30 | 7 | 0 | 5 | 1.77 | 64.6 | NaCO3 |
| 2 wt% Pd-BFO | 22.9 | 0.0046 | 1.5 | 105 | 60 | 7 | 0 | 5 | 1.19 | 76.2 | NaCO3 |
| 2 wt% Pd-BFO | 22.9 | 0.0046 | 1.5 | 105 | 90 | 7 | 0 | 5 | 0.69 | 86.2 | NaCO3 |
| 2 wt% Pd-BFO | 22.9 | 0.0046 | 1.5 | 105 | 120 | 7 | 0 | 5 | 0.33 | 93.4 | NaCO3 |
| 2 wt% Pd-BFO | 22.9 | 0.0046 | 1.5 | 105 | 150 | 7 | 0 | 5 | 0.05 | 99 | NaCO3 |
| 2 wt% Pd-BFO | 22.9 | 0.0046 | 1.5 | 105 | 180 | 7 | 0 | 5 | 0 | 100 | NaCO3 |
| 2 wt% Pd-BFO | 22.9 | 0.0046 | 1.5 | 105 | 210 | 7 | 0 | 5 | 0 | 100 | NaCO3 |
| 2 wt% Pd-BFO | 22.9 | 0.0046 | 1.5 | 105 | 240 | 7 | 0 | 5 | 0 | 100 | NaCO3 |
| 2 wt% Pd-BFO | 22.9 | 0.0046 | 1.5 | 105 | 270 | 7 | 0 | 5 | 0 | 100 | NaCO3 |
| 2 wt% Pd-BFO | 22.9 | 0.0046 | 1.5 | 105 | 0 | 7 | 0 | 5 | 5 | 0 | NaCO3 |
| 2 wt% Pd-BFO | 22.9 | 0.0046 | 1.5 | 105 | 30 | 7 | 0 | 5 | 1.84 | 63.2 | NaCO3 |
| 2 wt% Pd-BFO | 22.9 | 0.0046 | 1.5 | 105 | 60 | 7 | 0 | 5 | 1.26 | 74.8 | NaCO3 |
| 2 wt% Pd-BFO | 22.9 | 0.0046 | 1.5 | 105 | 90 | 7 | 0 | 5 | 0.75 | 85 | NaCO3 |
| 2 wt% Pd-BFO | 22.9 | 0.0046 | 1.5 | 105 | 120 | 7 | 0 | 5 | 0.37 | 92.6 | NaCO3 |
| 2 wt% Pd-BFO | 22.9 | 0.0046 | 1.5 | 105 | 150 | 7 | 0 | 5 | 0.08 | 98.4 | NaCO3 |
| 2 wt% Pd-BFO | 22.9 | 0.0046 | 1.5 | 105 | 180 | 7 | 0 | 5 | 0 | 100 | NaCO3 |
| 2 wt% Pd-BFO | 22.9 | 0.0046 | 1.5 | 105 | 210 | 7 | 0 | 5 | 0 | 100 | NaCO3 |
| 2 wt% Pd-BFO | 22.9 | 0.0046 | 1.5 | 105 | 240 | 7 | 0 | 5 | 0 | 100 | NaCO3 |
| 2 wt% Pd-BFO | 22.9 | 0.0046 | 1.5 | 105 | 270 | 7 | 0 | 5 | 0 | 100 | NaCO3 |
| 2 wt% Pd-BFO | 22.9 | 0.0046 | 1.5 | 105 | 0 | 7 | 0 | 5 | 5 | 0 | NaHCO3 |
| 2 wt% Pd-BFO | 22.9 | 0.0046 | 1.5 | 105 | 30 | 7 | 0 | 5 | 3.39 | 32.2 | NaHCO3 |
| 2 wt% Pd-BFO | 22.9 | 0.0046 | 1.5 | 105 | 60 | 7 | 0 | 5 | 2.61 | 47.8 | NaHCO3 |
| 2 wt% Pd-BFO | 22.9 | 0.0046 | 1.5 | 105 | 90 | 7 | 0 | 5 | 1.94 | 61.2 | NaHCO3 |
| 2 wt% Pd-BFO | 22.9 | 0.0046 | 1.5 | 105 | 120 | 7 | 0 | 5 | 1.23 | 75.4 | NaHCO3 |
| 2 wt% Pd-BFO | 22.9 | 0.0046 | 1.5 | 105 | 150 | 7 | 0 | 5 | 0.63 | 87.4 | NaHCO3 |
| 2 wt% Pd-BFO | 22.9 | 0.0046 | 1.5 | 105 | 180 | 7 | 0 | 5 | 0.08 | 98.4 | NaHCO3 |
| 2 wt% Pd-BFO | 22.9 | 0.0046 | 1.5 | 105 | 210 | 7 | 0 | 5 | 0 | 100 | NaHCO3 |
| 2 wt% Pd-BFO | 22.9 | 0.0046 | 1.5 | 105 | 240 | 7 | 0 | 5 | 0 | 100 | NaHCO3 |
| 2 wt% Pd-BFO | 22.9 | 0.0046 | 1.5 | 105 | 270 | 7 | 0 | 5 | 0 | 100 | NaHCO3 |
| 2 wt% Pd-BFO | 22.9 | 0.0046 | 1.5 | 105 | 0 | 7 | 0 | 5 | 5 | 0 | NaHCO3 |
| 2 wt% Pd-BFO | 22.9 | 0.0046 | 1.5 | 105 | 30 | 7 | 0 | 5 | 3.42 | 31.6 | NaHCO3 |
| 2 wt% Pd-BFO | 22.9 | 0.0046 | 1.5 | 105 | 60 | 7 | 0 | 5 | 2.65 | 47 | NaHCO3 |
| 2 wt% Pd-BFO | 22.9 | 0.0046 | 1.5 | 105 | 90 | 7 | 0 | 5 | 1.97 | 60.6 | NaHCO3 |
| 2 wt% Pd-BFO | 22.9 | 0.0046 | 1.5 | 105 | 120 | 7 | 0 | 5 | 1.3 | 74 | NaHCO3 |
| 2 wt% Pd-BFO | 22.9 | 0.0046 | 1.5 | 105 | 150 | 7 | 0 | 5 | 0.66 | 86.8 | NaHCO3 |
| 2 wt% Pd-BFO | 22.9 | 0.0046 | 1.5 | 105 | 180 | 7 | 0 | 5 | 0.13 | 97.4 | NaHCO3 |
| 2 wt% Pd-BFO | 22.9 | 0.0046 | 1.5 | 105 | 210 | 7 | 0 | 5 | 0 | 100 | NaHCO3 |
| 2 wt% Pd-BFO | 22.9 | 0.0046 | 1.5 | 105 | 240 | 7 | 0 | 5 | 0 | 100 | NaHCO3 |
| 2 wt% Pd-BFO | 22.9 | 0.0046 | 1.5 | 105 | 270 | 7 | 0 | 5 | 0 | 100 | NaHCO3 |
| 2 wt% Pd-BFO | 22.9 | 0.0046 | 1.5 | 105 | 0 | 7 | 0 | 5 | 5 | 0 | NaHCO3 |
| 2 wt% Pd-BFO | 22.9 | 0.0046 | 1.5 | 105 | 30 | 7 | 0 | 5 | 3.35 | 33 | NaHCO3 |
| 2 wt% Pd-BFO | 22.9 | 0.0046 | 1.5 | 105 | 60 | 7 | 0 | 5 | 2.6 | 48 | NaHCO3 |
| 2 wt% Pd-BFO | 22.9 | 0.0046 | 1.5 | 105 | 90 | 7 | 0 | 5 | 1.9 | 62 | NaHCO3 |
| 2 wt% Pd-BFO | 22.9 | 0.0046 | 1.5 | 105 | 120 | 7 | 0 | 5 | 1.2 | 76 | NaHCO3 |
| 2 wt% Pd-BFO | 22.9 | 0.0046 | 1.5 | 105 | 150 | 7 | 0 | 5 | 0.6 | 88 | NaHCO3 |
| 2 wt% Pd-BFO | 22.9 | 0.0046 | 1.5 | 105 | 180 | 7 | 0 | 5 | 0.05 | 99 | NaHCO3 |
| 2 wt% Pd-BFO | 22.9 | 0.0046 | 1.5 | 105 | 210 | 7 | 0 | 5 | 0 | 100 | NaHCO3 |
| 2 wt% Pd-BFO | 22.9 | 0.0046 | 1.5 | 105 | 240 | 7 | 0 | 5 | 0 | 100 | NaHCO3 |
| 2 wt% Pd-BFO | 22.9 | 0.0046 | 1.5 | 105 | 270 | 7 | 0 | 5 | 0 | 100 | NaHCO3 |
| 2 wt% Pd-BFO | 22.9 | 0.0046 | 1.5 | 105 | 0 | 7 | 0 | 5 | 5 | 0 | Na2HPO4 |
| 2 wt% Pd-BFO | 22.9 | 0.0046 | 1.5 | 105 | 30 | 7 | 0 | 5 | 4.39 | 12.2 | Na2HPO4 |
| 2 wt% Pd-BFO | 22.9 | 0.0046 | 1.5 | 105 | 60 | 7 | 0 | 5 | 3.97 | 20.6 | Na2HPO4 |
| 2 wt% Pd-BFO | 22.9 | 0.0046 | 1.5 | 105 | 90 | 7 | 0 | 5 | 3.57 | 28.6 | Na2HPO4 |
| 2 wt% Pd-BFO | 22.9 | 0.0046 | 1.5 | 105 | 120 | 7 | 0 | 5 | 3.14 | 37.2 | Na2HPO4 |
| 2 wt% Pd-BFO | 22.9 | 0.0046 | 1.5 | 105 | 150 | 7 | 0 | 5 | 2.74 | 45.2 | Na2HPO4 |
| 2 wt% Pd-BFO | 22.9 | 0.0046 | 1.5 | 105 | 180 | 7 | 0 | 5 | 2.33 | 53.4 | Na2HPO4 |
| 2 wt% Pd-BFO | 22.9 | 0.0046 | 1.5 | 105 | 210 | 7 | 0 | 5 | 1.94 | 61.2 | Na2HPO4 |
| 2 wt% Pd-BFO | 22.9 | 0.0046 | 1.5 | 105 | 240 | 7 | 0 | 5 | 1.51 | 69.8 | Na2HPO4 |
| 2 wt% Pd-BFO | 22.9 | 0.0046 | 1.5 | 105 | 270 | 7 | 0 | 5 | 1.13 | 77.4 | Na2HPO4 |
| 2 wt% Pd-BFO | 22.9 | 0.0046 | 1.5 | 105 | 0 | 7 | 0 | 5 | 5 | 0 | Na2HPO4 |
| 2 wt% Pd-BFO | 22.9 | 0.0046 | 1.5 | 105 | 30 | 7 | 0 | 5 | 4.34 | 13.2 | Na2HPO4 |
| 2 wt% Pd-BFO | 22.9 | 0.0046 | 1.5 | 105 | 60 | 7 | 0 | 5 | 3.94 | 21.2 | Na2HPO4 |
| 2 wt% Pd-BFO | 22.9 | 0.0046 | 1.5 | 105 | 90 | 7 | 0 | 5 | 3.51 | 29.8 | Na2HPO4 |
| 2 wt% Pd-BFO | 22.9 | 0.0046 | 1.5 | 105 | 120 | 7 | 0 | 5 | 3.11 | 37.8 | Na2HPO4 |
| 2 wt% Pd-BFO | 22.9 | 0.0046 | 1.5 | 105 | 150 | 7 | 0 | 5 | 2.7 | 46 | Na2HPO4 |
| 2 wt% Pd-BFO | 22.9 | 0.0046 | 1.5 | 105 | 180 | 7 | 0 | 5 | 2.29 | 54.2 | Na2HPO4 |
| 2 wt% Pd-BFO | 22.9 | 0.0046 | 1.5 | 105 | 210 | 7 | 0 | 5 | 1.89 | 62.2 | Na2HPO4 |
| 2 wt% Pd-BFO | 22.9 | 0.0046 | 1.5 | 105 | 240 | 7 | 0 | 5 | 1.46 | 70.8 | Na2HPO4 |
| 2 wt% Pd-BFO | 22.9 | 0.0046 | 1.5 | 105 | 270 | 7 | 0 | 5 | 1.09 | 78.2 | Na2HPO4 |
| 2 wt% Pd-BFO | 22.9 | 0.0046 | 1.5 | 105 | 0 | 7 | 0 | 5 | 5 | 0 | Na2HPO4 |
| 2 wt% Pd-BFO | 22.9 | 0.0046 | 1.5 | 105 | 30 | 7 | 0 | 5 | 3.4 | 32 | Na2HPO4 |
| 2 wt% Pd-BFO | 22.9 | 0.0046 | 1.5 | 105 | 60 | 7 | 0 | 5 | 3.89 | 22.2 | Na2HPO4 |
| 2 wt% Pd-BFO | 22.9 | 0.0046 | 1.5 | 105 | 90 | 7 | 0 | 5 | 3.45 | 31 | Na2HPO4 |
| 2 wt% Pd-BFO | 22.9 | 0.0046 | 1.5 | 105 | 120 | 7 | 0 | 5 | 3.06 | 38.8 | Na2HPO4 |
| 2 wt% Pd-BFO | 22.9 | 0.0046 | 1.5 | 105 | 150 | 7 | 0 | 5 | 2.65 | 47 | Na2HPO4 |
| 2 wt% Pd-BFO | 22.9 | 0.0046 | 1.5 | 105 | 180 | 7 | 0 | 5 | 2.25 | 55 | Na2HPO4 |
| 2 wt% Pd-BFO | 22.9 | 0.0046 | 1.5 | 105 | 210 | 7 | 0 | 5 | 1.85 | 63 | Na2HPO4 |
| 2 wt% Pd-BFO | 22.9 | 0.0046 | 1.5 | 105 | 240 | 7 | 0 | 5 | 1.4 | 72 | Na2HPO4 |
| 2 wt% Pd-BFO | 22.9 | 0.0046 | 1.5 | 105 | 270 | 7 | 0 | 5 | 1.05 | 79 | Na2HPO4 |
